# Supplementary material for: Neuro-computational mechanisms and individual biases in action-outcome learning under moral conflict
Source: Nat Commun. 2023 Mar 6;14:1218. doi: 10.1038/s41467-023-36807-3 (PMC9988878; doi:10.1038/s41467-023-36807-3)
Supplement: Supplementary file 1 — Supplementary Information [file 41467_2023_36807_MOESM1_ESM.pdf]

Supplementary Notes for

Neuro-computational mechanisms and individual biases in action-outcome learning under moral conflict

Laura Fornari[1§], Kalliopi Ioumpa[1§], Alessandra D. Nostro[1], Nathan J. Evans[2], Lorenzo De Angelis[1], Sebastian P. H. Speer[1], Riccardo Paracampo[1], Selene Gallo[1], Michael Spezio[3], Christian Keysers[1,4,#] & Valeria Gazzola[1,4,#,*]

[§,#] equal contribution
* corresponding author: v.gazzola@nin.knaw.nl

Table of Content

Supplementary Note 1. Stimuli creation and validation
Supplementary Note 2. General overview of the experimental procedures
Supplementary Fig. 1. Experimental procedures
Supplementary Note 3. Helping Task
Supplementary Fig. 2. Helping task structure
Supplementary Note 4. Optimization task
Supplementary Table 1. Optimization Task
Supplementary Note 5. Participants' belief that someone will get shocks
Supplementary Table 2. Distribution of participants' beliefs.
Supplementary Fig. 3. Effect of belief … on choice allocation in the Online study
Supplementary Note 6. Participants' motivation
Supplementary Fig. 4. Motivation as a function of preference.
Supplementary Table 3: Descriptive statistics for the Pain-Avoidance score.
Supplementary Note 7. Choices allocation across conditions
Supplementary Fig. 5. Choice allocation in Dropout and NoDropout Conflict Online conditions
Supplementary Note 8. Choices across all 20 trials
Supplementary Fig. 6. Participant's choices in the Dropout blocks.
Supplementary Note 9. Explicit Report
Supplementary Table 4. Differences in Reported Probabilities.
Supplementary Table 5. Comparison in Reported Probabilities.
Supplementary Note 10. Model comparison in ConflictDropout blocks
Supplementary Fig. 7. Model Comparison.
Supplementary Fig. 8. M2 with weighting at both Decision and Outcome (M2DO).
Supplementary Note 11. Age and gender differences across studies
Supplementary Fig. 9. Gender and age differences across studies.
Supplementary Note 12. Ambiguous group characterization
Supplementary Fig. 10. Choices over blocks.
Supplementary Fig. 11. Simulated choices variance.
Supplementary Fig. 12. Learning parameters across groups.
Supplementary Fig. 13. Reaction time across groups.

Supplementary Fig. 14. Explicit recall comparison across groups.
Supplementary Note 13. M2Out Parameter Distributions and Recovery
Supplementary Fig. 15. M2Out Parameter Distributions and Recovery
Supplementary Fig. 16: Correlations between LR and wf.
Supplementary Note 14. Predicting helping based on wf but not IRI or MAS
Supplementary Table 6. Bayesian linear regression posterior summary of coefficients.
Supplementary Note 15. How to generate fMRI parameter estimates that can be easily interpreted.
Supplementary Table 7: Impact of dividing PES by (1-wf).
Supplementary Note 16. Additional fMRI results.
Supplementary Table 8. BOLD activity associated with the main effect of Outcome
Supplementary Table 9: Supplementary Signature Analyses
Supplementary Table 10. BOLD activity covarying positively with PES
Supplementary Table 11. BOLD activity covarying with PES in a way that depends linearly on wf.
Supplementary Table 12. BOLD activity covarying positively with PEM
Supplementary Table 13. BOLD activity covarying with PEM in a way that depends linearly on wf
Supplementary Table 14. BOLD activity covarying with PES in a way that depends linearly on LRS
Supplementary Fig. 17. Overlap between PEM and PES.
Supplementary Fig. 18. Decision phase and EV.
Supplementary Table 15. BOLD activity associated with the main effect of Decision.
Supplementary Table 16. BOLD activity covarying positively with -EVS
Supplementary Note 17. PEM and PES separability
Supplementary Table 17. Frequentist tests for simulations without wf dependence.
Supplementary Table 18. Frequentist tests with or without wf dependence.
Supplementary Table 19. Bayesian tests for simulations without wf dependence
Supplementary Table 20. Bayesian tests with or without wf dependence.
Supplementary Note 18. Two-steps procedure
Supplementary Fig. 19: Dissociating Outcome vs Prediction Error coding.
Supplementary Note 19. Psychological description of the difference between our learning models
Supplementary Note 20. Stress Tolerance short questionnaire (STSQ), and association with wf
Supplementary Note 21. Pain rating, pre- and post-disclosure feedback questionnaires and associations with wf
Supplementary Fig. 20: Multiple regression explaining wf using Q4 and Q8 of the pre-disclosure feedback questionnaire.
Supplementary Note 22. Eye gaze analysis
Supplementary Fig. 21.Correlation between time spent looking at the video and considerate choices.

Supplementary Note 1. Stimuli creation and validation

Videos

Videos were generated in house. The authors SG and AN played the role of the actress during the fMRI and Online experiment respectively. All videos were recorded to start with an initial neutral facial expression, followed by the facial expression in response to the electrical stimulation delivered to the right-hand dorsum. The upper part of the actress' body was clearly visible on a black background. The actresses were encouraged to produce realistic and clear facial expressions in response to the temporally unpredictable stimulations. The intensity of the stimulation was decided with the actresses, and the facial expressions were exaggerated to reflect a clear expression of pain. All original recordings were cut to last 2s and to have the frame showing the beginning of the facial expression in response to the stimulation exactly at 1s. All videos were validated by independent groups of subjects, who were asked to rate the intensity of the pain experienced by the confederate on a scale from 1 to 10, with '1' being 'just a simple touch sensation' and '10' being 'most intense imaginable pain'. As low pain intensity videos we selected the ones with ratings of 1 and 2 and as high pain intensity videos we selected the ones with ratings of 4, 5 and 6 Videos were edited using Adobe Premiere Pro CS6 (Adobe, San Jose, CA, USA).

The videos used for the Learning task in the fMRI experiment were selected from the Hand videos recorded for Gallo et al., 2018 [4] (https://elifesciences.org/articles/32740, see also Supplementary Note§3 below, and https://doi.org/10.7554/eLife.32740.006, https://doi.org/10.7554/eLife.32740.009). Thirteen videos of value 1 and 14 of value 2 were used as low pain stimuli, while 9 videos of value 4, 13 of value 5 and 3 of value 6 were chosen for as high intensity stimuli. A full description of how these videos were created can be found in [4]. A similar procedure was then used to generate the video of the Online experiment (for two examples see https://osf.io/rk8w4/?view_only=98b193a58aff48dda40b9d3d91ac5254 Figure1_Task). The main difference was that in the stimuli for the Online experiment both actress's hands were shown (Figure 1a), instead of only the right hand. For the Online experiment, an initial pool of 300 videos showing a painful stimulation and 300 videos showing an innocuous stimulation were recorded. The final pool of stimuli was then selected based on an online validation (survey done through Gorilla, https://gorilla.sc/) performed by 191 participants (aged 18 – 35, 103 females). Participants were recruited through Prolific (https://prolific.ac/). From the initial pool, 140 high intensity and 130 low intensity stimuli were selected. Since for each condition we needed 140 trials (120 trials with both outcomes plus 40 trials with money dropout), 10 low videos were repeated twice in each condition. The exact same videos used in the Conflict blocks were repeated in the NoConflict blocks, this allowed us to rule-out any effect due to the difference in the displayed stimuli between conditions. The repetition did not have any negative impact on participants' performance, since in the Online experiment participants were aware of the fact that these videos had been pre-recorded.

The high intensity pain stimuli used in the fMRI Learning task had an average rating of 4.76 ± 0.66SD and the low intensity stimuli of 1.52 ± 0.51SD (Bayesian Mann-Whitney *t*-tests W=675, $p<0.001$, $BF_{10}=2710.5$). For the Online study, the high intensity pain stimuli had an average rating of 5.74 ± 0.47SD and the low intensity stimuli of 1.54 ± 0.18SD (Bayesian Mann-Whitney t-tests, W=18200, $BF_{10}=7.7*10^{11}$, $p<0.001$). There was no difference between the average rating of the low intensity pain stimuli used in the fMRI and in the Online experiment (Mann-Whitney *t*-test W=1750, $BF_{10}=0.24$, $p=0.983$). On the other hand, the difference between the average rating of the high intensity pain stimuli used in the two experiments was significant (W=502.50, $BF_{10}=456.31$, $p<0.001$). Having high intensity pain stimuli perceived as of lower intensity in the fMRI experiment might a priori predict more lucrative preferences in the fMRI compared to the Online experiment. In contrast, lucrative preferences were rarer in the fMRI than in the Online experiment (Table 1), suggesting that this difference in the high intensity stimuli did not impact behaviour substantially.

Symbols

All the symbols used in the learning task were created using Adobe Illustrator. For the creation of symbols we used simple geometrical shapes without obvious meaning. Each symbol was then paired with a second one created using the same shape elements organized in a different position. All the symbols covered the same area on the screen. Twenty-four pairs of symbols were used in the test: 12 pairs were used in the Conflict, and 12 in the NoConflict condition. The absence of a priori preference for a symbols in each pair was assessed in an independent group of subjects (Bayesian Wilcoxon Signed-Rank test, W=31, BF_{10}=0.31, *p*=0.547).

Supplementary Note 2. General overview of the experimental procedures

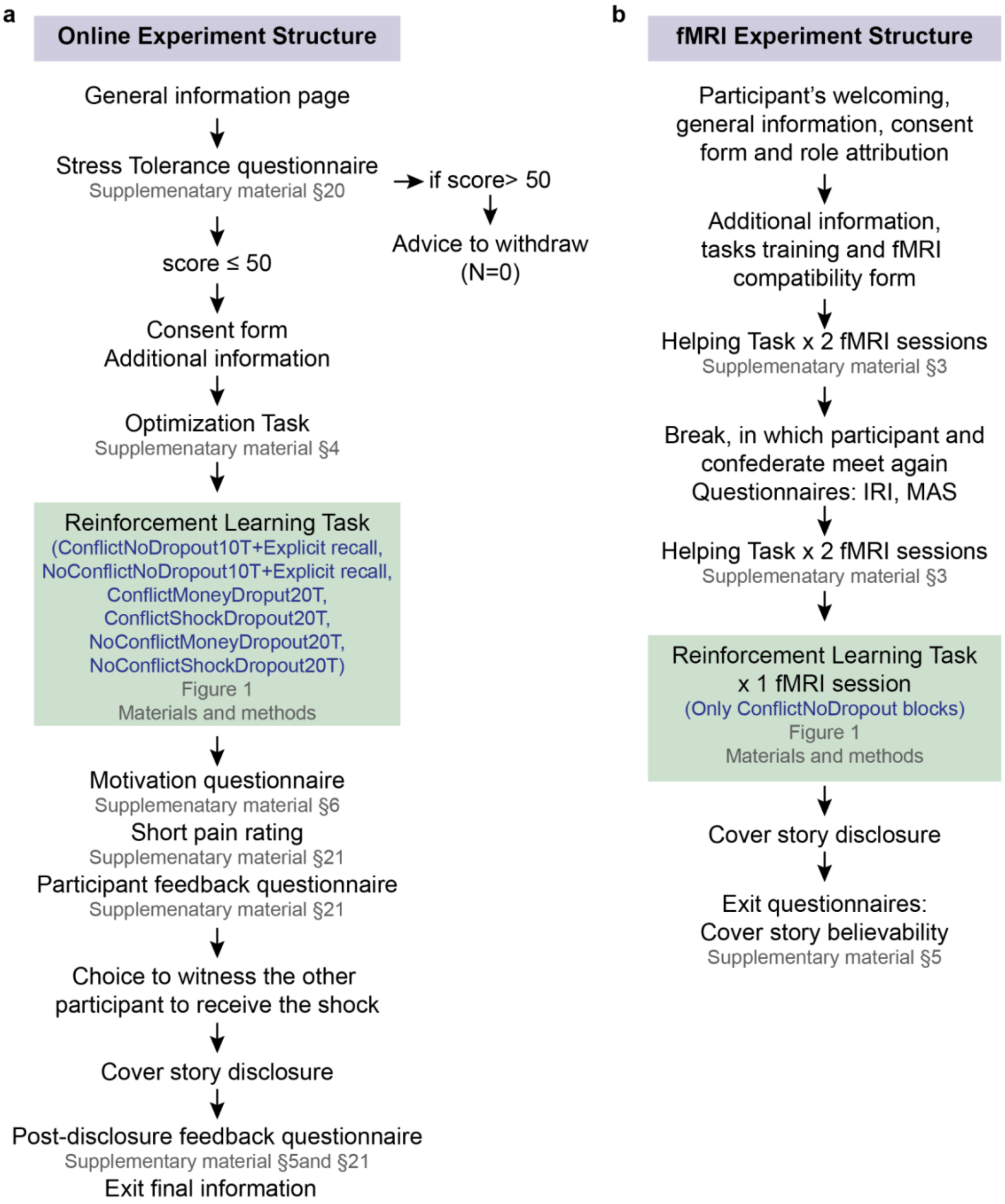

Supplementary Fig. 1. Experimental procedures. For both the Online (a) and fMRI (b) experiments the schema details the sequence of procedures, tasks (with included conditions) and questionnaires each participant went through.

Supplementary Note 3. Helping Task

In the fMRI experiment, participants performed the Helping Task presented in Gallo et al. [4] as the main experimental task. Only the behavioral results (i.e., average donation per participant associated with the Face stimuli) of the Helping Task are included in the current publication. Briefly, participants performed 60 trials in which they watched a first (pre-recorded) video of the same confederate as in the Learning Task receiving a painful stimulation. The intensity of the stimulation could vary between 1 and 6 on a 10-point pain scale, and was chosen on each trial by the computer program. In each trial participants also receive 6 credits, and could decide to donate some of these credits to reduce the intensity of the second stimulation to the confederate. Each credit donated back to the experimenter reduced the next stimulation by 1 point on the 10-point pain scale. Participants then watched a second video showing the confederate's response to the second stimulation. At the end of the task, participants were paid the sum of the amount of money that they had kept for themselves from all the trials divided by 10. We capture individual differences as the average number of credits given up per trials ("donation"). Two types of videos were presented. One showing the confederate receiving an electroshock on the hand and expressing the pain she felt by only reacting with facial expression. The other showed a belt hitting the dorsum of the confederate right hand, and the confederate expressing how much pain she felt by a reaction of the hand alone. The face was not visible in the latter stimuli. Hand and Face videos were presented in separate sessions.

In total there were 2 sessions of 15 trials each presenting the hand video and 2 presenting the face. The single session with 6 blocks of Learning task was presented at the end, after the four Helping Task sessions. This was because the fMRI experiment was centered on the Helping task, and the Learning task was meant as a pilot data, which should have been followed by a second fMRI data acquisition centered to the Learning, which was however impossible due to COVID19 restrictions.

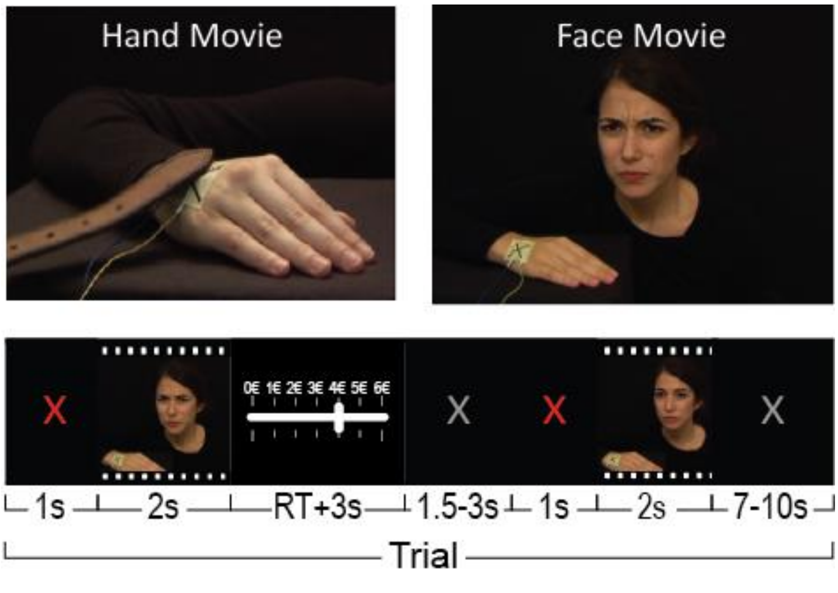

Supplementary Fig. 2. Helping task structure. Top: two screen shots taken from one exemplar video showing the confederate receiving the slap on her right hand, and from a video showing the confederate receiving the shock on the hand and manifesting its painfulness through facial reactions. Bottom: trial structure. For illustrative purposes, reaction times (RT).

Supplementary Note 4. Optimization task

In the Online experiment, before the learning tasks, we determined the amount of monetary reward that would have a subjective value equivalent to the painful shock received by the other participant. This task enabled us to personalize the amount of money participants were later offered as high reward in the learning tasks to create a meaningful conflict. Participants always had to choose between a considerate option combining 0.5€ for them with a low shock to the confederate, and a lucrative option combining a higher amount of money for themselves with a high shock to the confederate. The amount of money offered in the lucrative option varied in steps of 0.25€ across the 5 types of choices (see the table below). Before making the choice, the participant was able to see the intensity of the electrical stimulation for the confederate by playing pre-recorded videos.

	Considerate option	Lucrative option	Nr participants
1	0.5€ + Low Shock	1€ + High Shock	12 (6L, 3A, 3C)
2	0.5€ + Low Shock	1.25€ + High Shock	22 (12L, 8A, 2C)
3	0.5€ + Low Shock	1.5€ + High Shock	13 (3L, 7A, 3C)
4	0.5€ + Low Shock	1.75€ + High Shock	7 (2L, 2A, 3C)
5	0.5€ + Low Shock	2€ + High Shock	25 (1L, 6A, 18C)

Supplementary Table 1. Optimization Task. The table lists the five possible options offered during the optimization task, with the number of participants to which each option was assigned during the Learning Task of the Online experiment. The numbers in brackets indicate the number of participants showing a Lucrative (L), Considerate (C) or Ambiguous (A) preference during the task.

Each of the 5 types of choices were presented 6 times, for a total of 30 decisions. A sigmoid was then fitted to the choice data, and the indifference point was selected based on where the sigmoid crosses the 0.5 considerate proportion. To select the high reward to offer during the learning task, we picked the lowest value for which each participant chose half of the times the low-electrical stimulation option and half the high electrical stimulation option. In case there was not a value for which the number of considerate choices was equal to the number of lucrative choices, we picked the lowest value for which the number of lucrative choices was higher than the number of considerate choices. If a participant had always chosen the considerate option, we picked the highest value (2€) as the high reward for the learning experiment. Conversely, if a participant had always chosen the lucrative option, we picked the lowest value (1€) as the high reward for the learning experiment. Due to an error in programming the task, to 4 participants who had 1€ as real indifference, 1.25€ was offered as a high reward during learning. Another subject, whom should have been offered 1€ got offered 2€ as a high reward. This could have induced a more lucrative behavior in this subject; however this participant was still in the Considerate group, meaning that this didn't have a strong impact on performance. In the Online experiment, the average indifference point was 1.53 ± 0.37SD, which matched the 1.5 € given as high reward to every participant in the fMRI experiment.

Supplementary Note 5. Participants' belief that someone will get shocks

At the end of the Online study, participants were asked to express the degree of agreement to the statement: "Before choosing whether or not to see the other person receiving the electrical stimulations, I believed that someone was really going to receive them" on a scale from 1 (strongly disagree) to 7 (strongly agree). If they at least somewhat disagreed, they were additionally asked whether they nevertheless acted as if they believed someone would get shocks or not. An ANOVA with level of agreement as a nominal factor showed that average choices were not influenced by how much a participant believed in shock delivery (Main effect of belief in shocks, $F_{(6,72)}$=0.739, p=0.62, BF_{incl}=0.109). However, of the 28 Non-Believers (i.e. those who responded 'somewhat disagree', 'disagree', or 'strongly disagree'), 24 reported they nevertheless acted as if someone were to receive the shocks (Supplementary Table 2). This might explain why people that doubted anyone would get shocks would nevertheless give up money to reduce shocks. In contrast, the 4 participants reporting they acted as if no one were to get shocks (red in Supplementary Fig. 2) chose considerate options on average significantly less than the other 75 participants (Independent sample test $t_{(77)}$=-2.97, p=0.004, BF_{10}=8.75). In the fMRI study, all 27 participants reported that they believed that someone was getting a shock.

	**Total Nr of participants**	**Believers**	**Non-Believers**	
			Acting as if believing	Non-acting as if believing
**Online participants**	79	51	24	4
**fMRI participants**	27	27	0	0

Supplementary Table 2. Distribution of participants' beliefs.
The number in each cell is the participants count. Believers were considered those reporting values from 4 to 7 (neither agree to totally agree), non-believers those reporting 3 to 1 (somewhat disagree to totally disagree). Nr=number. Source data are provided as a Source Data file.

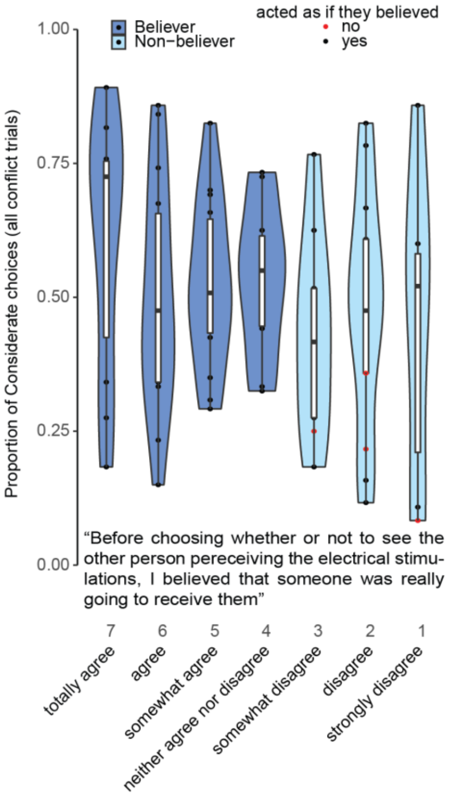

Supplementary Fig. 3. Effect of belief in the fact that the victim will receive shocks on choice allocation in the Online study. The figure shows the proportion of pain-reducing choices across all the conflict trials of the Online experiment (including No-Dropout and the 10 first trial of Dropout blocks) as a function of the answer provided. Violin plots represent the value distribution, the box-plot within, the median and quartiles, the whiskers, the range of datapoints between Q1-1.5IQR and Q3+1.5IQR, and red dots participants reporting that they did not act as if someone were getting shocks. Participants responding that they at least somewhat disagree are considered Non-Believers for the purpose of Supplementary Fig. 5. N=79. Believers in dark blue and Non-Believers in light blue. Source data are provided as a Source Data file.

Supplementary Note 6. Participants' motivation

To better understand what drove participants' choices in the Online learning task, at the end of the Online task, participants were asked the question "What motivated your choices?". Five options were proposed to the participant and they could choose to select 1 or more. In the case they selected more than 1, they were asked to select them in order of importance, starting from the most important. The options presented to participants were the following:

A) Gaining as much money as possible
B) Giving a good impression
C) Being in line with my moral beliefs
D) Avoiding to see the other person receiving the shocks
E) Preventing harm to others

Supplementary Fig. 4a below reports how often a particular motivation was chosen as the primary motivation in each group.

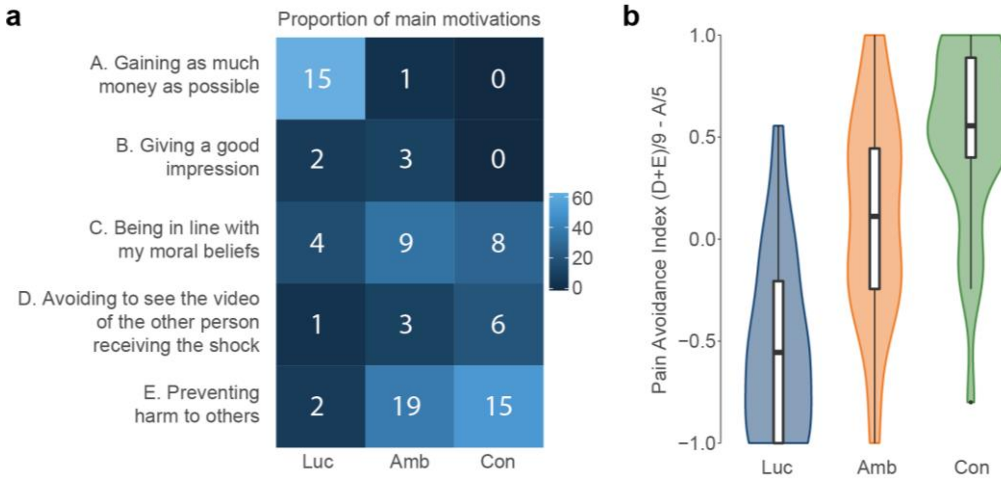

Supplementary Fig. 4.
Motivation as a function of preference. **a** for each of the preference groups, the color indicates the proportion of participants that have chosen a particular motivation as their primary motivation, and the white numbers represent the actual number of participants in that cell. N=79. **b** The value of the pain-avoidance index, calculated as (D+E)/9-A/5, as a function of group preference. Violin plots represent the value distribution, the box-plot within, the median and quartiles, the whiskers, the range of datapoints between Q1-1.5IQR and Q3+1.5IQR. This index has the value of +1 if participants select pain avoiding motivations as their first two motivations (i.e. preventing harm to others and avoiding to see the video of the other person receiving the shocks) and do not select 'gaining as much money as possible', and -1 if they select 'gaining as much money as possible' and not the two pain avoiding motivations. N=29 Considerate (Con, green), N=24 Lucrative (Luc, blue), N=26 Ambiguous (Amb, orange). Source data are provided as a Source Data file.

Since there were 5 alternatives, to quantify motivation, values from 0 to 5 were assigned to A-E according to its position in the list of motivations (0 if it had not been selected, 5 if it had been indicated as first motivation, 4 as second, 3 as third, 2 as forth and 1 as fifth). To assess whether the motivation overall was more lucrative or considerate, we then derived a secondary measure of '*Pain-Avoidance*' that would be negative if the motivation was mainly 'gaining as much money as possible' and positive if the motivation was 'preventing harm to others' or 'avoiding to see the other person receiving the shocks'. Specifically, this was calculated as (D+E)/9-A/5. We divided D+E by 9 and A by 5, because a person with maximum pain reducing motivation could choose D and E as their first motivations and thus obtain 5+4=9, and one maximally lucrative would choose A as their first motivation. We decided on this contrast of D+E and A, because we found D+E

to correlate highly negatively with A (tau=-0.38, p<0.001, BF_{10}=29232.57). Supplementary Table 3 below gives an overview of the average pain avoidance score calculator for the Lucrative, Considerate and Ambiguous group preferences separately (see also Supplementary Fig. 4b).

	**Pain-Avoidance score ((D+E)/9-A/5)**		
	**Lucrative**	**Ambiguous**	**Considerate**
Participants	24	26	29
Mean	-0.519	0.120	0.506
Std. Deviation	0.447	0.509	0.440
Shapiro-Wilk	0.903	0.960	0.887
P-value of Shapiro-Wilk	0.025	0.384	0.005
Minimum	-1.000	-1.000	-0.800
Maximum	0.556	1.000	1.000

Supplementary Table 3.
Descriptive statistics for the Pain-Avoidance score. As can be seen, the score indeed varies all the way from -1 to 1 amongst our participants, but is not normally distributed, and is thus analyzed non-parametrically. Source data are provided as a Source Data file.

To test whether participants that chose the considerate options during the task, within or below chance (i.e. considerate, neutral and lucrative) differed in their pain avoidance score, we performed a Bayesian ANOVA with group as factor. This analysis revealed a significant main effect of group (Kruskal Wallis tests, $H_{(2)}$=35.18, p<0.001,) and post-hoc Bayesian Mann-Whitney t-tests confirmed differences between all the 3 groups (Considerate-Ambiguous: W=196.50, p=0.002, BF_{10}=9.31; Lucrative-Ambiguous: W=111.50, p<0.001, BF_{10}=38.14; Lucrative-Considerate: W=47.50, p<0.001, BF_{10}=397.01).

Testing each value against zero using a Wilcoxon signed rank test showed that for the considerate preference group values were significantly above zero (W=380, p<0.001, BF_{10}=714.32), for the lucrative preference group they were below zero (W=13.5, p<0.001, BF_{10}=748.88) but for the ambiguous preference group, they were close to zero (W=227.5, p=0.19, BF_{10}=0.48). This suggests that participants in the Considerate and Lucrative group could and decided to consciously report motivations that were in line with their choices, while the Ambiguous group appeared to have less clearly polarized monetary or pain-avoiding motivations.

Supplementary Note 7. Choices allocation across conditions

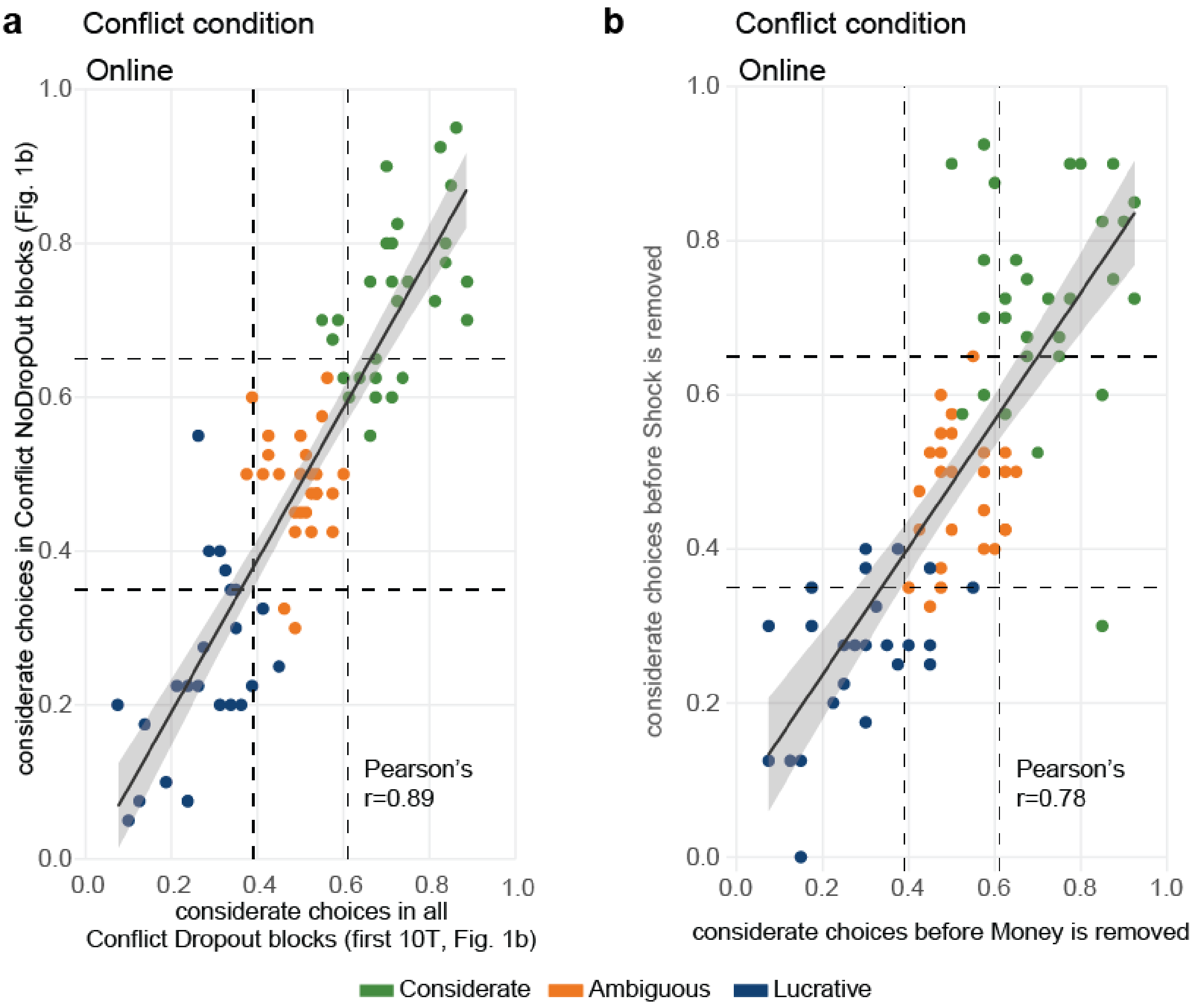

Supplementary Fig. 5. **Choice allocation in Dropout and NoDropout Conflict Online conditions. a** Average choices allocation per participant (N=29 Considerate in green, N=24 Lucrative in blue, N=26 Ambiguous in orange) during the first 10 trials of the 8 blocks that will later lead to Dropout (x-axis) and the 4 conflict NoDropout blocks (y-axis). Pearson's correlation= 0.89, two-tail p<3.848e-28, BF_{10}>1000; mean NoDropout=0.501±0.025s.e.m.; mean Dropout=0.512±0.023s.e.m.; Paired sample two-tail test $t_{(78)}$= 1.04, p=0.302; BF_{10}=0.208. Color code represents the subgroup classification including all trials. The dashed lines represent the critical values for the binomial distribution p<0.05 separately for the Dropout and NoDropout blocks. Regression line in black with shaded the 95% confidence interval. **b** As in **a** for the first 10 trials of the 4 Dropout blocks in which money will later be removed (x-axis), and the 4 Dropout blocks in which shock will later be removed (y-axis). Pearson's correlation=0.78, two-tail p=1.434e-17, BF_{10}>1000; mean MoneyDropout=0.52±0.024s.e.m.; mean ShockDropout=0.503±0.025s.e.m.; Paired sample two-tail test $t_{(78)}$=1.21, p=0.23, BF_{10}=0.25. Source data are provided as a Source Data file.

Supplementary Note 8. Choices across all 20 trials

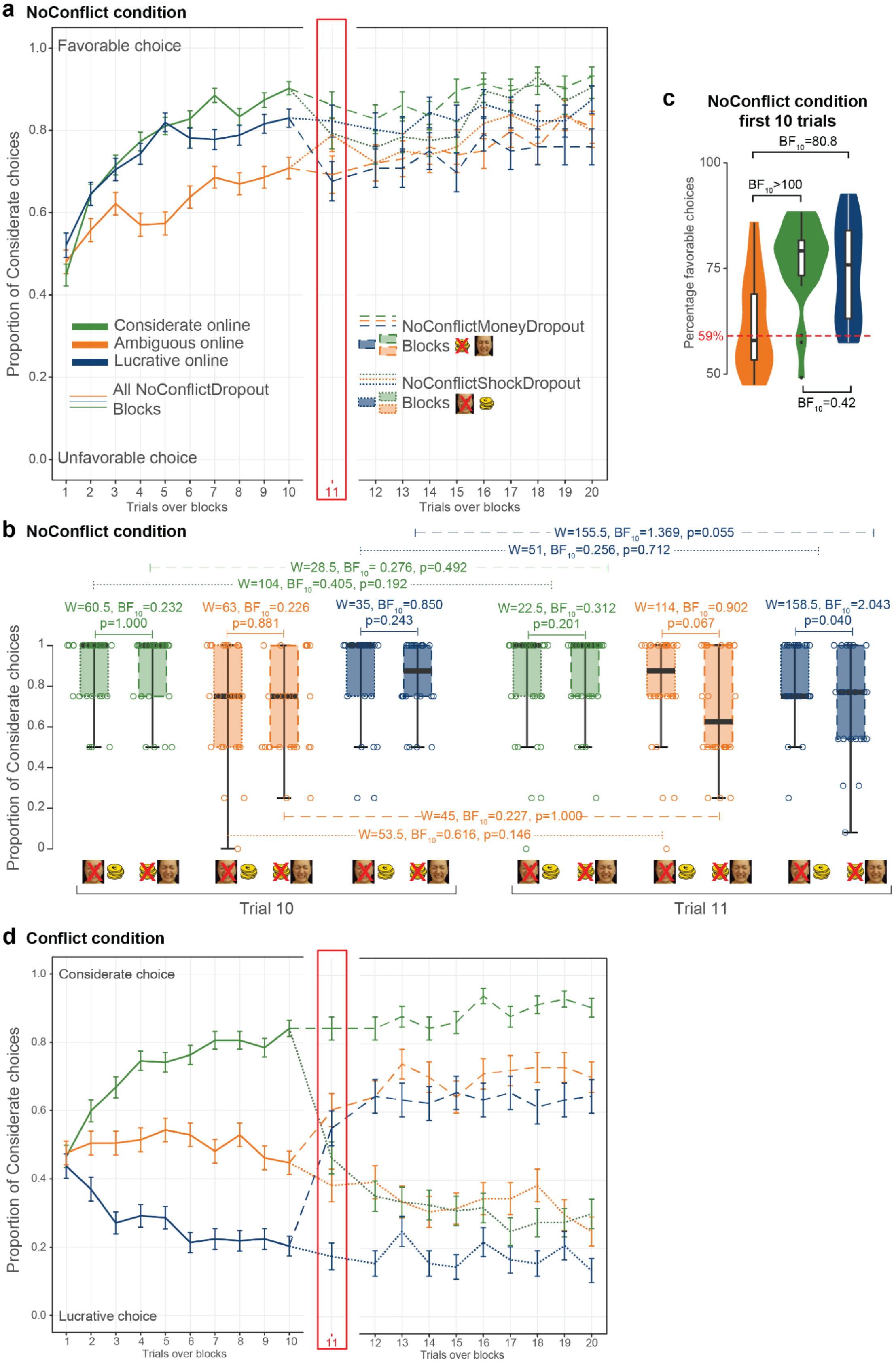

Supplementary Fig. 6. Participant's choices in the Dropout blocks. **a** For the NoConflict condition, the average ± sem considerate choices as a function of trial, separately for the three preference groups. Note that preference grouping is based on all NoConflict trials, i.e. including the NoDropout blocks, while the averages of proportion of considerate choices only include choices on the Dropout trials. Here we also show choices on trials 12-20 after Dropout. These choices are less relevant to the main paper, because they capture what is probably a different form of learning, when symbols are only associated with 1 outcome rather than 2

outcomes that can conflict. **b** NoConflict choices at the 10th and 11th trials, separately for condition and group. Dots are participants' proportions of considerate choices, Box-plot show the median and quartiles, with whiskers showing the range of datapoints between Q1-1.5IQR and Q3+1.5IQR. Points outside that range are shown as outliers. Numbers on top of box plots indicate the two-tail Wilcoxon signed-rank values from comparing choices at the 10th and 11th trials, BF_{10} and *p*-values. $BF_{10}>3$, evidence for the presence of a difference, and $BF_{10}<⅓$ evidence against. **c** Proportion of favorable choices (i.e. choosing the symbol most likely to lead to high-money and low-shock) for the first 10 trials of the NoConflictDropout condition, separately for the three groups. Violin plots represent the value distribution, the box-plot within, the median and quartiles, the whiskers, the range of datapoints between Q1-1.5IQR and Q3+1.5IQR. One way ANOVA, $F_{(2,76)}=15.6$, $p<0.001$; BF_{incl} group=8284.52. Red dotted line: learning threshold determined using binomial distribution (71/120 correct choices). 54% of the ambiguous preference subjects fell below this learning threshold, while just 7% of those with considerate and 4% of those with lucrative preference were below this threshold. **d** Same as in a and in Figure 4a, but for the Conflict condition, with the purpose to illustrate choices up to trial 20. N=79 (29 Considerate in green, 24 Lucrative in blue, 26 Ambiguous in orange) for all panels. Source data are provided as a Source Data file.

Supplementary Note 9. Explicit Report

One Sample T-Test

	(symbol) − (symbol)	**Average±SD Median [25% 75%]**	**Test**	**Stat**	**df**	***p***	**BF_{10}**	**Cohen's *d***
**Considerate**	Conflict Money	-20.78±19.21	Student	-5.83	28	2.93E-06	6556.565	-2.20
		-20 [-34 -2.25]	Wilcoxon	29.5		5.02E-05	194.196	
	Conflict Shock	-38.97±19.31	Student	-10.87	28	1.48E-11	6.09E+08	-4.11
		-39.25 [-52.50 -24.75]	Wilcoxon	0		4.00E-06	1.12E+08	
	NoConflict Money	27.36±23.52	Student	6.27	28	8.97E-07	19559.464	2.37
		24.25 [16.5 44]	Wilcoxon	415		1.38E-06	403.178	
	NoConflict Shock	-44.98±16.55	Student	-14.64	28	1.20E-14	5.38E+11	-5.53
		-48.00 [-54.25 -35.75]	Wilcoxon	0		2.70E-06	1498.691	
**Lucrative**	Conflict Money	-36.79±19.62	Student	-9.18	23	3.71E-09	3.24E+06	-3.83
		-40.25 [-51.62 -21.69]	Wilcoxon	3		2.84E-05	1529.834	
	Conflict Shock	-27.60±21.79	Student	-6.21	23	2.47E-06	7783.492	-2.59
		-23.75 [-38.69 -11.31]	Wilcoxon	4		8.35E-07	992.344	
	NoConflict Money	41.17±17.80	Student	11.34	23	6.83E-11	1.38E+08	4.73
		43.38 [28.125 56.81]	Wilcoxon	300		1.94E-05	2264.991	
	NoConflict Shock	-14.25±27.90	Student	-2.50	23	0.02	2.733	-1.04
		-22.13 [-30.75 -2.69]	Wilcoxon	75		0.033	2.699	
**Ambiguous**	Conflict Money	-23.80±19.16	Student	-6.33	25	1.25E-06	14497.856	-2.53
		-25.5 [-38.25 -8.25]	Wilcoxon	17		5.99E-05	6407.894	
	Conflict Shock	-24.78±23.66	Student	-5.34	25	1.55E-05	1447.137	-2.14
		-21.50 [-43.75 -8.63]	Wilcoxon	28.5		1.99E-04	274.725	
	NoConflict Money	32.25±27.92	Student	5.89	25	3.81E-06	5203.687	2.36
		40.75 [15.56 49.63]	Wilcoxon	327		2.27E-05	983.748	
	NoConflict Shock	-26.20±20.92	Student	-6.39	25	1.10E-06	16313.969	-2.56
		-28.00 [-44.06 -17 75]	Wilcoxon	19		7.41E-05	1055.828	

Supplementary Table 4.

Differences in Reported Probabilities. Result of the two-tailed one-sample t-test vs zero on the difference between reported probabilities for the low-shock minus high-shock symbol, separately for the Considerate (green, N=29), Lucrative (blue; N=24) and Ambiguous (orange, N=26) preference group, and for the Conflict and NoConflict condition, as in Figure 3c,d. The first column indicates for which outcome and condition (Conflict, NoConflict) the difference was computed, followed by the average (± Standard Deviation) and median [25% 75%] difference over individuals belonging to each groups, the test performed, its statistical value, the df, p-values and Bayes Factors. Please note that the parametric tests are justified in this case, as the differences were normally distributed. The non-parametric test was added for consistency with the majority of the analyses reported in the main manuscript, which needed a non-parametric testing due to violation of normality. Cohen's d was calculated with the formula: $2t/\sqrt{df}$. Considerate participants in green, Lucrative in blue and Ambiguous in orange. Source data are provided as a Source Data file.

Paired Samples T-Test

	(symbol) − (symbol)	**Test**	**Stat**	**z**	**df**	***p***	**BF_{10}**	**Cohen's *d***
**Considerate**	Conflict Money- Shock	Student	4.08		28	3.37E-04	87.816	1.54
		Wilcoxon	377.5	3.46		5.62E-04	104.789	
	NoConflict Money- Shock	Student	12.16		28	1.04E-12	7.63E+09	4.60
		Wilcoxon	435	4.70		3.73E-09	14156.46	
	Conflict (Money – Shock)- NoConflict (Money- Shock)	Student<br>Wilcoxon	-7.81<br>5	 -4.6	28	1.66E-08<br>4.55E-06	806111.8<br>23750.99	-2.95
**Lucrat ive**	Conflict Money- Shock	Student	-2.82		23	0.01	4.979	-1.18
		Wilcoxon	67	-2.37		0.018	7.97	
	NoConflict Money- Shock	Student	7.70		23	8.14E-08	182100.1	3.21

		Wilcoxon	295	4.14		1.19E-06	10884.46	
	Conflict (Money – Shock)- NoConflict (Money- Shock)	Student	-11.3		23	7.29E-11	1.30E+08	-4.71
		Wilcoxon	1	-4.26		2.38E-07	5497.203	
**Ambiguous**	Conflict Money- Shock	Student	0.33		25	0.745	0.218	0.13
		Wilcoxon	178.5	0.08		0.949	0.212	
	NoConflict Money- Shock	Student	7.29		25	1.22E-07	125484.9	2.92
		Wilcoxon	318	4.18		3.04E-05	82587.56	
	Conflict (Money – Shock)- NoConflict (Money- Shock)	Student	-8.15		25	1.67E-08	801108.4	-3.26
		Wilcoxon	5	-4.33		1.58E-05	1013.15	

Supplementary Table 5. **Comparison in Reported Probabilities.** Result of the two-tailed matched paired *t*-test comparing the differences in Supplementary Table 4 between money and shock (dotted colored lines under the violins in Figure 3c), and between Conflict and NoConflict conditions for money (solid colored lines below the violins in Figure 3c-d). Background colors indicate the group of interest: Considerate (green, N=29), Lucrative (blue, N=24) and Ambiguous (orange, N=26). The first column indicates for which outcome and condition (Conflict, NoConflict) the difference was computed, followed by the average (± Standard Deviation) and median [25% 75%] difference over individuals belonging to each groups, the test performed, its statistical value, the df, *p*-values and Bayes Factors. Please note that the parametric tests are justified in this case, as the differences were normally distributed. The non-parametric test was added for consistency with the majority of the analyses reported in the main manuscript, which needed a non-parametric testing due to violation of normality. Cohen's *d* was calculated with the formula: $2t/\sqrt{df}$. Considerate participants in green, Lucrative in blue and Ambiguous in orange. Source data are provided as a Source Data file.

Supplementary Note 10. Model comparison in ConflictDropout blocks

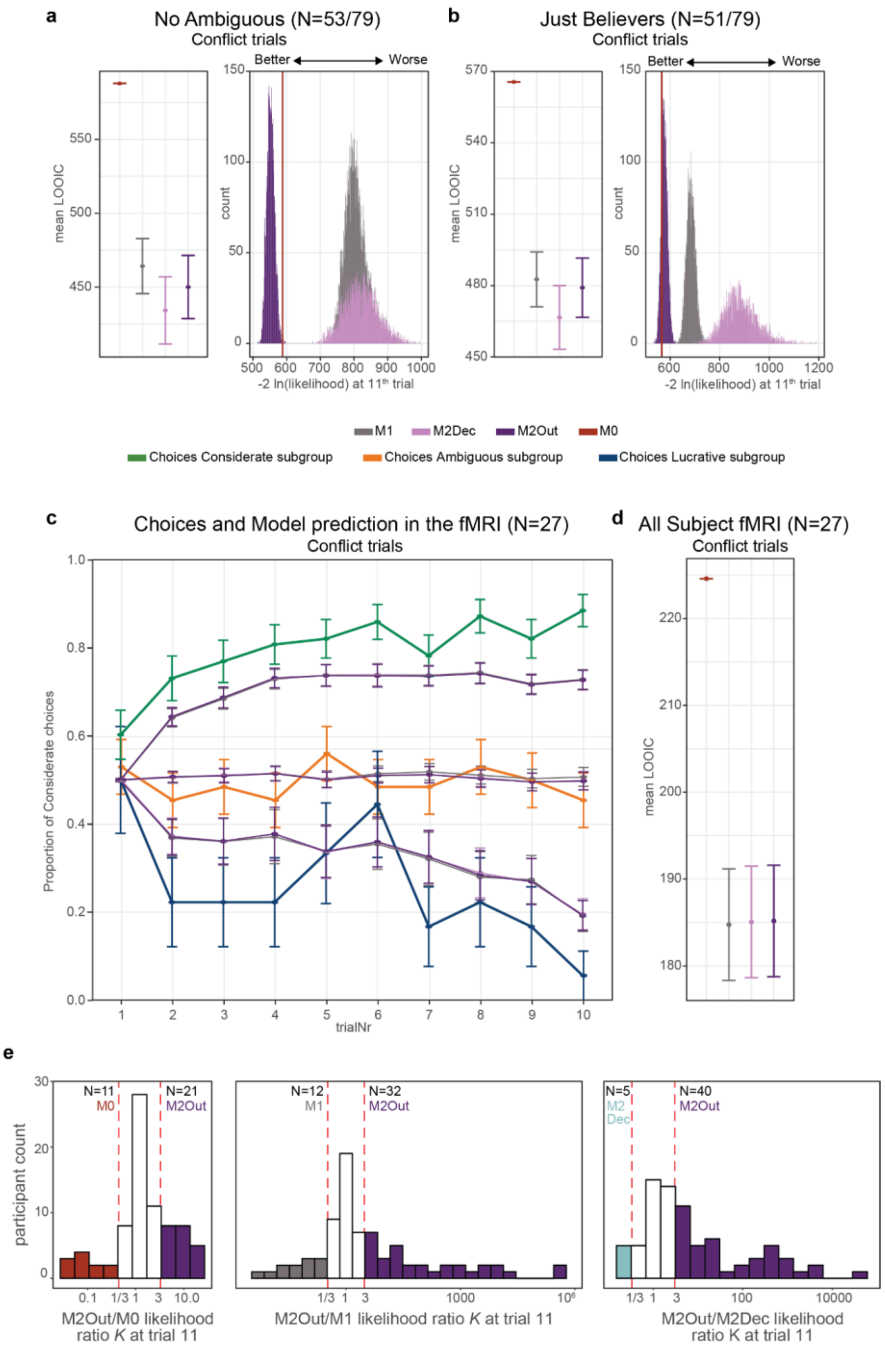

Supplementary Fig. 7. Model Comparison. a Mean LOOIC over trials 1-10 and the likelihood of the choices during devaluation at the 11th trial (as in Figure 5d,f), but excluding participants with ambiguous preferences. Error bars: standard error of the estimated LOOIC. **b** As in b but excluding participants that expressed doubts. **c** Observed (in green, orange and dark blue) and predicted (in gray, light and dark violet) choices over the 10 trials of the Conflict condition for the fMRI data. Error bars: standard error of the mean across participants. **d** Mean LOOIC for all fMRI participants. Error bars: standard error of the estimated LOOIC. **e** The distribution of likelihood ratio (K) across participants in favor of M2Out relative to the competing models. For each Online participant, we summed the log-likelihood of the 11th trials of all ConflictDropout blocks, and calculated K=exp(log_lik_M2Out)/exp(log_lik_OtherModel). Using traditional bounds for log-likelihood ratios used for Bayes factors, K>3 represents subjects with at least modest evidence to have been more likely to use M2Out than the alternative, and K<⅓, more likely to have used the alternative model. Note that participants with K>3 (shown in indigo) always outnumber those for K<⅓, showing that also at the single subject level, M2Out is the model most favored by the data during devaluation. Source data are provided as a Source Data file.

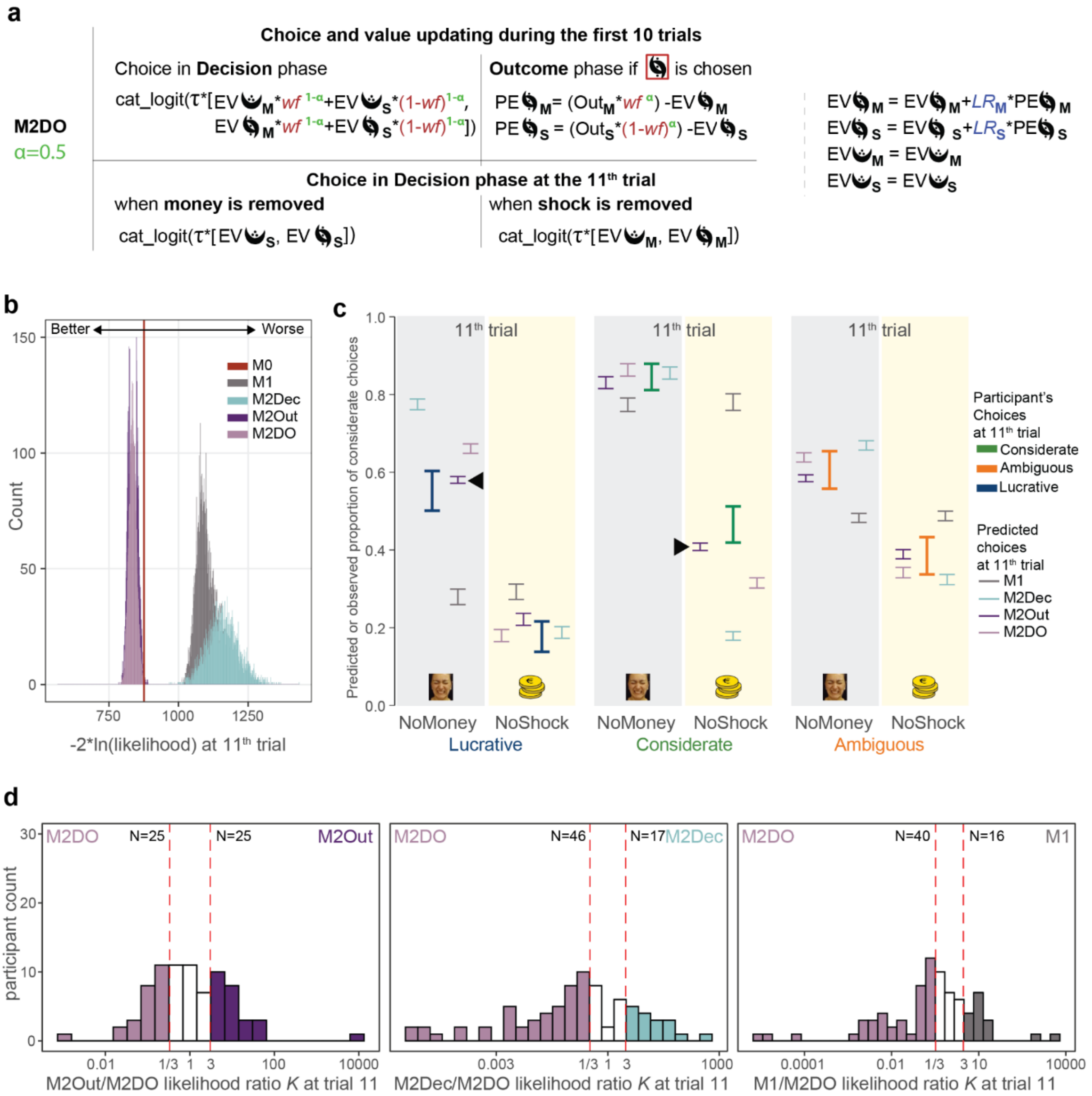

Supplementary Fig. 8. M2 with weighting at both Decision and Outcome (M2DO). a Model formalization for a model that distributes the effect of preference across the decision and outcome phase. Same as in Figure 5, except that an exponent α (shown in green) distributes how much of the weighting occurs during outcome and decision phase. The models M2Out and M2Dec represent special cases of this model with α=1 and α=0, respectively. Using α=0.5 distributes weighting equally across the outcome and decision phase. τ=inverse temperature, *LR*=learning rate, EV=Expected Value, PE=Prediction Error, Out=Outcome. Subscript M=money and S=shocks. Outcomes are coded by value: high-shock Out_S=-1, low-shock Out_S=+1, high-Money Out_M=+1, low-money Out_M=-1. **b** Distribution over 4000 posterior draws of the summed log likelihood of the 11th trial over all participants multiplied by -2 to place values on the information scale as for LOOIC, with lower values indicating better predictions, and zero, perfect predictions. M2DO with α=0.5 and M2Out performed similarly well and outperformed all other models. Note, that in principle, α could be fit to each participants data. However, we know that M2Out and M2Dec make identical behavioral predictions for the first 10 trials, and only start to make distinguishable predictions on the 11th trial, when dropout occurs. Accordingly, the first 10 trials cannot constrain the estimate of α, as α=1 and α=0 make identical prediction, and fitting would thus only depend on 8 trials per participants in our design. We therefore did not attempt such fitting, and simply compared the likelihood of 3 possible values (α=1, i.e. M2Out, α=0.5, i.e. M2DO, and α=0, i.e. M2Dec). M0 in red, M1 in grey, M2Dec in light blue, M2Out in purple and M2DO in lavender. **c** Thicker green, orange and dark-blue lines indicated participant's choices at trial 11. Thinner gray, light magenta, light and dark violet lines indicate model predictions at trial 11. Gray and yellow backgrounds highlight whether money (gray) or shock (yellow) were removed at trial 11. Trial 11 is not included in model fitting. Black arrowheads indicate our M2Out is still closer to actual choices in the most interesting conditions, when a shift in decision is expected: moving toward the considerate option when the money is removed for the Lucrative group, and moving toward the lucrative option when shock is removed for the Considerate group. Error bars: standard error of the mean across participants (N=29 Considerate in green, N=24 Lucrative in blue, N=26 Ambiguous in orange). **d** Same as Supplementary Fig. 7e, but for M2DO with α=0.5. Source data are provided as a Source Data file.

Supplementary Note 11. Age and gender differences across studies

As can be seen from Table 1, there is a significant age difference across the Online and fMRI study (Mann-Whitney t-test, BF_{10}=8.022, p<0.001). Additionally, the fMRI study only includes female participants, while a mixed sample is included in the Online experiment. In order to understand whether such differences are a problem for combining the two datasets, we need to consider the aims of our study. Generally we were mainly interested in understanding (i) whether people that maximize their gains suppress their empathy failing to learn that their choices also harm others, or learn to represent both action-outcome contingencies equally well and nevertheless decide to maximize their own gains; and (ii) how the brain updates values based on shocks to others.

It is therefore important to note, that the premise for our combination of more extensive behavioral testing in the online study with the fMRI study is not that the participants in the two studies have the same distribution of preferences between self-money and other-shocks, but rather, that the same computational model can describe their choices. This is because the fMRI study does not include the Dropout trials that allow us to determine which of the models best describe their learning strategy, and we must thus use a model chosen in the online study to extract the hidden variables used in the fMRI analysis. To justify the combination of the two dataset, it is thus important to determine whether (i) M2Out remains the winning model even when only considering the female sub-sample of our Online study, and (ii) a sample of female participants with an age composition that does not differ significantly from that in the fMRI study would still yield evidence in favor of M2Out outperforming M2Dec and M1.

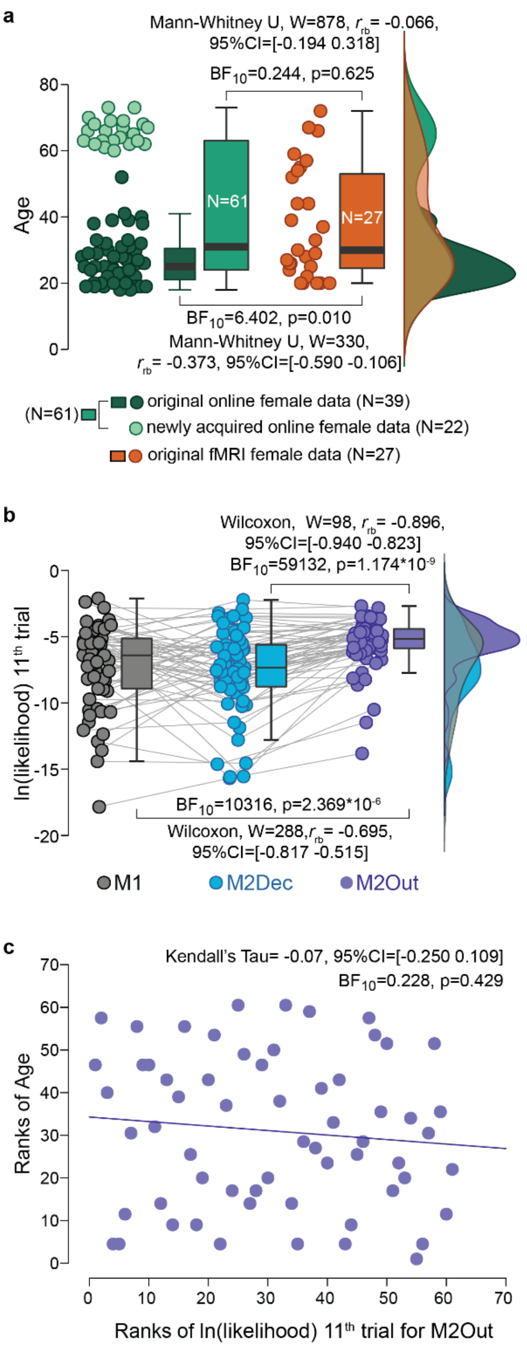

Supplementary Fig. 9. Gender and age differences across studies. a Distribution of participants' age in the different subgroups. Lightest green represents the newly acquired online older female participants (N=22); darkest green the online female group presented in the main text (N=39), and intermediate shade of green, the whole sample (N=61). Box-plot show the median and quartiles, with whiskers showing the range of datapoints between Q1-1.5IQR and Q3+1.5IQR. **b** Log-Likelihood at trial 11 estimated for the three models of interests. N=61. Box-plot show the median and quartiles, with whiskers showing the range of datapoints between Q1-1.5IQR and Q3+1.5IQR. M1 IN grey, M2Dec in light blue and M2Out in purple. **c** Correlation between age (full Online female, N=61) and Log-Likelihood at trial 11 for M2Out. Statistical significance is reported in each panel for the relevant non-parametric test (independent sample t-test in a, paired t-test and b and correlation in c), and for both the frequentist and Bayesian approach. BF>3 = evidence in favor of a difference, BF<⅓= evidence in favor of a lack of difference. All tests are two-tailed. Source data are provided as a Source Data file.

Given that our initial online sample differed in age from the fMRI sample, during the manuscript revision we collected an additional online sample of women (N=22; lighter green dots in Supplementary Fig. 9a), so that by combining them with the females from the online study originally acquired, we now have a group of online female participants that no longer differs in age from that in

the fMRI sample (Mann-Whitney *t*-test, BF_{10}=0.244, p<0.625; Supplementary Fig. 9a). We then compared the log likelihood of the 11th trial for the three models in this combined online sample that is gender and age-matched, and found evidence that M2Out still outperforms M2Dec and M1 (Supplementary Fig. 9b). M2Out outperforms the other models, even when only considering the online females originally collected (N_{female}=39; M2Out vs M1: Wilcoxon W=123, BF_{10}=269.565, p=9.093*10-5; M2Out vs M2Dec: Wilcoxon W=65, BF_{10}=976.468, p=6.247*10-7). We finally ran a correlation between the log likelihood of the 11th trial estimated with M2Out and age including all online females (N=61), and found evidence in favor of a lack of correlation (Supplementary Fig. 9c). From these control analyses we can therefore conclude that using M2Out to estimate the learning parameters in the fMRI study is justified.

Supplementary Note 12. Ambiguous group characterization

To gain deeper insights into the behavior of the ambiguous participants, we ran some explorative additional analyses to address the following questions.

1. **Do Ambiguous individuals alternate their preference across blocks?**

To understand whether individuals of the Ambiguous group choose to alternate their preference between blocks, we first plotted the average of the proportion of pain-reducing choices per block for each participant and all the blocks of the Ambiguous group. Only the first 10T are included, and we combined both the Dropout and NoDropout blocks (total number of blocks = 12: 4 ConflictNoDropout, 4 ConflictShockDropout, 4 ConflictMoneyDropout). From this graph it is hard to see whether participants choose totally randomly (therefore staying around 0.5 for each block), or whether they use a strategy in which within a block they have a preference, but voluntarily switch preference from block to block (going from a clearly considerate choice for one block to a clearly lucrative choice in another block, i.e. being above chance considerate, or lucrative, but only within blocks). The graph includes some participants that have relatively extreme scores in certain blocks, suggesting that some participants may have had per-block-preferences. The problem is that doing a binomial per block with only 10 trials to see if there was a significant preference per block lacks sensitivity - one needs 9/10 choices in one direction to have a significant binomial, and that can hardly be expected given that participants need to learn within a block.

To investigate whether ambiguous participants display significant preference within certain blocks, but may switch preference between blocks, we therefore reasoned as follows. If participants have a preference in a block, the probability to choose the considerate option should deviate from 0.5. If they switch their preference between blocks, sometimes the deviation might be upwards (when expressing a considerate preference) and sometimes downwards (when expressing a lucrative preference). Hence, evidence of - albeit alternating - preference would be captured by the sum of the squared deviations from 0.5 (SSD) across the 12 blocks. To assess how surprising a given summed square deviation would be, we can compare it against simulated choices of a random chooser (possibly as a consequence of not having learned the task). We therefore estimated this SSD across blocks for each participant, and ran a simulation to estimate how a distribution of random choices would look like. We then estimated the critical value (i.e. 95th or 99th percentile of the null distribution SSD) and compared our subjects to it. Three (blue lines in Supplementary Fig. 10) out of 26 participants show SSD values in excess of the 99th percentile of the null distribution (Supplementary Fig. 11). This suggests that there is evidence that in the Ambiguous group at least 3 out of 26 ambiguous participants do not choose randomly, but do show a preference in some of the blocks, albeit with different preferences across different blocks. Note that 3/26 at alpha=0.01 is larger than what we would expect based on a 1% false positive rate alone (binomial, p=8.8E-5). However, for the vast majority of the ambiguous group (23/26=88%) we have no evidence that their choices are non-random at alpha=0.01, and for 20/26 (77%), that they are non-random at alpha=0.05.

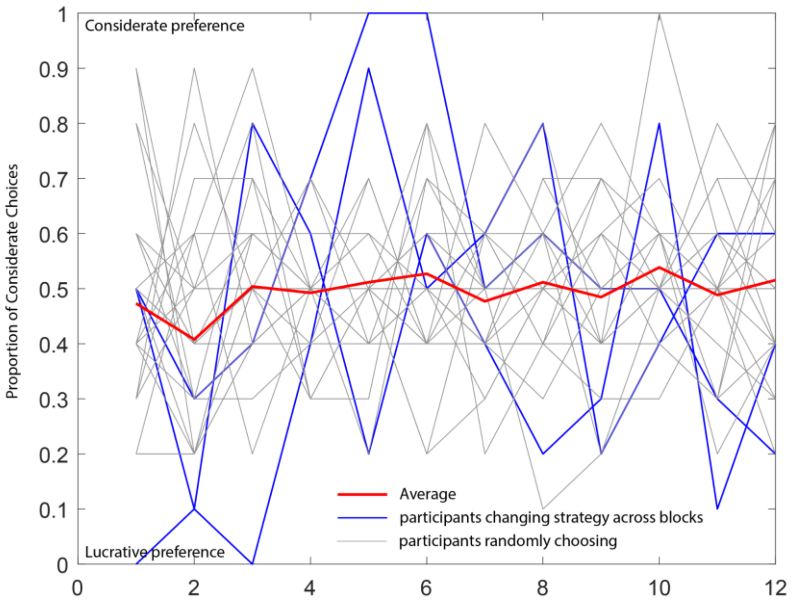

Supplementary Fig. 10. Choices over blocks. Gray and blue lines (one for each ambiguous participant of the Online dataset) indicate the average choice over the 10 trials of ConflictNoDropout and ConflictDropout for each of the 12 blocks. Blue highlighted participants are those that show evidence of having significant, albeit changing, preference over blocks, as evidenced by an SSD above the 99th percentile of the null distribution SSD as shown in Supplementary Fig. 11. Red represents the overall average across participants. Source data are provided as a Source Data file.

2. Do Ambiguous individuals lack a clear preference (i.e. choose more randomly)?

We also examined whether the group of Ambiguous participants overall shows higher SSD than would be expected by chance, by summing the SSD of the 26 participants, and comparing it against a null distribution obtained via 10000 simulations of a group of 26 random choosers, and found the real group SSD to be in the 98.1th percentile of the null distribution of group SSD, showing that as a group, the ambiguous participants also showed more extreme choices than expected if they were not to have any preference at all.

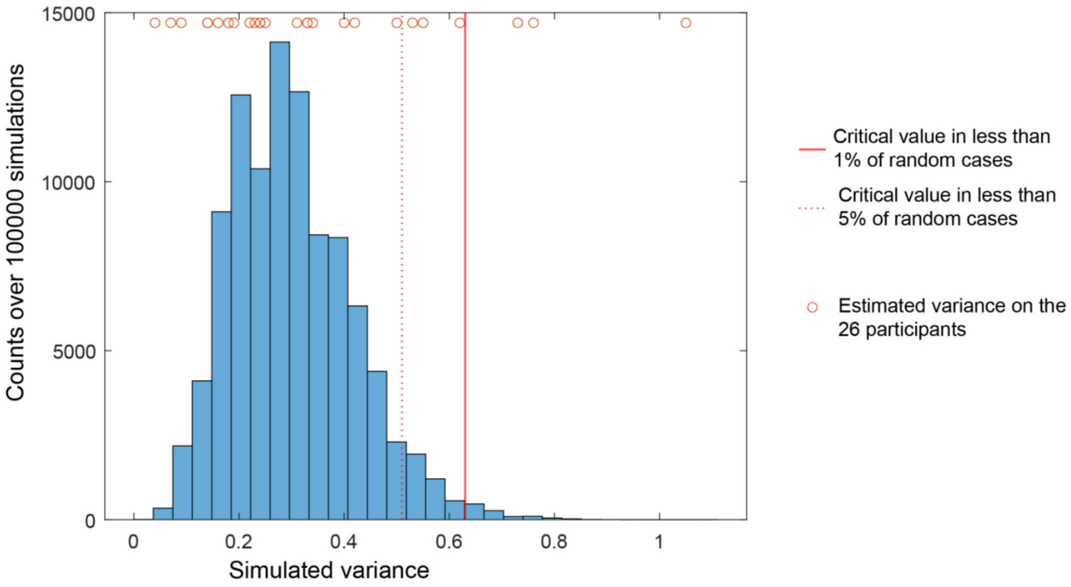

Supplementary Fig. 11. Real vs. Simulated Sum of Squared Deviation from 0.5. We simulated 100000 random choosers performing 10 trials per block for 12 blocks. For each block and simulated participant, we calculated the proportion of considerate choices, and then, for each participant a sum of the squared deviations (SSD) by summing the squared deviation from 0.5 across the 12 blocks. The blue histogram shows the distribution of the SSD across these 100000 simulated random choosers. The SSD of the 26 ambiguous participants is shown as open orange circles above the histogram. The solid and dotted vertical lines represent the 99th and 95th percentile of the simulated random choosers. Real participants with values above those lines thus are unlikely to have chosen entirely randomly, as their preference per block fluctuated more than random choices would predict.

3. **Do Ambiguous individuals have poorer implicit learning?**

During the Conflict condition, because of the presence of a conflictual decision, participants' flatter learning curves could be explained by a poorer learning, but also by other factors, such indecision or different strategies in performing the task. Looking at the NoConflict condition is therefore the most appropriate test to investigate whether the Ambiguous group learns similarly to the other groups, as it represents the win-win situation in which participants can learn to increase their chances to add a financial bonus without causing any pain to the confederate. Importantly, in the NoConflict condition, whether participants value reducing shocks to others and/or increasing their own financial gains, one option is clearly better than the other, and the percentage favorable choices can be interpreted as a measure of performance. Along that reasoning, when looking at Ambiguous participants' choices, we can clearly see that on average their choices remain worse than the other groups also in the NoConflict conditions (Supplementary Fig. 5a,b and c). Even at the 10th trial - i.e. at the moment in which participants should have learned the symbol-outcome association for each block - the Ambiguous group remains the lowest in % favorable choices (main effect of group Kruskal-Wallis ANOVA=10.924, p=0.004; Mann-Whitney U independent sample t-tests: $W_{\text{Ambiguous vs Lucrative}}$=197, p=0.023, BF_{10}=1.516; $W_{\text{Ambiguous vs Considerate}}$ =192, p=0.001, BF_{10}=8.235) confirming that overall the Ambiguous group learns more slowly than the other groups. Interestingly, this 10th trial is also the one after which participant's explicit reports are collected in the NoDropout blocks.

We then, as suggested by the reviewer, looked at individual parameters derived from our learning model, *LR* and Tau in particular. We ran two one-way non-parametric ANOVAs, one on the *LR* for money and one on the *LR* for shock, with group (Ambiguous, Considerate and Lucrative) as between-subjects factor. The Kruskal-Wallis test indicates a significant effect of group for both the *LR* for money (H=18.842, p=8.099*10^$^{-5}$) and shock (H=6.049, p=0.049) in the NoConflict condition. Post-hoc non-parametric frequentists and Bayesian t-tests show that the *LR* for shock differs from that of the Considerate group, and the *LR* for money differs from that of the Lucrative group. The figure below illustrates the result and indicates the statistical values of this analysis (Supplementary Fig. 12a,b) and the same analysis performed on the Conflict condition (Supplementary Fig. 12d,e). Overall, these results suggest that despite the *LR* of the Ambiguous group remaining within the range of the other two groups - therefore supporting that some learning occurs - their average is lower than that of the groups that values a particular outcome (as revealed by their choices under Conflict). That is to say, for Money, the Lucrative participants are known to value maximizing Money, and their *LR* for Money is significantly higher than that of the Ambiguous participants. For Shocks, a similar trend occurs: the considerate participants value avoiding shocks, and their *LR* for Shocks is higher than that of the Ambiguous participants. On the other hand, the *LR* of the Ambiguous group resembles that of the groups that seem to value that outcome little, and hence the Ambiguous *LR* for shocks resembles that of the Lucrative participants that do not particularly value shocks, and their *LR* for money resembles that of the Considerate participants that do not particularly value money. A limitation of this analysis is the fact that *LR* is less accurately estimated for the non-preferred outcome. As Ambiguous participants show a less clear preference, their *LR* may also be less accurately estimated.

The non-parametric ANOVA on the parameter Tau, also indicates a significant main effect of group (Kruskal-Wallis main effect of group, H=17.105, p=1.93*10^{-4}). Non-parametric post-hoc t-tests indicate the parameter Tau is significantly the lowest for the Ambiguous group compared to the others (Supplementary Fig. 12c), suggesting the Ambiguous group makes more random decisions than the other groups even when the model suggests that they have learned a similar difference in expected values across the alternative options. The exact same patterns of results was observed for the Conflict condition (Supplementary Fig. 12f).

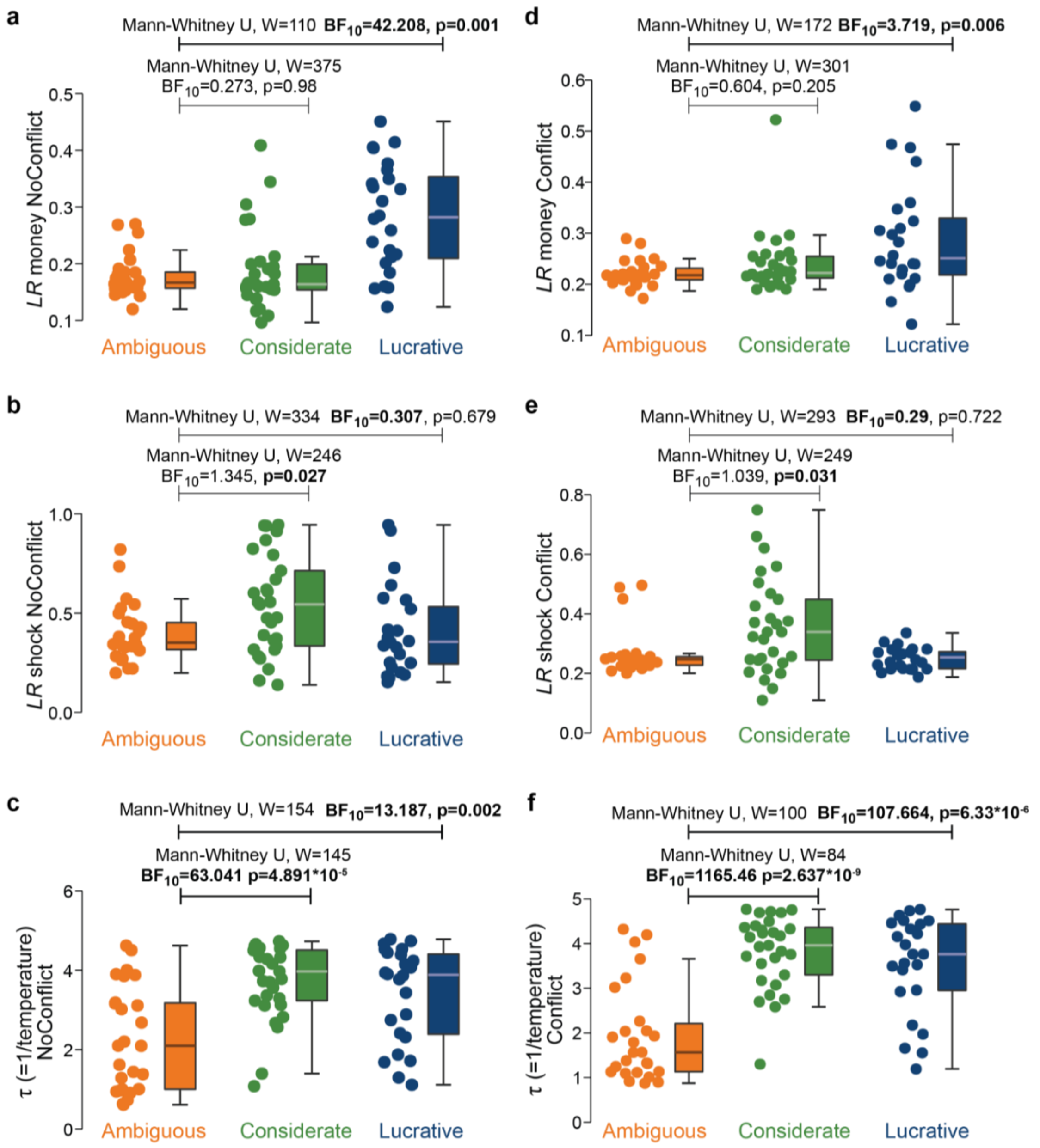

Supplementary Fig. 12. Learning parameters across groups. a-c LR_M, LR_S and τ values for the Ambiguous (orange, N=26), Considerate (green, N=29) and Lucrative (dark blue, N=24) online participants, estimated by M2Out for the first 10 trials of the NoConflict condition. Note that these NoConflict trials were not included in the model fits presented elsewhere, which only include the Conflict trials. **d-f** same as in a-c but estimated for the Conflict condition. Statistical significance is reported in each panel for the non-parametric independent samples planned two-tail post-hoc testing, both for the frequentist and Bayesian approach. BF>3 = evidence in favor of a difference, BF<⅓= evidence in favor of a lack of difference. All box-plot show the median and quartiles, with whiskers showing the range of datapoints between Q1-1.5IQR and Q3+1.5IQR. Source data are provided as a Source Data file. Source data are provided as a Source Data file.

Finally, we looked at whether Ambiguous participants were overall slower in making their choices in terms of reaction times (Supplementary Fig. 13). The Kruskal-Wallis ANOVA shows a main effect of group (8.166, p=0.017). Non-parametric independent samples t-tests on reaction time (RT) data from the NoConflict condition, indicate a trend for the Ambiguous participants to be slower than the Considerate group, but there is evidence for a lack of a difference in RT with the Lucrative group. The same ANOVA on the RT during the Conflict condition does not reveal a main effect of group (4.657, p=0.097). Overall, the evidence suggesting the Ambiguous group takes longer to choose is very small, and the RT analysis supports more the idea that the Ambiguous responds with a comparable RT than the other groups.

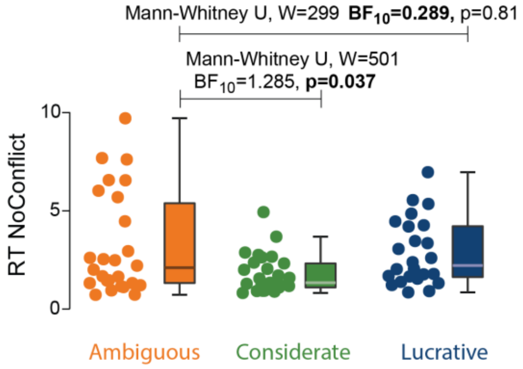

Supplementary Fig. 13. Reaction time across groups. RT values for the Ambiguous (orange, N=26), Considerate (green, N=29) and Lucrative (dark blue, N=24) online participants, for the first 10 trials of the NoConflict condition. Box-plot show the median and quartiles, with whiskers showing the range of datapoints between Q1-1.5IQR and Q3+1.5IQR. Statistical significance is reported in each panel for the non-parametric independent samples planned post-hoc two-tail testing, both for the frequentist and Bayesian approach. BF>3 = evidence in favor of a difference, BF<⅓= evidence in favor of a lack of difference. Source data are provided as a Source Data file.

4. Do Ambiguous individuals have poorer explicit learning?

When looking at the graphs of Figure 3c,d, even Ambiguous participants report the association probabilities with above-chance accuracy. However, it is also noticeable that the Ambiguous group's accuracy in recalling the difference between the two symbols for a particular outcome seems closer to the accuracy of the group that values this particular outcome less: they report this difference for money less accurately than the Lucrative group and only as well as the considerate group, and they report this difference for shocks less accurately than the Considerate participants and only as well as the Lucrative participants. In other words, while the Considerate groups tends to recall more accurately the difference between the two symbols when reporting the probabilities of shock, and the Lucrative recall better the difference when reporting the probabilities of money, the Ambiguous group accuracy is comparable to the probabilities the Considerate group reports for the money and the Lucrative for the shock.

Supplementary Fig. 14 illustrates this effect more clearly and reports the statistical values of the non-parametric independent t-tests we ran. While in the NoConflict condition the accuracy in recalling the probabilities of the two symbols associated with money seems not to clearly differ across group (Kruskal-Wallis ANOVA main effect of group=1.146, p=0.284), a difference in accuracy becomes visible when recalling the probabilities associated with shock (Kruskal-Wallis ANOVA main effect of group=10.371, p=0.001). In particular, the Ambiguous group recalls the probabilities of the symbol-shock association worse than the Considerate group. When repeating the same analyses on the Conflict condition, this effect accentuates and the accuracy of the Ambiguous group worsens also for the symbol-money associations, for which the Ambiguous becomes worse than the Lucrative group.

These analyses, again support the idea that although the Ambiguous group explicitly learns *something* about the difference between the two symbols, their accuracy tends to be lower than the accuracy of the Considerate and Lucrative groups for their outcome of value. If the choices of Ambiguous participants were to have been the result of excellent learning combined with a strategy to balance the two outcomes, one may have expected them to recall both outcomes as well as the group prioritizing that outcome, rather than as poorly as the group not prioritizing that outcome in their decisions.

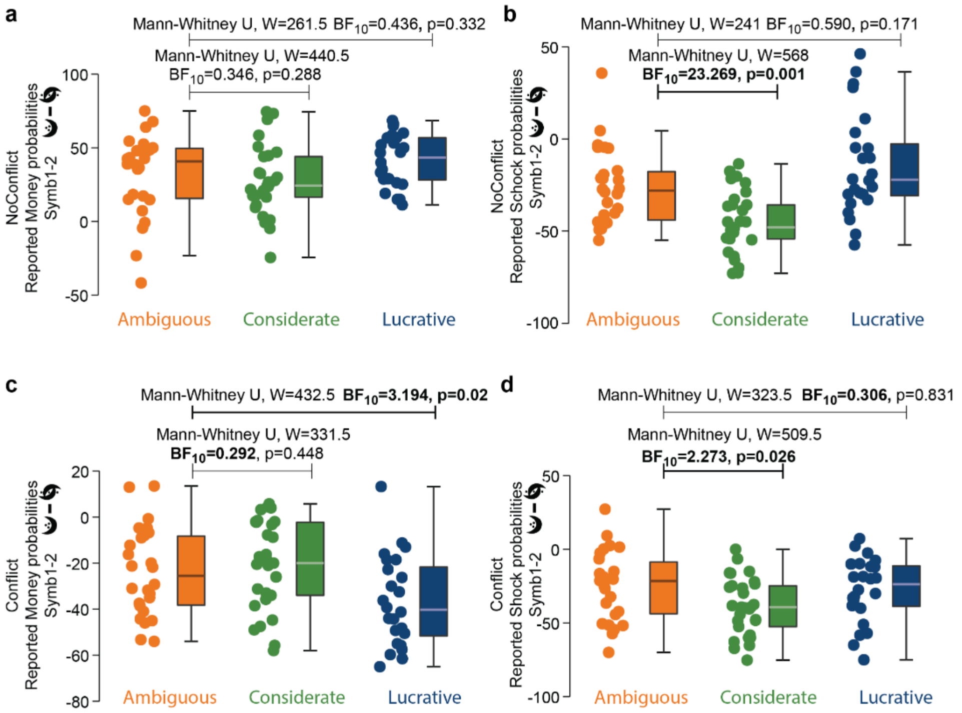

Supplementary Fig. 14. Explicit recall comparison across groups. a-b Difference between Money a and Schock b reported probabilities for the low-shock minus high-shock symbol (Symbol1-2), separately for the Considerate (green, N=29), Lucrative (blue, N=24) and Ambiguous (orange, N=26) preference group, for the NoConflict condition (same as in Figure 3d). **c-d** same as in a-b (and Figure 3c) but for the Conflict Condition. Statistical significance is reported in each panel for the non-parametric independent samples planned two-tail post-hoc testing, both for the frequentist and Bayesian approach. BF>3 = evidence in favor of a difference, BF<⅓= evidence in favor of a lack of difference. Box-plot show the median and quartiles, with whiskers showing the range of datapoints between Q1-1.5IQR and Q3+1.5IQR. Source data are provided as a Source Data file

To summarize, evidence that the ambiguous group does learn comes from:

- The fact that the in the explicit report the ambiguous group reports differences between the probabilities associated with the two symbol that are significantly different from zero, both for the Conflict and NoConflict condition and for both the money and shock probabilities (Figure 3a,c)
- The fact that choices in the NoConflict conditions are above chance level, and in the same directions of the other groups
- The fact that at the 11th trial of the Dropout blocks participants significantly discriminate between MoneyDropout and ShockDropout blocks (i.e. Figure 4a, 11th trial)
- The fact that on average their choices are more extreme than expected by chance (Supplementary Fig. 11)
- Reaction time mostly remains within the range of the other two groups (Supplementary Note §13).

Evidence that the ambiguous learn more slowly than the other groups comes from:

- The proportion of favorable choices is on average lower than in the other groups in the NoConflict condition (Supplementary Fig. 6b). Even at the 10th trial - i.e. at the moment in which participants should have learned the symbol-outcome association for each block - the Ambiguous group remains the lowest in % favorable choices, suggesting that overall the Ambiguous group learns more slowly than the other groups.
- For ambiguous participants the *LR* for money is lower than the Lucrative group, and the *LR* for shock lower than the Considerate group (Supplementary Fig. 12a,b).
- Tau indicates that the Ambiguous group performs more random decisions compared to the other groups (Supplementary Fig. 12c).
- Accuracy in explicit recall remains lower for the Ambiguous group compared to the preferred outcomes of the other groups (Supplementary Fig. 14a,b).

Supplementary Note 13. M2Out Parameter Distributions and Recovery

The distribution of the parameters that were estimated for $\boldsymbol{\tau}$, LR_S and $\boldsymbol{\tau}$, LR_M can be found in Supplementary Fig. 15a,b. We also performed a parameter recovery assessment on the M2Out model to assess the robustness of inferences on the parameter values estimated from this model. Generating values for the parameters *wf*, LR_S and LR_M were all drawn from a uniform distribution between 0 and 1, and $\boldsymbol{\tau}$ from an exponential distribution between 0.2 and 5 (i.e., the natural logarithm of $\boldsymbol{\tau}$ was drawn from a uniform distribution between -1.61 and 1.61). Parameter sets were sampled using a Latin hypercube sampling design[53,54] to ensure an adequate coverage of the joint parameter space. The simulated data were generated using the full process of the M2Out model (i.e., simulated trial 1 informs simulated trial 2, etc.), with the amount of data matching the fMRI experiment (i.e., 6 blocks, each with 10 trials). Parameter values were estimated using Differential Evolution Markov Chain Monte Carlo[55], with 12 chains run in parallel for 2000 iterations and the first 1000 iterations discarded as burn-in. The priors on the *wf*, LR_S and LR_M parameters were all truncated normal distribution (between 0 and 1) with mean 0.5 and standard deviation 0.2, while the prior for the $\boldsymbol{\tau}$ parameter was a truncated normal distribution (between 0 and infinity) with mean 1 and standard deviation 3. The estimates plotted in Figure 6c display the estimated posterior means from each simulated data set.

In the main manuscript, we use a hierarchical Bayesian model to estimate these parameters under the assumption that we sampled multiple individuals from the same underlying population. In particular for LR_S and LR_M, it is likely that different participants may gravitate onto similar LR_S and LR_M given the similarity in volatility experienced by them. For the parameter recovery we perform here, this assumption is not true: we deliberately sampled the entire space of possible LR_S and LR_M using a uniform distribution. Accordingly, for the parameter recovery, each simulated behavior was analyzed individually using a non-hierarchical model. The priors for this individual implementation were not informed by the hyper-parameters of the final study, and were simply aimed at informing us about the proportion of all possible variance in these parameters that can be retrieved from analyzing participants one at a time.

The inverse temperature ($\boldsymbol{\tau}$) parameter had a relatively broad distribution across our participants in our two experiments (Supplementary Fig. 6A), and parameter recovery shows that when simulating participants with different $\boldsymbol{\tau}$, the $\boldsymbol{\tau}$ estimates recovered by fitting M2Out correlated quite highly with the simulated $\boldsymbol{\tau}$ values (Kendall's Tau ($\boldsymbol{\tau}_{simulated}$, $\boldsymbol{\tau}_{estimate}$)=0.53, p<0.001, BF_{10}>1000). As reported in the main text, the same was true for *wf*. In contrast, parameter recovery shows that although the learning rates can be significantly recovered by M2Out, t,he correlation values are more modest than for *wf* and τ (Kendall's Tau ($LR_{Ssimulated}$, $LR_{Sestimate}$)=0.25, Kendall's Tau ($LR_{Msimulated}$, $LR_{Mestimate}$)=0.24, both p<0.001, BF_{10}>1000), and the $\boldsymbol{\tau}$ values obtained from our model-fitting should thus be interpreted more tentatively.

In particular, we find that $\boldsymbol{\tau}$ is difficult to estimate for the outcome that participants consider less in their choices: for simulated participants with *wf*<0.1 (i.e. that minimize shocks to others), Kendall's Tau between simulated and estimated values drops to 0.03 for LR_M but is 0.36 for LR_S, while for simulated participants with *wf*>0.9 (i.e. that maximize gains to the self), it is 0.32 for LR_M but drops to 0.06 for LR_S. Accordingly, we did not include these parameter estimates in the main manuscript due to their reduced robustness.

It might also be noted, that the median learning rates estimated by the hierarchical Bayesian model for the Online experiment (Supplementary Fig. 6B) was close to 0.25, and was very different from the medial of the parameters recovered by the non-hierarchical model with a prior with mean 0.5. Adjusting the prior of the parameter recovery to match the hyper-parameter obtained in the Online experiment fixes this anomaly.

Supplementary Fig. 15.

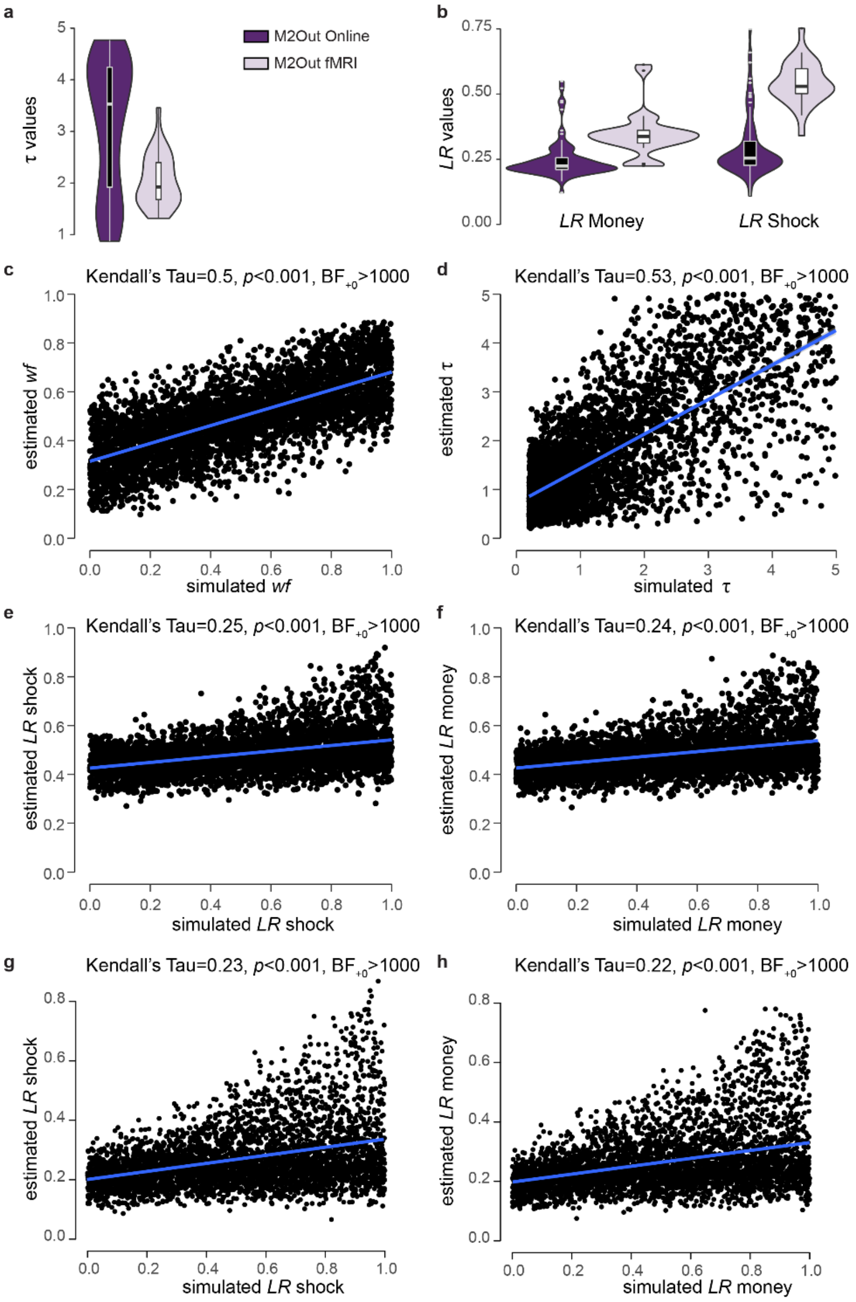

a,b Distribution of parameter estimates for M2Out in the Online (purple, only including the first 10 trials of the ConflictDropout blocks, N=79) or fMRI (lavender, including all trials, N=27) across participants. Violin plots represent the value distribution, the box-plot within, the median and quartiles, the whiskers, the range of datapoints between Q1-1.5IQR and Q3+1.5IQR. **c-f** Parameter Recovery Performance of a non-hierarchical implementation of M2Out. The figure shows the result of simulating the choices of 4000 participants that use M2Out for their decision, with a range of *wf*, τ, LR_S and LR_M values, then estimating the parameters using M2Out. Note that this was done non-hierarchically to avoid influence from one simulation on the other, using priors that were not informed by the findings of our actual study. We performed a Shapiro-Wilk test for bivariate normality that considered for each of the four parameters both the simulated and estimated values. For *wf*, p=0.048, and we thus report both Pearson's r and Kendall's τ. For the other three parameters, the Shapiro-Wilk p was always below 0.001, and we thus only report Kendall's τ. The p and BF values always refer to one-tailed tests with H1: $\tau > 0$, as this test does not require bi-variate normality. Note how for *wf* and τ, the estimated values capture the simulated values fairly well, although the slope of the regression line is <1. For τ, however, the prior distribution (truncated normal distribution, between 0 and 1, with mean 0.5 and standard deviation 0.2) appears to have a stronger influence on the estimated values than the simulated data, which strongly compresses the estimated values towards the peak of the prior and reduces the coupling between simulated and estimated values. **g-h** Adapting the priors for LR_S and LR_M to 0.2, closer to the values observed in the Online study, shifts the recovered parameters to a range closer to what was observed in the online study. Source data are provided as a Source Data file.

To investigate whether the current design and modeling approach can differentiate the learning rate and the weighting factor we run non-parametric correlations between the two parameters, separately for money and shock. Overall, results indicate a lack of significant correlations between *LR* and *wf*. BF additionally indicates clear evidence of a lack of correlation between LR_S and *wf*, and a similar trend for LR_M and *wf* (Supplementary Fig. xx). We can therefore conclude that there is sufficient unique variance for the model to differentiate between *LR* and *wf*.

What we do observe in our data and in simulations, is that, somewhat unsurprisingly, the *LR* parameter estimates in our Bayesian framework are more influenced by the data for the quantity that is more valued: participants with very low *wf*, that mainly chooses to reduce shocks, have estimates for *LR* for shock that are

more constrained by the data while participants with very high *wf*, that mainly choose to maximize gains, have estimates for *LR* for money that are more constrained by the data.

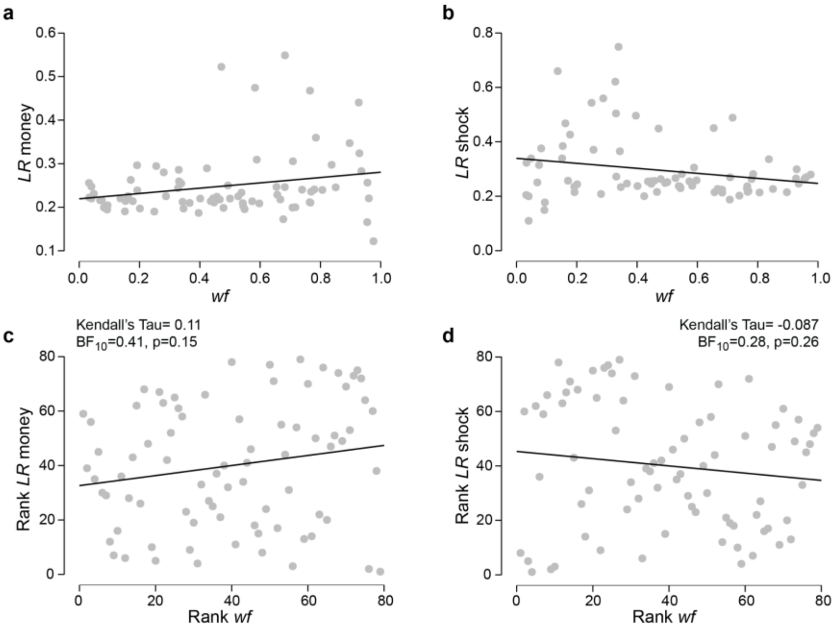

Supplementary Fig. 16: Correlations between *LR* and *wf*. **a** Online participants' (N=79) learning rate values for money (*LR* money) plotted against participant's *wf*. **b** Same as in (a) but for the learning rate for shock (*LR* shock). **c.** Correlation between learning rate for money (*LR* money) and *wf*. **c** As *LR* and *wf* were not normally distributed (Saphiro-Wilk $p<0.01$), the graph plots the rank *LR* money against *wf* to directly reflect the fact that Kendall's Tau is calculated on ranks, rather than actual values as shown in (a). **d** Same as in (c) but for the *LR* shock against *wf*. All tests were two-tail. Source data are provided as a Source Data file.

Supplementary Note 14. Predicting helping based on *wf* but not IRI or MAS

		Bayesian					Frequentist		Kendall's Tau with *wf*
	**Coefficient**	**P(incl)**	**P(incl|data)**	**BFincl**	**Mean**	**SD**	***t***	***p***	
	Intercept	1	1	1	2.643	0.197	2.069	0.058	
	wf	0.5	0.922	**11.74**	-1.669	0.732	-3.009	**0.009**	
**IRI**	Fantasizing	0.5	0.277	0.383	-0.006	0.031	-0.433	0.671	-0.070
	Perspective Taking	0.5	0.261	0.354	-0.003	0.027	-0.575	0.574	-0.249
	Empathic Concern	0.5	0.284	0.396	0.008	0.029	0.899	0.384	-0.153
	Personal Distress	0.5	0.262	0.356	-0.002	0.024	-0.190	0.852	0.053
**MAS**	Power-Prestige	0.5	0.269	0.367	-0.001	0.025	0.170	0.868	0.019
	Retention-time	0.5	0.282	0.393	-0.007	0.029	-1.042	0.315	0.120
	Distrust	0.5	0.405	0.682	0.024	0.039	1.820	0.090	0.109
	Anxiety	0.5	0.311	0.451	-0.014	0.036	-1.137	0.274	0.243

Supplementary Table 6. Bayesian linear regression posterior summary of coefficients.
The table summarizes the Bayesian linear regression model comparison for models explaining the donation in the Helping task using the *wf* of the Learning task (estimated by fitting M2Out on the first 10 trials of the Conflict conditions), the subscales of the IRI [56] (FS, PT, EC and PD) and MAS [46] (Power-Prestige, Retention-time, Distrust and Anxiety). The first column indicates the variable under consideration, followed by the prior probability of inclusion (P(incl)), the posterior probability of inclusion (P(incl|data)), the BF_{incl} indicating how much more likely models including a variable are compared to the average of those not including this variable. $BF_{incl}>3$ is considered moderate, and $BF_{incl}>10$ strong, evidence that a variable explains donation. $BF_{incl}<⅓$ indicates moderate evidence against a variable explaining donation. While *wf* shows a $BF_{incl}>11$, all other variables have a $BF_{incl}<0.7$. The most likely model given the data is therefore the one including the intercept and the *wf* alone [28]. Mean and SD represent the estimates of the weight of the parameter in the regression. The negative weight for *wf* indicates that people with higher *wf* (i.e. with more lucrative preferences) donate less to help others. A frequentist analysis (with its relevant *t* and *p* value) reaches the same conclusions, with $p=0.009$ for *wf* but all other $p>0.05$. Significant *p* values and BF>3 are highlighted in bold and green. The final column indicates the correlation between the psychological tests and the *wf*. None of these correlations were significant (all $p>0.05$). Source data are provided as a Source Data file.

Supplementary Note 15. How to generate fMRI parameter estimates that can be easily interpreted.

In our winning model, M2Out, the magnitude of prediction errors and expected values depends on a participant's *wf* value. Let us consider two participants, A and B, with wf_A=0.1 and wf_B=0.9. Now let us focus on a hypothetical first trial in which both try a symbol and witness a high-money high-shock outcome. Because it is their first trial, their expected values were still set at 0, EV_S=EVM=0, and because it is a high-shock, high-money outcome, OutS=-1 (high-shock) and OutM=+1 (high-money). Their prediction errors, according to M2Out will be different, despite starting from the same EV and witnessing the same outcome, due to their difference in *wf*. Let us focus on the shocks, where M2Out specifies that PES=OutS*(1-*wf*)-EVS. For participant A: PES_A=-1*(1-0.1)-0=-0.9; for B, PES_B=-1*(1-0.9)-0=-0.1. Now let us also consider two hypothetical BOLD responses. Response pattern 1 (BOLD1), assumes that in this voxel witnessing the same shock intensity triggers a similar BOLD response across all participants, independently of *wf*, so that $BOLD1_A$=$BOLD1_B$=1. In contrast, response pattern 2 (BOLD2), assumes that in this different voxel, participants that care more about shocks (like participant A) have a stronger response to witnessing the high-shock than participants (like participant B) that care less about shocks, with a magnitude that linearly depends on *wf*, e.g. $BOLD2_A$=0.9, $BOLD2_B$=0.1. The core question for our fMRI analysis is now to build a design matrix at the first level of the fMRI analysis that yield parameter estimates for PES that can be easily interpreted across participants to identify voxels in which response magnitude does or does not depend on personal preferences. If we directly enter the PES values from M2Out in our fMRI model (after mean-subtraction but without dividing by standard deviation), the parameter estimate *b* for the PES predictor for each participant in our one-trial example would simply be BOLD/PES. In the case of BOLD1, where the BOLD response is the same across participants, $b1_A$=1/-0.9=-1.11, and $b1_B$=1/-0.1=-10. Hence, despite the same BOLD response across participants, the parameter estimates are very different across participants. To use the parameter estimates as a measure of individual differences, this is not desirable. How can we avoid that effect? If we divide PES by (1-*wf*), to reverse the effect of (1-*wf*) in the formula to calculate PES, and we use PES/(1-*wf*) as the predictor, this issue is remedied — PES_A/(1-*wf*)=-0.9/0.9=-1, PES_B/(1-*wf*)=-0.1/0.1=-1 — and the parameter estimates now reflect the equality of BOLD response magnitude as b1A=1/-1=-1, and b1B=1/-1=-1. How do the two methods compare in case BOLD2, where the response did depend on personal preference? When using the actual PES values from M2Out as predictors, $b2_A$=0.9/-0.9=1 and $b2_B$=0.1/-0.1=1. So here, BOLD response magnitude actually differed across the participants, but the parameter estimates do not ($b2_A$=$b2_B$). Again, this is not desirable. If using PES/(1-*wf*), $b2_A$=0.9/-1=-0.9, and $b2_B$=0.1/-1=-0.1. Here, by looking at the parameter estimates, we can directly observe that the responses of A were stronger than those of B for the same outcome. Hence, dividing the PES by (1-*wf*), ensures that participants with similar response magnitudes in the brain get similar parameter estimates, and participants with different response magnitudes in the brain have different parameter estimates.

In reality, our experiments involved more than one trial, and PES becomes a vector of values across trials. Dividing this vector with (1-*wf*) will not alter what voxels have significant parameter estimates (i.e. $b \neq 0$), i.e. significant associations with PES, but the magnitude of the parameter estimate now becomes more easily interpretable across participants.

The same logic of course applies to PEM. Because PEM=OutM**wf*-EVM, here we need to divide PEM by *wf* to make parameter estimates interpretable across individuals. The same logic also applies to EVS and EVM, that need to be scaled by (1-*wf*) and *wf*, respectively, to make their parameter estimates suited for an analysis regarding the dependence on *wf*.

Supplementary Table 7 shows a numerical example across our 25 participants considering the first shock trial for PES. In this example we took the actual *wf* estimate of our 25 participants, again only considering the first trial of a block with OutS=-1. BOLD1 is, as above, a hypothetical BOLD response in a voxel where it is constant across participants witnessing the same difference between outcome and expected value, and BOLD2 is, as above, a response in a voxel where the response linearly depends on wf, with BOLD2=(1-*wf*). We then added noise to the BOLD response (uniform random noise between 0 and 0.2), and calculated the parameter estimates b1 (for BOLD1) and b2 (for BOLD2), either given PES or PES/(1-*wf*). Finally, we calculated the correlation between *wf* and the parameter estimates, as we will in the fMRI analysis at the second level, to infer whether the BOLD response in a network or voxel does, or does not, depend linearly on the participants' preferences as captured by *wf*. As can be seen, using PES/(1-*wf*), the correlation is close to zero for BOLD1 (r=0.06) and very high for BOLD2 (r=0.98), and a Bayesian test provides evidence of absence for b1 (BF_{10}<⅓) and evidence of presence of an association for *b*2 (BF_{10}=1.6E14). When using PES directly, however, we find significant, but intermediate associations for *b*1 and b2, despite the very different BOLD situations. This illustrates that using the PES values from M2Out directly does not provide parameter estimate values in fMRI that lend themselves to be easily interpreted with respect to the relationship between BOLD activity magnitude and *wf*.

An alternative approach may have been to standardize the PES vector prior to entering it into the model, as that would also tend to bring the PES predictors to be similar in scale across participants. However, in our research we favored the division by (1-*wf*) and *wf* for PES/EVS and PEM/EVM, respectively, as this ensures that the transformed predictors uses the same units as we used for outcomes (with a PES value of -1 on the first high-shock) rather than depending on the overall variance of the prediction errors.

Subj	*wf*	OutS	PES	PES/<br>(1-*wf*)	BOLD<br>1	BOLD<br>2	noise	b1| PES	b1| PES/(1-*wf*)	b2| PES	b2| PES/(1-*wf*)
1	0.03	-1	-0.97	-1	1	0.97	0.17	-1.21	-1.17	-1.18	-1.14
2	0.95	-1	-0.05	-1	1	0.05	0.16	-24.78	-1.16	-4.46	-0.21
3	0.56	-1	-0.44	-1	1	0.44	0.05	-2.41	-1.05	-1.12	-0.49
4	0.88	-1	-0.12	-1	1	0.12	0.00	-8.06	-1.00	-1.00	-0.12
5	0.05	-1	-0.95	-1	1	0.95	0.12	-1.19	-1.12	-1.13	-1.07
6	0.29	-1	-0.71	-1	1	0.71	0.02	-1.45	-1.02	-1.03	-0.73
7	0.78	-1	-0.22	-1	1	0.22	0.12	-4.98	-1.12	-1.53	-0.34
8	0.11	-1	-0.89	-1	1	0.89	0.06	-1.20	-1.06	-1.07	-0.95
9	0.42	-1	-0.58	-1	1	0.58	0.00	-1.73	-1.00	-1.01	-0.58
10	0.08	-1	-0.92	-1	1	0.92	0.07	-1.17	-1.07	-1.08	-0.99
11	0.48	-1	-0.52	-1	1	0.52	0.14	-2.20	-1.14	-1.27	-0.65
12	0.02	-1	-0.98	-1	1	0.98	0.07	-1.08	-1.07	-1.07	-1.05
13	0.64	-1	-0.36	-1	1	0.36	0.18	-3.30	-1.18	-1.51	-0.54
14	0.68	-1	-0.32	-1	1	0.32	0.18	-3.67	-1.18	-1.57	-0.51
15	0.11	-1	-0.89	-1	1	0.89	0.07	-1.19	-1.07	-1.08	-0.96
16	0.25	-1	-0.75	-1	1	0.75	0.17	-1.56	-1.17	-1.23	-0.93
17	0.97	-1	-0.03	-1	1	0.03	0.07	-37.86	-1.07	-3.59	-0.10
18	0.51	-1	-0.49	-1	1	0.49	0.11	-2.25	-1.11	-1.21	-0.60
19	0.98	-1	-0.02	-1	1	0.02	0.11	-49.32	-1.11	-6.05	-0.14
20	0.07	-1	-0.93	-1	1	0.93	0.19	-1.28	-1.19	-1.21	-1.12
21	0.03	-1	-0.97	-1	1	0.97	0.11	-1.14	-1.11	-1.11	-1.08
22	0.03	-1	-0.97	-1	1	0.97	0.17	-1.21	-1.17	-1.18	-1.14
23	0.08	-1	-0.92	-1	1	0.92	0.19	-1.29	-1.19	-1.21	-1.11
24	0.08	-1	-0.92	-1	1	0.92	0.04	-1.12	-1.04	-1.04	-0.96
25	0.51	-1	-0.49	-1	1	0.49	0.17	-2.38	-1.17	-1.34	-0.66
r(wf,b)								-0.7	0.06	-0.66	0.98
BF10								239	**0.26**	85	**1.60E+14**
p								1.40E-04	0.78	2.00E-04	1.30E-17

Supplementary Table 7: Impact of dividing PES by (1-*wf)* on the interpretability of parameter estimates.
For each of our 25 participant, from left to right: an arbitrary subject number; the *wf* value estimated using M2Out; the OutS value on the first trial, that we assume to be -1 (high-shock); the PES value as calculated using the equation of M2Out (PES=OutS*(1-*wf*)-EVS), knowing that EVS=0 on the first trial of a block; the PES/(1-*wf*) value; BOLD1 response considered to be fixed at 1 across all participants, BOLD2 response calculated as (1-*wf*), noise to be added to the BOLD response, the parameter estimates calculated for BOLD1 (b1) or BOLD2 (b2) given the predictor PES or PES/(1-*wf*). The bottom of the table indicates the correlation between wf and the above parameter estimates together with the BF_{10} and *p* value obtained using JASP.

Supplementary Note 16. Additional fMRI results.

Cluster size	Voxels in cyto	% Cluster	Hem	Cyto or Anatomical description	% Area	Peak *t*-value	MNI coordinates		
							x	y	z
**Main effect of Outcome** (Outcome>0; 5% FWE correction . *t*=3.47 . *p*<.001 . k=903)									
35282	953.5	2.7	left	Lobule VIIa crusI (Hem)	31.4				
	812	2.3	left	Lobule VI (Hem)	43.3				
	688.1	2	right	Lobule VI (Hem)	38.2				
	557	1.6	right	Area 45	53.9				
	502.3	1.4	right	Area hOc4la	56.6				
	496.1	1.4	left	Area hOc4la Middle Occipital Gyrus	58	12.17	-44	-80	-2
	484.5	1.4	right	Lobule VIIa crusI (Hem)	14.9				
	482.4	1.4	right	Area PFm (IPL)	68.4				
	443.6	1.3	left	Thal: Prefrontal	70.3				
	430.1	1.2	right	Thal: Prefrontal	76.9				
	389.1	1.1	left	Area hOc4lp	45.5				
	361.6	1	right	Area hIP3 (IPS)	79.3				
	352.3	1	left	Lobule VIIa crusII (Hem)	21.6				
	342.8	1	right	Area FG4	70				
	328.1	0.9	left	Area FG3 Fusiform Gyrus	39.7	5.16	-42	-52	-20
	323.1	0.9	left	Area hOc4v [V4(v)]	44.5				
	314.3	0.9	right	Area 44	52.4				
	311.6	0.9	right	Area hOc4v [V4(v)]	50.1				
	282.9	0.8	right	Area hOc4lp	50.5				
	271.1	0.8	right	Area hOc1 [V1]	13.1				
	270.1	0.8	right	Area hIP1 (IPS)	93.3				
	261.4	0.7	left	Area FG2	51.3	12.9	-42	-68	-18
	251.8	0.7	right	Area hOc3v [V3v]	29.5				
	232.1	0.7	right	Area PGa (IPL)	31.3				
	221.6	0.6	right	Area FG3 Fusiform Gyrus	33.8	12.73	44	-54	-20
	214.9	0.6	left	Area FG4	36.4				
	205	0.6	right	Area FG2	63				
	200.5	0.6	right	Area FG1	80.6				
	199	0.6	right	Thal: Temporal	36.4				
	192.1	0.5	left	Area hOc3v [V3v]	20.7				
	177.9	0.5	right	Area hIP2 (IPS)	84.4				
	176.1	0.5	left	Area FG1	69.1				
	172.8	0.5	right	Area PF (IPL)	25.6				
	166.6	0.5	left	Lobule IX (Hem)	26.8				
	161.5	0.5	right	Thal: Parietal	48.5				
	160.1	0.5	left	Area 44	18.3				
	153.9	0.4	left	Lobule VI (Verm)	73.5				
	138.9	0.4	right	Lobule VI (Verm)	59.8				
	138.1	0.4	left	Area hOc1 [V1]	6.8				

132.9	0.4	left	Thal: Parietal	41.8	14.44	-18	-28	-4
132.1	0.4	left	Lobule VIIIa (Verm)	89.3				
125.1	0.4	left	Thal: Temporal	23.5	14.17	-24	-26	-6
122.9	0.3	right	Lobule VIIIa (Verm)	58.6				
119.5	0.3	right	Lobule IX (Hem)	17				
110.4	0.3	right	Lobule VIIa crusII (Hem)	7.8				
94.1	0.3	left	Lobule IX (Verm)	105				
91.6	0.3	right	Area hOc2 [V2]	9				
90.8	0.3	right	Lobule IX (Verm)	86.5				
90.1	0.3	left	Lobule VIIb (Hem)	13.3				
87.3	0.2	left	Area 45	12.5				
80.8	0.2	left	Area hOc5 [V5/MT]	100.5				
76.4	0.2	right	Area hOc3d [V3d]	13.9				
72.5	0.2	right	Area Fp1	4.3				
67.5	0.2	right	Amygdala (LB)	31.6				
67.1	0.2	left	Area hOc3d [V3d]	6.8				
62.5	0.2	right	Area 7A (SPL)	8				
61.6	0.2	right	Lobule VIIb (Hem)	9.4				
58.3	0.2	right	Area hOc5 [V5/MT]	100				
56.6	0.2	right	Lobule V (Hem)	7.1				
53	0.2	left	Thal: Visual	59.1				
48.1	0.1	right	Area PFcm (IPL)	14.8				
47.1	0.1	right	Area PFt (IPL)	11.3				
45.9	0.1	right	Area 7PC (SPL)	10.1				
43.9	0.1	right	Thal: Visual	105.4	12.91	24	-28	-4
42.8	0.1	left	Area hOc2 [V2] Middle Occipital Gyrus	4.5	12.81	-18	-102	0
42	0.1	left	Area PFm (IPL)	7.2				
39.3	0.1	right	Subiculum	10.3				
38.9	0.1	left	Area PGa (IPL)	6.1				
37.9	0.1	left	Lobule VIIIa (Hem)	5				
37.6	0.1	right	Area PGp (IPL)	3.8				
36	0.1	left	Lobule VIIb (Verm)	117.6				
35.1	0.1	left	Lobule V (Hem)	4.8				
34	0.1	right	Area 2	5.2				
33.9	0.1	right	Lobule VIIa crusII (Verm)	59.7				
32.1	0.1	right	Lobule I IV (Hem)	6.4				
32	0.1	right	Lobule VIIb (Verm)	97.7				
26.8	0.1	left	Lobule VIIa crusII (Verm)	59.9				
26.6	0.1	right	Thal: Premotor	20				
26.5	0.1	right	Area hOc4d [V3A]	6.3				
24.9	0.1	right	Lobule VIIIa (Hem)	3.4				
23.3	0.1	right	Area Id1	14.2				
22.4	0.1	right	Area Fo2	2				
20.5	0.1	right	Amygdala (CM)	74.9				
20.4	0.1	left	Subiculum	5.3				

	19.6	0.1	left	Thal: Premotor	16.5				
	19.5	0.1	right	Amygdala (SF)	40.9				
	18.1	0.1	left	Area PF (IPL)	3.5				
	16	0	left	Lobule VIIIb (Verm)	25.9				
	15.4	0	right	Lobule VIIIb (Hem)	2.1				
	14.8	0	left	Area PFcm (IPL)	4.6				
	14.8	0	left	Amygdala (LB)	6.1				
	13.3	0	left	Lobule I IV (Hem)	2.8				
	13.1	0	left	Amygdala (CM)	30.3				
	12.9	0	right	Lobule VIIIb (Verm)	17.9				
	12.5	0	right	Thal: Somatosensory	15.4				
	12.1	0	right	BF (Ch 4)	29.1				
	10.9	0	right	DG (Hippocampus)	8.4				
	8.8	0	left	Area PGp (IPL)	1.1				
	8.4	0	right	Area TE 3	0.8				
	8.1	0	left	Thal: Somatosensory	26.1				
	7.6	0	right	Lobule X (Verm)	43.6				
	7.1	0	right	Area 1	1				
	6	0	left	Area hOc4d [V3A]	1.1				
	5.9	0	right	HATA Region	26.9				
	5.8	0	left	Area Id1	4.9				
	5.8	0	left	Lobule X (Verm)	43				
	4.9	0	right	CA1 (Hippocampus)	1.7				
	4.6	0	left	Amygdala (SF)	12.7				
	4.5	0	left	Thal: Motor	9.1				
			right	Insula		13.72	42	20	-2
2380			right	Superior Medial Gyrus	11.41	4	32	52	11.41
			left	Superior Medial Gyrus	09.06	2	32	42	9.06
			right	MCC	8.62	4	30	38	8.62
			right	Posterior-Medial Frontal	6.68	12	26	56	6.68
			right	ACC	6.38	4	36	26	6.38
2345	389.8	16.6	left	Area hIP3 (IPS) Inferior Parietal Lobule	85.1	11.63	-30	-52	46
	279.1	11.9	left	Area hIP1 (IPS)	76.7				
	142.1	6.1	left	Area hIP2 (IPS) Inferior Parietal Lobule	63.2	5.58	-50	-42	46
	104	4.4	left	Area 7A (SPL)	8.3				
	53	2.3	left	Area 7P (SPL) Precuneus	15.2	4.46	8	-76	50
	27.6	1.2	left	Area PFt (IPL)	4.7				
	24.5	1	left	Area 7M (SPL)	15.9				
	21	0.9	right	Area 7P (SPL) Precuneus	4.5	04.01	8	-76	50
	19.8	0.8	right	Area 7M (SPL)	19.3				
	12.1	0.5	left	Area PF (IPL)	2.3				
	12	0.5	left	Area 2	2.3				

	7	0.3	left	Area PFm (IPL)	1.2				
	6.5	0.3	left	Area 7PC (SPL)	3.8				
	6.5	0.3	right	Area 7A (SPL)	0.8				
	0.3	0	left	Area 5L (SPL)	0				
903	42.9	4.7	right	Area 33 ACC	19.8	10.89	6	4	28
	24.9	2.8	left	Area 33 ACC	11.7	09.03	-4	2	28

Supplementary Table 8. BOLD activity associated with the main effect of Outcome.

Only clusters surviving a 5% FWE correction at the cluster size are reported (t=3.47, p<.001, cluster size 903; resampled voxel size: 2x2x2mm; Supplementary Fig. 7a). Brain regions are identified using the Anatomy Toolbox[30]. The columns refer to the size in voxels of each cluster; the number of voxels of that cluster falling within a specific cytoarchitectonic region; the percentage of voxels in that region; hemisphere; cytoarchitectonic region (if available) or macro-anatomical description of the region; percentage of cytoarchitectonic region activated by cluster; peak t-value within a particular region; and MNI coordinates of the peak. If more peaks were identified within the same cyto-architectonic or anatomical region only the peak with the highest t-value was included in the table. Peaks falling outside the gray matter are not included in the table. Cyto architectonic description is only reported when a voxel has a probability over 40% to fall in that area and only for cyto-architectonic areas available in the anatomy toolbox; anatomical description is otherwise reported. The '*.txt' file generated by the Anatomy toolbox can be found at: https://osf.io/rk8w4/?view_only=98b193a58aff48dda40b9d3d91ac5254

		normality		versus zero							correlation with *wf*		
	**test**	**Shapiro**	***p***	***t***	***w***	**df**	***p*(*t*)**	***p*(w)**	**BF_{10}(t)**	**BF_{10}(w)**	**Tau**	**$p_{(Tau)}$**	**$BF_{10(Tau)}$**
AVPS	PESxAVPS	0.967	0.567	-5.461	19	24	0.00001	0.00002	1702.94	416.25	0.01	0.944	0.257
	PEMxAVPS	0.964	0.505	1.041	197	24	0.308	0.367	0.34	0.37	-0.03	0.833	0.262
	EVSxAVPS	0.961	0.441	0.899	199	24	0.377	0.339	0.3	0.31	-0.29	0.042	1.855
	EVMxAVPS	0.904	0.022	2.806	248	24	0.01	0.02	4.86	6.21	-0.043	0.761	0.268
RS	PESxRS	0.984	0.955	3.278	267	24	0.003	0.004	12.71	51.29	-0.19	0.183	0.601
	PEMxRS	0.987	0.981	2.816	258	24	0.01	0.009	4.96	13.38	0.15	0.293	0.436
	EVSxRS	0.938	0.134	-2.052	100	24	0.051	0.096	1.26	1.12	-0.037	0.797	0.265
	EVMxRS	0.897	0.016	-0.581	130	24	0.567	0.396	0.25	0.32	0.157	0.272	0.458

Supplementary Table 9: Supplementary Signature Analyses.

For both the AVPS (top) and RS (bottom) signature, the table details for each loading the result of a shapiro normality test, including the test value and associated p value, followed by a two-tailed test of the loading against zero, where t indicates a student t-test and W a wilcoxon test with the latter particularly relevant for cases where normality is violated. A (t) following p or BF_{10} specifies that these values come from the student t-test, a (w), from the Wilcoxon test. The final 3 columns reflect a Kendall's τ test of the correlation between *wf* and the loading. Kendall's τ was used because of the non-normal distribution of *wf*. Green numbers highlight significant results (p values below 0.05 or BF_{10} values above 3), red values indicate evidence of absence (BF10<⅓).

Cluster size	Voxels in cyto	% Cluster	Hem	Cyto or Anatomical description	% Area	Peak *t*-value	MNI coordinates		
							x	y	z
**Linear regression of PE_S and 1-*wf*** (5% FWE correction *t*=3.47 *p*<.001 k=181)									
219	28	12.8	right	Area s32<br>Mid Orbital Gyrus	19.2	4.07	6	36	-14
	5.8	2.6	right	Area Fo2	0.5				
	4.6	2.1	right	Area s24	3.2				
	2.4	1.1	right	Area Fo2	0.6				
	1.6	0.7	right	Area 33	0.8				
			right	Insula		4.58	28	14	-18
			right	Rectal Gyrus		5.03	20	18	-12
			right	Mid Orbital Gyrus		4.07	6	36	-14
181	52.1	28.8	left	Area 4a<br>Precentral Gyrus	5.6	4.74	-36	-28	66
	45.9	25.3	left	Area 4p<br>Postcentral Gyrus	14.1	4.52	-38	-22	52
	27.5	15.2	left	Area 3b	4.9				
	2	1.1	left	Area 1<br>Postcentral Gyrus	0.4	3.71	-44	-26	60

Supplementary Table 10. BOLD activity covarying with PE_S in a way that depends linearly on *wf*. Note that because the PES values from M2Out have been divided by 1-*wf* prior to entering them into the first level parametric regressor, the PES parameter estimates would no longer depend on 1-wf if PES signals were similarly strong across participants. Only clusters surviving a 5% FWE correction at the cluster size are reported (*t*=3.47, *p*<.001, cluster size 181; resampled voxel size: 2x2x2mm; Supplementary Fig. 7b). Brain regions are identified using the Anatomy Toolbox[30]. The columns refer to the size in voxels of each cluster; the number of voxels of that cluster falling within a specific cytoarchitectonic region; the percentage of voxels in that region; hemisphere; cytoarchitectonic region (if available) or macro-anatomical description of the region; percentage of cytoarchitectonic region activated by cluster; peak t-value within a particular region; and MNI coordinates of the peak. If more peaks were identified within the same cyto-architectonic or anatomical region only the peak with the highest t-value was included in the table. Peaks falling outside the gray matter are not included in the table. Cyto architectonic description is only reported when a voxel has a probability over 40% to fall in that area and only for cyto-architectonic areas available in the anatomy toolbox; anatomical description is otherwise reported. The '*.txt' file generated by the Anatomy toolbox can be found at: https://osf.io/rk8w4/?view_only=98b193a58aff48dda40b9d3d91ac5254

Cluster size	Voxels in cyto	% Cluster	Hem	Cyto or Anatomical description	% Area	Peak *t*-value	MNI coordinates		
							x	y	z
**Linear regression of PE_S** (5% FWE correction t=3.47 p<.001 k=167)									
503	28.3	5.6	right	Area Fp1 Middle Frontal Gyrus	1.7	4.90	26	50	4
	10.4	2.1	left	Area Fp2 ACC	1.4	4.14	-4	50	-2
	3.6	0.7	left	Area Fp2	0.3				
	3.3	0.6	right	Area 33	1.5				
	0.1	0	right	Area s32	0.1				
			right	ACC		4.63	10	38	-2
			left	ACC		4.49	-6	46	-4
			left	Mid Orbital Gyrus		4.35	0	42	-8
167			left	IFG (p. Triangularis)		4.41	-34	40	0
			left	Middle Frontal Gyrus		5.62	-36	52	4
**Linear regression of PE_S** (5% FWE correction t=2.5 p<.01 k=950)									
19882	418.9	2.1	right	Lobule VI (Hem)	23.2				
	353.9	1.8	left	Lobule VI (Hem)	18.9				
	349.8	1.8	right	Lobule VIIa crusI (Hem)	10.8				
	326.3	1.6	right	Area 2	50.2				
	307.4	1.5	right	Area 4a	28.2				
	301.4	1.5	left	Area hOc1 [V1]	14.9				
	289.6	1.5	left	Area 4a	31				
	264.9	1.3	right	Area 3b Postcentral Gyrus	42.1	4.88	18	-38	60
	250.9	1.3	left	Area hOc3d [V3d]	25.3				
	244	1.2	left	Area 3b	43.3				
	215.9	1.1	left	Area 2 Postcentral Gyrus	41	5.76	-40	-34	48
	215.1	1.1	left	Lobule VIIa crusI (Hem)	7.1				
	176.9	0.9	right	Lobule V (Hem)	22.2				
	173.5	0.9	right	Area hOc1 [V1]	8.4				
	163.8	0.8	left	Area hOc3v [V3v]	17.7				
	155.5	0.8	left	Area hOc4v [V4(v)]	21.4				
	139.1	0.7	right	Area 4p	44.7				
	132.8	0.7	right	Area Fp1 Middle Frontal Gyrus	7.8	4.90	26	50	4
	125.1	0.6	left	Area 4p	38.6				
	115.1	0.6	right	Area PGp (IPL) Angular Gyrus	11.7	4.84	42	-72	36
	111.3	0.6	right	Area hIP2 (IPS)	52.8				
	111.3	0.6	left	Area s32	53.1				
	110.4	0.6	left	Area hOc4d [V3A]	19.3				
	110.4	0.6	right	Area hIP3 (IPS)	24.2				
	106.8	0.5	right	Area hOc4d [V3A]	25.4				
	102.8	0.5	left	Lobule VIIa crusII (Hem)	6.3				
	102.3	0.5	right	Area 5M (SPL)	34.9				
	96.5	0.5	left	Lobule V (Hem)	13.2				
	95.6	0.5	right	Area 3a	47.5				
	94.6	0.5	left	Area 3a	32.9				
	92.6	0.5	left	Area hOc2 [V2]	9.8				
	90.6	0.5	right	Area OP4 [PV]	29				
	80.6	0.4	right	Area 5L (SPL)	11				
	79.4	0.4	left	Area OP3 [VS]	56.6				
	77.8	0.4	left	Area 7A (SPL)	6.2				
	76.6	0.4	right	Area hOc3d [V3d]	13.9				
	74.1	0.4	right	Area s32	50.9				
	72.4	0.4	right	Area TE 1.2	69.4				
	70.5	0.4	left	Area 5M (SPL)	14.5				
	70.4	0.4	right	Area PGa (IPL)	9.5				
	70.1	0.4	left	Lobule VIIIa (Verm)	47.4				
	69.9	0.4	left	Area FG4	11.8				
	69.6	0.4	right	Area PFt (IPL)	16.7				
	68	0.3	right	Area hOc2 [V2]	6.7				

	64.3	0.3	left	Lobule I IV (Hem)	13.3				
	62.6	0.3	left	Area Fp2	8.6				
	61.1	0.3	right	Lobule VIIIa (Verm)	29.2				
	60.3	0.3	left	Area 44	6.9				
	57.5	0.3	right	Area hOc3v [V3v]	6.7				
	56.9	0.3	right	Area 1	8.1				
	56.6	0.3	left	Area PFt (IPL)	9.7				
	51.3	0.3	left	Area Fp1	2.8				
	51	0.3	right	Area Fo2	4.6				
	50.3	0.3	right	Area TE 3	4.8				
	48.6	0.2	left	Area OP4 [PV]	13.5				
	45.6	0.2	right	Area 5Ci (SPL)	23.1				
	45	0.2	right	Lobule VIIa crusII (Hem)	3.2				
	44.5	0.2	left	Area TE 3	5				
	43	0.2	left	Lobule VI (Verm)	20.5				
	39.5	0.2	right	Lobule VIIIb (Verm)	54.8				
	36.4	0.2	right	Lobule VI (Verm)	15.7				
	36.3	0.2	left	Area TE 1.0	29.3				
	35.4	0.2	left	Area 7P (SPL)	10.2				
	34.9	0.2	right	Area hIP1 (IPS)	12				
	33.6	0.2	left	Area 5L (SPL)	4.8				
	32.6	0.2	right	Area 33	15.1				
	31.4	0.2	right	Area FG4	6.4				
	30	0.2	right	Lobule I IV (Hem)	6				
	28.6	0.1	left	Area hOc4lp	3.3				
	27.1	0.1	right	Area TE 1.0	17.9				
	26.6	0.1	left	Area TE 1.1	16.5				
	25.1	0.1	left	Area OP2 [PIVC]	43.2				
	23.4	0.1	left	Area TE 1.2	16.7				
	23.1	0.1	left	Thal: Parietal	7.3				
	22.3	0.1	left	Area PFcm (IPL)	6.9				
	21.1	0.1	right	Area 7A (SPL)	2.7				
	21	0.1	left	Area FG1	8.2				
	19	0.1	left	Lobule IX (Verm)	21.2				
	16	0.1	right	Area 7M (SPL)	15.7				
	15.9	0.1	left	Area Fo2	1.5				
	15.6	0.1	left	Area Ig1	22.7				
	15.6	0.1	left	Area Ig2	11.5				
	15.6	0.1	left	Lobule VIIIb (Verm)	25.3				
	15.5	0.1	left	Area 1	2.7				
	15.4	0.1	right	Area 7PC (SPL)	3.4				
	12.4	0.1	right	Lobule IX (Verm)	11.8				
	11.5	0.1	right	Area PFm (IPL)	1.6				
	11.4	0.1	right	Area Fp2	1.9				
	9.5	0	left	Area 7PC (SPL)	5.6				
	9.1	0	left	Area 33	4.3				
	8.8	0	right	Area hOc4v [V4(v)]	1.4				
	7.5	0	left	Area OP1 [SII]	2				
	6	0	left	Area PGp (IPL)	0.7				
	5.4	0	right	Area s24	3.7				
	5.3	0	left	Lobule VIIb (Verm)	17.1				
	4.6	0	left	Lobule IX (Hem)	0.7				
	4.3	0	right	Lobule VIIb (Verm)	13				
	3.5	0	right	Area OP1 [SII]	0.9				
	3.4	0	right	Lobule IX (Hem)	0.5				
	3.1	0	right	BF (Ch 4)	7.5				
	3.1	0	left	Area s24	1.8				
	2.9	0	right	Lobule VIIIa (Hem)	0.4				
	2.9	0	right	Area OP3 [VS]	1.4				
			right	MCC		4.78	14	-14	50
950	110.1	11.6	right	Area TE 1.1 Superior Temporal Gyrus	54.5	3.07	40	-32	14
	94.3	9.9	right	Area OP3 [VS]	44.9				
	66.4	7	right	Area Ig1 Insula	87	3.57	38	-20	4
	54.1	5.7	right	Area OP2 [PIVC]	61.1				

35.1	3.7	right	Area Ig2	22.1
22.3	2.3	right	Area OP1 [SII]	5.7
22	2.3	right	Thal: Somatosensory	27.1
19.1	2	right	Thal: Motor	43.2
18.4	1.9	right	Thal: Parietal	5.5
8.8	0.9	right	Thal: Premotor	6.6
5	0.5	right	Area PFcm (IPL)	1.5
3.8	0.4	right	Area Id1	2.3
2.6	0.3	right	Thal: Prefrontal	0.5
1	0.1	right	Area TE 1.0	0.7

Supplementary Table 11. BOLD activity covarying positively with PE_S after removing variance explained by *wf*.
Results of the constant in the linear regression of PE_S and 1-*wf*, capturing voxels where the parametric modulator for PES is above zero after removing variance explained by 1-*wf***.** Note that because the PE_S values from M2Out have been divided by 1-*wf* prior to entering them into the first level parametric regressor, the PE_S parameter estimates used in this analysis would survive this contrast particularly well if all participants had similarly positive prediction errors. Clusters surviving a 5% FWE correction at the cluster size are reported (above, *t*=3.47. *p*<.01, cluster size 950; resampled voxel size: 2x2x2mm; Figure 7c) and clusters surviving a 5% FWE correction at the cluster size are reported (below, t=3.47. *p*<.001, cluster size 167; resampled voxel size: 2x2x2mm; Figure 7b). Brain regions are identified using the Anatomy Toolbox[30]. The columns refer to the size in voxels of each cluster; the number of voxels of that cluster falling within a specific cytoarchitectonic region; the percentage of voxels in that region; hemisphere; cytoarchitectonic region (if available) or macro-anatomical description of the region; percentage of cytoarchitectonic region activated by cluster; peak t-value within a particular region; and MNI coordinates of the peak. If more peaks were identified within the same cyto-architectonic or anatomical region only the peak with the highest t-value was included in the table. Peaks falling outside the gray matter are not included in the table. Cyto architectonic description is only reported when a voxel has a probability over 40% to fall in that area and only for cyto-architectonic areas available in the anatomy toolbox; anatomical description is otherwise reported. The '*.txt' file generated by the Anatomy toolbox can be found at: https://osf.io/rk8w4/?view_only=98b193a58aff48dda40b9d3d91ac5254

Cluster size	Voxels in cyto	% Cluster	Hem	Cyto or Anatomical description	% Area	Peak *t*-value	MNI coordinates x	y	z
**Linear regression of PE_M** (5% FWE correction *t*=2.5 *p*<.01 k=542)									
2093	266.6	12.7	left	Thal: Temporal	50.1	4.44	-12	-32	6
	228.6	10.9	right	Thal: Temporal	41.9	4.60	8	-20	14
	142.6	6.8	left	Thal: Prefrontal	22.6	3.93	-18	-8	12
	136.3	6.5	right	Thal: Prefrontal	24.3				
	66	3.2	right	Lobule I IV (Hem)	13.2				
	39.9	1.9	right	Lobule IX (Hem)	5.7				
	39.3	1.9	right	Thal: Parietal	11.8				
	30.6	1.5	left	Subiculum	8				
	23.1	1.1	right	Thal: Somatosensory	28.5	4.53	22	-20	4
	16.5	0.8	left	Lobule I IV (Hem)	3.4				
	15.3	0.7	right	Thal: Premotor	11.5				
	14.4	0.7	left	Thal: Visual	16				
	13.5	0.6	right	Lobule IX (Verm)	12.9				
	11.1	0.5	right	Thal: Motor	25.1				
	8.9	0.4	left	Thal: Parietal	2.8				
	3.3	0.2	right	Thal: Visual	7.8				
	1.6	0.1	left	CA3 (Hippocampus)	4				
	1.6	0.1	right	Lobule V (Hem)	0.2				
	1.6	0.1	left	Thal: Premotor	1.4				
	1.3		left	CA1 (Hippocampus)	0.6				
	1		right	Subiculum	0.3				
			left	ParaHippocampal Gyrus		3.66	-16	-20	-20
1226	375	30.6	left	Lobule VIIa crusI (Hem)	12.3	4.42	-14	-72	-32
	232.6	19	left	Lobule VIIa crusII (Hem)	14.2	4.61	-16	-76	-38
	204.4	16.7	right	Lobule VIIa crusI (Hem)	6.3				
	95.1	7.8	left	Lobule VI (Hem)	5.1	4.07	-20	-66	-34
	67.4	5.5	right	Lobule VI (Hem)	3.7	3.36	30	-64	-28
	28.4	2.3	right	Lobule VIIa crusII (Hem)	2				
	17.3	1.4	left	Lobule VIIa crusII (Verm)	38.7				
	14.5	1.2	right	Lobule VIIa crusII (Verm)	25.6				
	5.3	0.4	left	Lobule VI (Verm)	2.5				
	5	0.4	right	Lobule VIIb (Hem)	0.8				
	4.4	0.4	right	Lobule VI (Verm)	1.9				
	3.6	0.3	right	Lobule VIIb (Verm)	11.1				
	2.3	0.2	left	Lobule VIIb (Hem)	0.3				
	0.5	0	left	Lobule VIIb (Verm)	1.6				
	0.5	0	right	Lobule VIIIa (Verm)	0.2				
	0.3	0	right	Lobule VIIIa (Hem)	0				
	0.1	0	left	Lobule VIIIa (Verm)	0.1				
564	33	5.9	right	Area Fo2	3				
	6.9	1.2	right	Area Fp1 Middle Orbital Gyrus	0.4	2.82	30	52	-6

	6.6	1.2	right	BF (Ch 4)	15.9	2.96	24	-2	-10
	6.6	1.2	right	Area 45	0.6				
	3.5	0.6	right	Amygdala (CM)	12.8				
	0.6	0.1	right	Amygdala (AStr)	4.1				
			right	IFG (p. Orbitalis)		4.31	32	38	-10
			right	Putamen		4.29	30	6	-6
			right	IFG (p. Triangularis)		3.27	48	28	0
			right	Insula Lobe		2.99	38	16	-4
542	4.3	0.8	right	Area 33	2				
	3.5	0.6	right	Area 5Ci (SPL)	1.8				
			left	MCC		4.07	0	-4	34
			right	MCC		3.89	4	-18	34
			left	PCC		3.58	0	-34	32

Supplementary Table 12. BOLD activity covarying positively with PEM after removing variance explained by *wf*. Results of the constant in the linear regression of PE_M and 1-*wf*. capturing voxels where the parametric modulator for PEM is above zero after removing variance explained by 1-*wf***.** Note that because the PE_M values from M2Out have been divided by 1-*wf* prior to entering them into the first level parametric regressor. the PE_M parameter estimates used in this analysis would survive this contrast particularly well if all participants had similarly positive prediction errors. Only clusters surviving a 5% FWE correction at the cluster size are reported (t=3.47. p<.01, cluster size 542; resampled voxel size: 2x2x2mm; Figure 7c). Brain regions are identified using the Anatomy Toolbox[30]. The columns refer to the size in voxels of each cluster; the number of voxels of that cluster falling within a specific cytoarchitectonic region; the percentage of voxels in that region; hemisphere; cytoarchitectonic region (if available) or macro-anatomical description of the region; percentage of cytoarchitectonic region activated by cluster; peak t-value within a particular region; and MNI coordinates of the peak. If more peaks were identified within the same cyto-architectonic or anatomical region only the peak with the highest t-value was included in the table. Peaks falling outside the gray matter are not included in the table. Cyto architectonic description is only reported when a voxel has a probability over 40% to fall in that area and only for cyto-architectonic areas available in the anatomy toolbox; anatomical description is otherwise reported. The '*.txt' file generated by the Anatomy toolbox can be found at: https://osf.io/rk8w4/?view_only=98b193a58aff48dda40b9d3d91ac5254

Cluster size	Voxels in cyto	% Cluster	Hem	Cyto or Anatomical description	% Area	Peak *t*-value	MNI coordinates		
							x	y	z
**Linear regression of PE_M and 1-wf** (5% FWE correction *t*=2.5 *p*<.01 k=1642)									
1642									
	252,9	15,4	right	Lobule VI (Hem) Cerebelum (VI)	14	4.26	8	-68	-16
	169	10,3	right	Lobule V (Hem) Cerebellar Vermis	21.2	4.11	4	-62	-12
	128,1	7,8	left	Lobule VI (Hem)	6.8				
	118,8	7,2	right	Area FG4	24.3				
	59,9	3,6	right	Area FG3 Fusiform Gyrus	9.1	3.80	40	-48	-22
	59,6	3,6	left	Lobule V (Hem)	8.1				
	54,9	3,3	right	Lobule I IV (Hem) Cereb	11	4.11	2	-46	-6
	52	3,2	left	Lobule VI (Verm)	24.8				
	37,4	2,3	right	Lobule VI (Verm) Cerebellar Vermis	16.1	4.32	2	-70	-20
	34,1	2,1	right	DG (Hippocampus)	26.5				
	32,9	2	left	Lobule I IV (Hem)	6.8				
	28	1,7	right	CA1 (Hippocampus)	9.7				
	23,4	1,4	right	Thal: Temporal Hippocampus	4.3	3.98	20	-34	2
	21,5	1,3	right	CA3 (Hippocampus)	36.3				
	18,9	1,1	right	Subiculum	5				
	17,8	1,1	right	Area FG1	7.1				
	11,5	0,7	right	CA2 (Hippocampus)	19.1	3.71	30	-30	-10
	5,4	0,3	right	Area FG2	1.7				
	1,3	0,1	right	Lobule VIIa crusI (Hem)	0				
	1	0,1	right	Thal: Parietal	0.3				
	0,9	0,1	right	Area hOc3v [V3v]	0.1				
	0,4	0	left	Lobule VIIa crusI (Hem)	0				

Supplementary Table 13. BOLD activity covarying with PE_M in a way that depends linearly on *wf*. Results of the linear regression of PE_M and 1-*wf*.

Note that because the PEM values from M2Out have been divided by 1-*wf* prior to entering them into the first level parametric regressor. the PEM parameter estimates would no longer depend on 1-*wf* if PEM signals were similarly strong across participants. Only clusters surviving a 5% FWE correction at the cluster size are reported (*t*=3.47. *p*<.01, cluster size 1642; resampled voxel size: 2x2x2mm; Figure 7d). Brain regions are identified using the Anatomy Toolbox[30]. The columns refer to the size in voxels of each cluster; the number of voxels of that cluster falling within a specific cytoarchitectonic region; the percentage of voxels in that region; hemisphere; cytoarchitectonic region (if available) or macro-anatomical description of the region; percentage of cytoarchitectonic region activated by cluster; peak *t*-value within a particular region; and MNI coordinates of the peak. If more peaks were identified within the same cyto-architectonic or anatomical region only the peak with the highest t-value was included in the table. Peaks falling outside the gray matter are not included in the table. Cyto architectonic description is only reported when a voxel has a probability over 40% to fall in that area and only for cyto-architectonic areas available in the anatomy toolbox; anatomical description is otherwise reported. The '*.txt' file generated by the Anatomy toolbox can be found at: https://osf.io/rk8w4/?view_only=98b193a58aff48dda40b9d3d91ac5254

Cluster size	Voxels in cyto	% Cluster	Hem	Cyto or Anatomical description	% Area	Peak *t*-value	MNI coordinates		
							x	y	z
**Linear regression of PE_S and LR_S** (5% FWE correction *t*=3.47 *p*<.001 k=121)									
1946	37.5	1.9	left	Area 4a	4				
	6.5	0.3	left	Area 5M (SPL)	1.3				
	1.3	0.1	right	Area 33	0.6				
			left	Posterior-Medial Frontal	6.97	-6	-12	72	6.97
			right	Superior Frontal Gyrus	5.72	14	22	48	5.72
			right	Posterior-Medial Frontal	5.22	10	4	66	5.22
			left	Superior Frontal Gyrus	5.11	-14	-6	70	5.11
394			right	Superior Frontal Gyrus		7.62	24	38	36
			right	Middle Frontal Gyrus		6.60	28	36	38
389	61.4	15.8	right	Area 45 IFG (p. Triangularis)	5.9	4.77	56	30	16
			right	Middle Frontal Gyrus		6.68	44	36	22
194	0.8	0.4	left	Area Fp2	0.1	7.28	-12	52	26
			left	Superior Frontal Gyrus		3.79	-10	44	14
			left	ACC		7.28	-12	52	26
137	15.1	11	right	Area 44	2.5	3.78	62	8	22
121			left	Middle Frontal Gyrus		5.98	-22	34	40
			left	Superior Frontal Gyrus		4.65	-16	26	44

Supplementary Table 14. BOLD activity covarying with PE_S in a way that depends linearly on LR_S.
Only clusters surviving a 5% FWE correction at the cluster size are reported (*t*=3.47. *p*<.001, cluster size 121; resampled voxel size: 2x2x2mm; Figure 7e). Brain regions are identified using the Anatomy Toolbox[30]. The columns refer to the size in voxels of each cluster; the number of voxels of that cluster falling within a specific cytoarchitectonic region; the percentage of voxels in that region; hemisphere; cytoarchitectonic region (if available) or macro-anatomical description of the region; percentage of cytoarchitectonic region activated by cluster; peak t-value within a particular region; and MNI coordinates of the peak. If more peaks were identified within the same cyto-architectonic or anatomical region only the peak with the highest t-value was included in the table. Peaks falling outside the gray matter are not included in the table. Cyto architectonic description is only reported when a voxel has a probability over 40% to fall in that area and only for cyto-architectonic areas available in the anatomy toolbox; anatomical description is otherwise reported. The '*.txt' file generated by the Anatomy toolbox can be found at: https://osf.io/rk8w4/?view_only=98b193a58aff48dda40b9d3d91ac5254

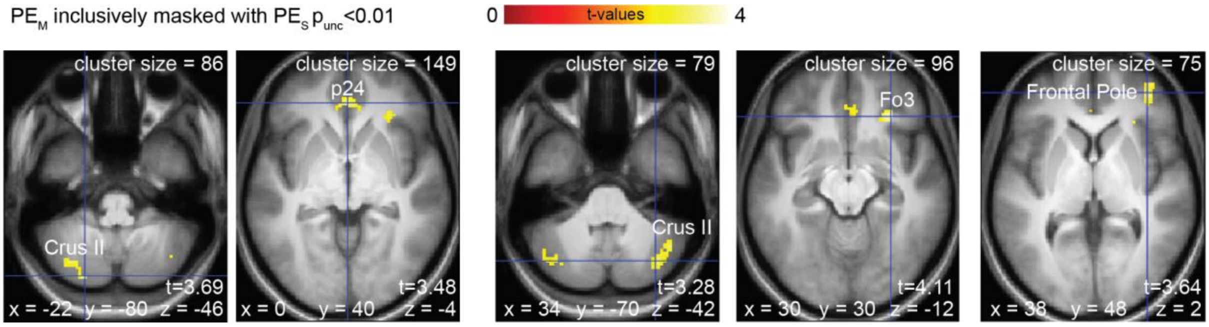

Supplementary Fig. 17. Overlap between PE_M and PE_S.

Cluster of activity obtained by inclusively masking PE_M (after the variance explained by *wf* is removed) with PE_S (after the variance explained by (1-*wf*) is removed). T-values and coordinates in each image correspond to the peak of the cluster indicated by the blue cross-line. Cluster size indicated on top right for each image. Minimum t-threshold corresponding to a one-tailed t-test at $p_{unc}<0.01$ was 2.5. No FWEc was applied. The PEs mask was taken from Figure 7c (purple; $p_{unc}<0.01$, $t=2.5$, k=FWEc=950 voxels). Slices are taken from the average T1 anatomical scan from our participants and visualized with SPM12.The '*.nii' file can be found at: https://osf.io/rk8w4/

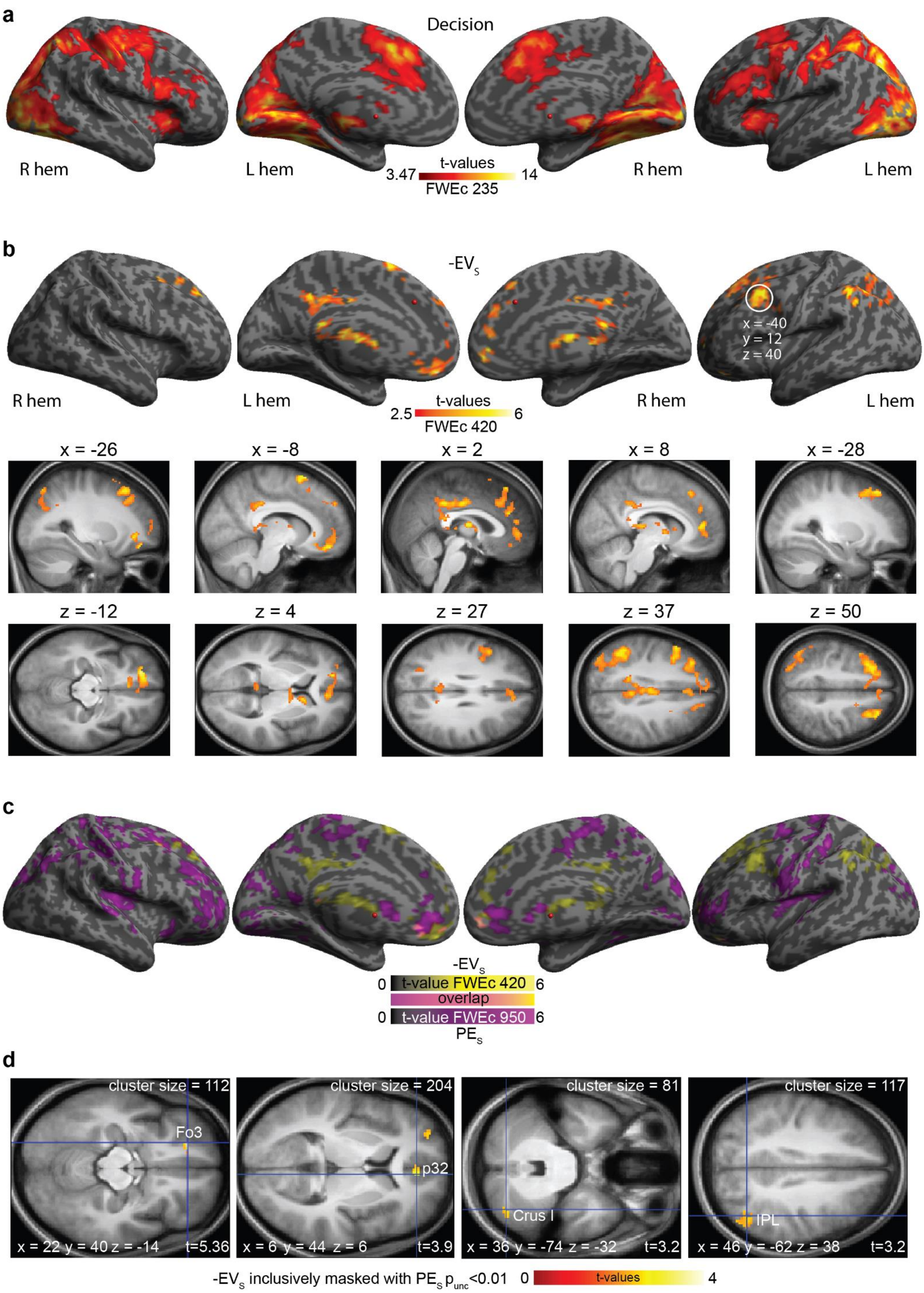
a
Decision
R hem
L hem
t-values
3.47
14
FWEc 235
R hem
L hem
b
-EVs
x = -40
y = 12
z = 40
R hem
L hem
t-values
2.5
6
FWEc 420
R hem
L hem
x = -26
x = -8
x = 2
x = 8
x = -28
z = -12
z = 4
z = 27
z = 37
z = 50
c
-EVs
0
t-value FWEc 420
6
overlap
0
t-value FWEc 950
6
PEs
d
cluster size = 112
Fo3
x = 22 y = 40 z = -14
t=5.36
cluster size = 204
p32
x = 6 y = 44 z = 6
t=3.9
cluster size = 81
Crus I
x = 36 y = -74 z = -32
t=3.2
cluster size = 117
IPL
x = 46 y = -62 z = 38
t=3.2
-EVs inclusively masked with PEs punc<0.01
0
t-values
4

Supplementary Fig. 18. Decision phase and EV. a Results of the second level one-tail t-test Decision>0, indicating voxels where BOLD signals during the decision phase (independently of prediction errors) are increased, $p_{unc}<0.001$, $t=3.47$, k=FWEc=235 voxels. See also supplementary Table 15 **b** BOLD signal negatively correlating with the expected value for shock, one-tail $p_{unc}<0.01$, $t=2.5$, k=FWEc=420 voxels (Supplementary Table 16). The white circle indicates the cluster also surviving at $p_{unc}<0.001$, $t=3.48$, k=FWEc=137 voxels, with the peak at the indicated coordinates. **c** The renders and slices visually compare activity correlating with -EV_S (purple) and PE_S (yellow), both shown at one-tail $p_{unc}<0.01$, $t=2.5$, with each relative FWEc. The images clearly show that -EV_S and PE_S can be dissociated, with several clusters independently correlating with -EV and PE_S, and only four clusters (not surviving an FWEc correction at $p_{unc}<0.01$) overlapping between PE_S and -EV_S. Note that -EV_S is modeled as a parametric modulator of the decision phase, while PE_S as a modulator of the outcome phase. Expected values computed during PE_S cannot be isolated. **d** Overlapping clusters between -EV_S and PE_S at one-tail $p_{unc}<0.01$, $t=2.5$, without any cluster correction. *T*-values and coordinates in each image correspond to the peak of the cluster indicated by the blue cross-line. Cluster size indicated on top right for each image. The PEs mask was taken from Figure 7c (purple; one-tail $p_{unc}<0.01$, $t=2.5$, k=FWEc=950 voxels). Description based on the Anatomy toolbox for SPM, and the statistical whole brain tables from SPM12. As in Figure 7, all results are FWE cluster corrected at $p_{unc}<0.05$, following cluster cutting at $p_{unc}<0.001$ or $p_{unc}<0.01$, specified using the critical FWE cluster size FWEc as indicated in figure panels. Renders were created in SPM12, based on the cortex_20484 surface from the SPM12 templates; slices are taken from the average T1 anatomical scan from our participants and visualized with SPM12. The '*.nii' files can be found at: https://osf.io/rk8w4/

Cluster size	Voxels in cyto	% Cluster	Hem	Cyto or Anatomical description	% Area	Peak *t*-value	MNI coordinates		
							x	y	z
**Main effect of Decision** (5% FWE correction t=3.47 *p*<.001 k=235)									
50325									
	1551.9	3.1	right	Area hOc1 [V1] Lingual Gyrus	75.2	13.16	-12	-90	-4
	1449.8	2.9	left	Area hOc1 [V1] Calcarine Gyrus	71.7	13.28	-12	-90	-4
	1086.5	2.2	left	Lobule VI (Hem)	58				
	910.3	1.8	right	Lobule VI (Hem)	50.5				
	594.8	1.2	right	Area hOc3v [V3v]	69.8				
	587	1.2	left	Area hOc3v [V3v]	63.4				
	570.8	1.1	left	Area hOc4v [V4(v)]	78.5				
	570.1	1.1	right	Area hOc2 [V2]	55.9				
	508.6	1	left	Area 7A (SPL)	40.6				
	502.3	1	left	Area hOc2 [V2]	53.1				
	499.9	1	left	Area FG4 Fusiform Gyrus	84.7	12.96	-28	-54	-14
	496.4	1	left	Area hOc4la	58.1				
	496	1	right	Area hOc4la	55.9				
	495.9	1	left	Lobule V (Hem)	67.7				
	484	1	right	Area 2	74.4				
	478.6	1	left	Area hOc4lp Middle Occipital Gyrus	55.9	13.16	-28	-88	2
	448.4	0.9	right	Area FG4	91.6				
	418.8	0.8	right	Area hOc4v [V4(v)]	67.4				
	403.8	0.8	left	Area PFt (IPL)	69.3				
	394.9	0.8	left	Thal: Prefrontal	62.6				
	381	0.8	left	Area 2	72.3				
	359.5	0.7	right	Thal: Prefrontal	64.2				
	359.1	0.7	left	Area 44	41				
	345	0.7	left	Area hIP3 (IPS)	75.3				
	328.4	0.7	right	Area hIP3 (IPS)	72				
	323	0.6	left	Area FG3 Fusiform Gyrus	39.1	12.85	-42	-60	-14
	322	0.6	left	Area hOc3d [V3d]	32.5				
	295.5	0.6	left	Area hOc4d [V3A]	51.8				
	287.9	0.6	right	Area 3b	45.8				
	286.8	0.6	right	Area hOc4lp	51.2				
	283.6	0.6	right	Area 1	40.4				
	253.9	0.5	left	Area FG1	99.6				
	247	0.5	right	Area 44	41.2				
	245.9	0.5	right	Area hOc3d [V3d]	44.7				

244.1	0.5	right	Area FG1	98.2				
243.5	0.5	left	Area FG2 Fusiform Gyrus	47.8	13.32	-40	-66	-12
241.5	0.5	right	Area PFt (IPL)	57.9				
224.3	0.4	right	Area 7A (SPL)	28.8				
221.6	0.4	right	Area FG3	33.8				
210.5	0.4	right	Thal: Parietal	63.2				
205.1	0.4	right	Area 4p	66				
200.1	0.4	right	Lobule VIIa crusI (Hem)	6.2				
198.6	0.4	right	Area 7PC (SPL)	43.7				
194.8	0.4	right	Area FG2 Inferior Occipital Gyrus	59.9	14.03	46	-62	-14
194	0.4	left	Area 3b	34.4				
191.8	0.4	right	Lobule V (Hem)	24				
169.9	0.3	left	Area hIP1 (IPS)	46.7				
164.8	0.3	left	Thal: Parietal	51.8				
158.8	0.3	right	Area hOc4d [V3A]	37.8				
140.9	0.3	left	Thal: Temporal	26.5				
140.9	0.3	right	Area 45	13.6				
121.1	0.2	left	Area 7PC (SPL)	71				
120.4	0.2	left	Area hIP2 (IPS)	53.6				
119.3	0.2	left	Area 45	17.1				
114.6	0.2	left	Lobule VIIIa (Verm)	77.4				
111.6	0.2	left	Lobule VI (Verm)	53.3				
99.8	0.2	left	Lobule VIIa crusI (Hem)	3.3				
98.9	0.2	right	Area hIP2 (IPS)	46.9				
98.9	0.2	right	Lobule VI (Verm)	42.6				
96.3	0.2	right	Thal: Temporal	17.6				
90.4	0.2	right	Area 3a	44.9				
84.9	0.2	right	Thal: Premotor	63.8				
81.5	0.2	left	Area hOc5 [V5/MT]	101.4				
80.1	0.2	right	Subiculum	21.1				
78.8	0.2	left	Area 1	13.8				
78.1	0.2	left	Area 7P (SPL)	22.4				
75.9	0.2	left	Area 5L (SPL)	10.9				
60.9	0.1	right	Lobule IX (Verm)	58				
58.4	0.1	left	Lobule IX (Verm)	65.1				
56.6	0.1	right	Area hIP1 (IPS)	19.6				
55.4	0.1	left	Area 33	26.1				
54	0.1	right	Lobule VIIIa (Verm)	25.8				
52.8	0.1	left	Lobule I IV (Hem)	11				
47.6	0.1	left	Thal: Premotor	40.1				
47	0.1	left	Thal: Visual	52.4	12.69	-24	-28	-4
45.4	0.1	right	Area hOc5 [V5/MT]	77.9				
45.3	0.1	right	Thal: Somatosensory	55.7				
44.4	0.1	right	Area 33	20.5				
38.3	0.1	right	Area 4a	3.5				
36.6	0.1	left	Area 3a	12.7				
34.8	0.1	right	Area 7P (SPL)	7.4				
32.6	0.1	right	Lobule VIIb (Hem)	5				
32.5	0.1	right	Thal: Visual	78.1				
31.6	0.1	left	Subiculum	8.3				
30	0.1	left	Lobule VIIb (Hem)	4.4				
29.3	0.1	right	Lobule VIIa crusII (Hem)	2.1				
28.9	0.1	left	Lobule VIIb (Verm)	94.3				
28.1	0.1	left	Area 7M (SPL)	18.2				
27.5	0.1	right	Lobule IX (Hem)	3.9				
27.1	0.1	right	Area PGp (IPL)	2.7				
26.8	0.1	left	Lobule VIIIa (Hem)	3.5				
26.5	0.1	left	Area PFop (IPL)	11.9				
25.1	0	right	Area 5L (SPL)	3.4				

	22.3	0	left	Thal: Motor	44.8				
	20.3	0	left	Lobule VIIIb (Verm)	32.8				
	19.4	0	right	Lobule VIIb (Verm)	59.2				
	18.6	0	left	Area PGp (IPL)	2.2				
	17.4	0	left	Thal: Somatosensory	55.8				
	17.3	0	right	Area 7M (SPL)	16.9				
	14.1	0	right	Lobule I IV (Hem)	2.8				
	13.4	0	right	Thal: Motor	30.2				
	8.5	0	right	DG (Hippocampus)	6.6				
	8.1	0	right	Lobule VIIIa (Hem)	1.1				
	6.5	0	left	Area PF (IPL)	1.2				
	5.9	0	right	Lobule VIIa crusII (Verm)	10.4				
	5.8	0	left	Lobule VIIIb (Hem)	0.9				
	5.1	0	right	CA1 (Hippocampus)	1.8				
235				Middle Frontal Gyrus		4.92	38	42	28

Supplementary Table 15. BOLD activity associated with the main effect of Decision.

Only clusters surviving a 5% FWE correction at the cluster size are reported (t=3.47. p<.001, cluster size 235; resampled voxel size: 2x2x2mm; Figure 18a). Brain regions are identified using the Anatomy Toolbox[30]. The columns refer to the size in voxels of each cluster; the number of voxels of that cluster falling within a specific cytoarchitectonic region; the percentage of voxels in that region; hemisphere; cytoarchitectonic region (if available) or macro-anatomical description of the region; percentage of cytoarchitectonic region activated by cluster; peak t-value within a particular region; and MNI coordinates of the peak. If more peaks were identified within the same cyto-architectonic or anatomical region only the peak with the highest t-value was included in the table. Peaks falling outside the gray matter are not included in the table. Cyto architectonic description is only reported when a voxel has a probability over 40% to fall in that area and only for cyto-architectonic areas available in the anatomy toolbox; anatomical description is otherwise reported. The '*.txt' file generated by the Anatomy toolbox can be found at: https://osf.io/rk8w4/?view_only=98b193a58aff48dda40b9d3d91ac5254

Cluster size	Voxels in cyto	% Cluster	Hem	Cyto or Anatomical description	% Area	Peak *t*-value	MNI coordinates		
							x	y	z
**Linear regression of -EV$_s$** (5% FWE correction t=2.5 p<.01 k=420)									
3158	80.8	2.6	left	Area Fp2 Superior Orbital Gyrus	11.1	5.42	-24	42	-14
	79.8	2.5	left	Area 44	9.1				
	69.1	2.2	left	Area s32	33				
	36.5	1.2	left	Area Fp1	2				
	22.9	0.7	left	Area Fo2	2.2				
	13.5	0.4	right	Area Fp2	2.2				
	11.8	0.4	left	Area Fo1	2.5				
	7.3	0.2	right	Area s32	5				
	6.4	0.2	left	Area s24	3.8				
	5.3	0.2	left	Area 33	2.5				
	3.3	0.1	left	Area 45	0.5				
	0.5	0	right	Area 33	0.2				
	0.4	0	right	Area s24	0.3				
			left	Posterior-Medial Frontal		5.15	-14	16	66
			left	Middle Frontal Gyrus		4.56	-6	12	62
1454	94.6	6.5	left	Thal: Temporal	17.8	4.78	-2	-12	10
	72.9	5	right	Thal: Temporal	13.3	4.90	2	-10	12
	27.5	1.9	right	Thal: Prefrontal	4.9	4.33	6	-24	8
	20.1	1.4	left	Thal: Prefrontal	3.2				
	0.3	0	left	Thal: Parietal	0.1				

			left	MCC		4.33	0	-18	36
			right	Caudate Nucleus		3.99	10	12	6
1114	197.5	17.7	left	Area hIP1 (IPS) Inferior Parietal Lobule	54.2	3.99	-40	-46	36
	144.9	13	left	Area hIP3 (IPS)	31.6				
	124	11.1	left	Area hIP2 (IPS) Inferior Parietal Lobule	55.2	4.15	-48	-48	42
	93	8.3	left	Area PGa (IPL) Angular Gyrus	14.6	3.40	-42	-62	46
	91	8.2	left	Area PFm (IPL) Inferior Parietal Lobule	15.7	3.50	-40	-62	52
	45.3	4.1	left	Area 7A (SPL)	3.6				
	41.4	3.7	left	Area PGp (IPL) Angular Gyrus	5	3.40	-34	-62	38
	6.4	0.6	left	Area PF (IPL)	1.2				
	3.9	0.3	left	Area PFt (IPL)	0.7				
	3	0.3	left	Area 2	0.6				
	0.3	0	left	Area PFcm (IPL)	0.1				
	0.1	0	left	Area 7P (SPL)	0				
			left	Superior Parietal Lobule		3.50	-26	-72	52
459	338.1	73.7	right	Lobule VIIa crusI (Hem) Cerebelum (Crus 1)	10.4	3.69	14	-86	-28
	80.8	17.6	right	Lobule VI (Hem) Cerebelum (VI)	4.5	3.64	40	-60	-26
	20.5	4.5	right	Lobule VIIa crusII (Hem)	1.4				
	14.6	3.2	right	Area FG2	4.5				
	1.5	0.3	right	Area hOc3v [V3v]	0.2				
	1	0.2	right	Area FG1	0.4				
	0.5	0.1	right	Area hOc2 [V2]	0				
	0.1	0	right	Area FG3	0				
	0.1	0	right	Lobule VI (Verm)	0.1				
	0.1	0	right	Area hOc4v [V4(v)]	0				

Supplementary Table 16. BOLD activity covarying positively with -EVs after removing variance explained by *wf*.
Only clusters surviving a 5% FWE correction at the cluster size are reported (*t*=2.5. *p*<.01, cluster size 420; resampled voxel size: 2x2x2mm; Figure 18b). Brain regions are identified using the Anatomy Toolbox[30]. The columns refer to the size in voxels of each cluster; the number of voxels of that cluster falling within a specific cytoarchitectonic region; the percentage of voxels in that region; hemisphere; cytoarchitectonic region (if available) or macro-anatomical description of the region; percentage of cytoarchitectonic region activated by cluster; peak t-value within a particular region; and MNI coordinates of the peak. If more peaks were identified within the same cyto-architectonic or anatomical region only the peak with the highest t-value was included in the table. Peaks falling outside the gray matter are not included in the table. Cyto architectonic description is only reported when a voxel has a probability over 40% to fall in that area and only for cyto-architectonic areas available in the anatomy toolbox; anatomical description is otherwise reported. The '*.txt' file generated by the Anatomy toolbox can be found at: https://osf.io/rk8w4/?view_only=98b193a58aff48dda40b9d3d91ac5254

Supplementary Note 17. PE_M and PE_S separability

The average correlation between the time courses of the parametric modulators for PE_S and PE_M was -0.26, ranging from -0.49 to -0.03. Due to this correlation, we explored whether our experimental design and GLM approach can disentangle voxels that represent PE_S from those representing PE_M, and whether they can differentiate voxels linearly dependent on *wf* from those that are not. Our GLM included, during the outcome period, a boxcar for the duration of the movie with two parametric modulators, one for PE_S and one for PE_M. Both have been normalized by dividing them with 1-*wf* and *wf* respectively. This was done, as described in the Methods and Materials section of the main manuscript, to ensure that PE_S and PE_M predictors become independent of preference and *wf* per se. When used in the GLM, the parameter estimates for these normalized PE_S values can then be compared across participants to identify if the brains of participants with higher weight on shocks (i.e. larger value for 1-*wf*) show larger signals for a given outcome than participants with lower weight on shocks. Using the original PE_S values would make that interpretation difficult, because they are already dependent on *wf*.

For this parameter recovery, we ran 1000 simulations. In each, we simulated 25 participants. For each participant, we used their own design matrices (the same used for the actual GLM first level analysis of the fMRI activity after convolution with the haemodynamic response function) to mix signals in each subject using three mixings (i) $-1*PE_S+0*PE_M$+noise; (ii) $0*PE_S+1*PE_M$+noise, and (iii) $-1*PE_S+1*PE_M$+noise. Noise was a random gaussian set at 1std of the mixed signal. Next, we ran a GLM using the same design matrix, and saved the parameter estimates for PE_S (we will call βPE_S) and PE_M (we will call βPEM) for each participant. We then perform a t-test for βPE_S and one for βPE_M to see if across the 25 parameter estimates (one per participant) there is evidence against the null hypothesis $H0{:}\beta PE_S=0$ or $H0{:}\beta PE_M=0$. Of course, if PE_S was mixed into the voxels activity (case i or iii), a significant t-test would be a hit, while a non-significant t-test would be a miss, and the same applies to PE_M for case ii. After repeating this procedure 1000 times, we count the proportion of the 1000 simulations where a t-test was significant against $H0{:}\beta PE_S=0$ or $\beta PE_M=0$. Additionally, to see how often the analysis falsely detects a dependence on *wf* although *wf* was not included in the mixing, we also look at $r(wf, \beta PE_S)=0$ and $r(wf, \beta PE_M)=0$. Initially, we use $p<0.05$ as a criterion, to look at the specificity and sensitivity for the case in which we explore responses in the AVPS, which is univariate. We also indicate proportions at $p<0.001$, but this time for a one-tailed test, in parenthesis, to provide results relevant for an explorative whole brain analysis where the cluster-cutting threshold was set at 0.001. The proportion of significant results was as follows:

**H_0 mixing**	**$-1·PE_S+0·PE_M$**	**$0·PE_S+1·PE_M$**	**$-1·PE_S+1·PE_M$**
**$\beta PE_S=0$**	99.8(92.4)	4.4(0)	99.8(90.6)
**$\beta PE_M=0$**	5.5(0.1)	99.8(90.8)	99.5(91.9)
**$r(wf, \beta PE_S)=0$**	5.2(0.7)	5.3(0.1)	5.4(0.4)
**$r(wf, \beta PE_M)=0$**	4.5(0.2)	5.1(0.1)	3.8(0.0)

Supplementary Table 17. Frequentist tests for simulations without *wf* dependence.
Percentage of two-tailed *t*-tests significant at $p<0.05$ from the 1000 simulations using signals generated without multiplications with *wf* or (1-*wf*), and in brackets, the percentage of one tailed $p_{1\text{-tailed}}<0.001$. The top row specifies how the signals were generated before adding 1sd of noise, the leftmost column, the null hypothesis that was tested in the hypothesis testing.

We then repeated the same analysis, but this time multiplying the signals with (1-*wf*) and *wf* as indicated in Supplementary Table 13 to simulate cases of voxels where signal strength depends on preference.

H_0 mixing	$-1 \cdot (1-wf) \cdot PE_S + 0 \cdot wf \cdot PE_M$	$0 \cdot (1-wf) \cdot PE_S + 1 \cdot wf \cdot PE_M$	$-1 \cdot (1-wf) \cdot PE_S + 1 \cdot wf \cdot PE_M$
**$\beta PE_S=0$**	99.3(81.1)	3.5(0.3)	99(80.5)
**$\beta PE_M=0$**	3.8(0.2)	98.5(64.8)	98.7(62.3)
**$r(wf, \beta PE_S)=0$**	75.9(26.4)	5.1(0.0)	74.6(26.5)
**$r(wf, \beta PE_M)=0$**	5(0.0)	98.3(77.3)	98.5(79.3)

Supplementary Table 18. Frequentist tests with or without *wf* dependence.
Percentage of two-tailed t-tests significant at $p<0.05$ from the 1000 simulations using signals generated with multiplications with *wf* or (1-*wf*), and in brackets, the percentage of $p_{1\text{-tailed}}<0.001$. The top row specifies how the signals were generated before adding 1sd of noise, the leftmost column, the null hypothesis that was tested in the hypothesis testing.

The above tables explore evidence against the null hypothesis, but for univariate analysis we also ask whether we can actually provide evidence for voxels mixed without a certain factor that the GLM provides evidence for the null hypothesis using Bayesian statistics [57], using a bound of $BF_{10}<1/3$. Using a Bayesian test, with n=25, we know that $|t|<1$ provides evidence in favour of H0: $\beta PE_S=0$ over H1: $\beta PE_S \neq 0$, and $|r|<0.17$ for H0: $r(wf, \beta PE_S)=0$ over H1: $r(wf, \beta PE_S) \neq 0$ ($BF_{10}<1/3$, using default priors in JASP). We thus counted the proportion with evidence in favour of H0 over H1 in all cases using these bounds.

H_0 mixing	$-1 \cdot PE_S + 0 \cdot PE_M$	$0 \cdot PE_S + 1 \cdot PE_M$	$-1 \cdot PE_S + 1 \cdot PE_M$
**$\beta PE_S=0$**	0	69.6	0
**$\beta PE_M=0$**	66	0	0
**$r(wf, \beta PE_S)=0$**	57.1	58.5	59.3
**$r(wf, \beta PE_M)=0$**	58.0	56.8	58.4

Supplementary Table 19. Bayesian tests for simulations without *wf* dependence
Percentage of $BF_{10}<1/3$ from the 1000 simulations using signals generated without multiplications with *wf* or (1-*wf*). The top row specifies how the signals were generated before adding 1sd of noise, the leftmost column, the null hypothesis that was tested in the hypothesis testing.

H_0 mixing	$-1 \cdot (1-wf) \cdot PE_S + 0 \cdot wf \cdot PE_M$	$0 \cdot (1-wf) \cdot PE_S + 1 \cdot wf \cdot PE_M$	$-1 \cdot (1-wf) \cdot PE_S + 1 \cdot wf \cdot PE_M$
**$\beta PE_S=0$**	0	70.1	0
**$\beta PE_M=0$**	68.1	0.1	0
**$r(wf, \beta PE_S)=0$**	2.0	57.5	3.6
**$r(wf, \beta PE_M)=0$**	57.5	0.0	0.0

Supplementary Table 20. Bayesian tests with or without *wf* dependence.
Percentage of $BF_{10}<1/3$ from the 1000 simulations using signals generated with multiplications with *wf* or (1-*wf*). The top row specifies how the signals were generated before adding 1sd of noise, the leftmost column, the null hypothesis that was tested in the hypothesis testing.

Summary: In our simulations, with 1sd of noise, we can detect voxels with signals linearly dependent on PE_S and/or PE_M accurately: If we use alpha=0.05, as we would for the AVPS analysis, signals generated by

including PE_S but not PE_M are detected as representing PE_S in ~99% of cases, and only in ~5% of cases as representing PE_M, and vice versa for voxels generated to include PE_M but not PE_S. Using Bayesian statistics, we can even provide evidence in favor of the H0 for the former ($\beta PE_S=0$) in ~70% of cases, and the latter H0 ($\beta PE_M=0$) in 66% of cases. Within our sample size, we thus have power to arrive at conclusions that match the way we generated the signals in the majority of simulations. Even when $p<0.001$ is used, as it would for our exploratory whole brain analysis, power remains decent.

With regard to linear dependence on *wf*, we find that for voxels generated with PE_S but not PE_M signals, if the signal was generated using (1-*wf*) as a multiplier, a significant correlation is detected in 76% of cases, and when not used in the generation, a significant correlation is found in 5% of cases, while evidence for H0:r(*wf*, βPE_S)=0 is found 57% of cases.

Supplementary Note 18. Two-steps procedure

In learning paradigms like the one we use, that PE=Out-EV causes outcomes (Out) and predictor errors (PE) to be highly correlated, as a positive Out trial (high-money or low-shock) will always be associated with positive PE, and negative Out trial with negative PE (see the formulae connecting Out and PE in Figure 5a). In our fMRI study, PE_S and Out_S had an average correlation of 0.741 (ranging 0.750 to 0.866 across our 27 participants), and the PE_M and Out_M had an average correlation of 0.749 (ranging 0.781 to 0.862 across our 27 participants). To disentangle whether a region or network with signals correlating with PE really encodes PE or simply encodes Out, Zhang and colleagues (2020) proposed a simple analysis: if the signal correlates with Out but not EV, they propose to consider it to encode Out not PE; if the signal correlates with Out and, in the opposite direction, with EV, they propose to consider it to encode PE[39]. For the signals in our two signatures and in the frontal clusters associated with PE_S we applied this logic. We extracted raw blood-oxygen-level dependent (BOLD) time series from the two signatures (i.e. by multiplying each volume with the signatures to create a scalar time-series for each signature) and the average signal from the two prefrontal ROIs that had signals correlating with PE_S (yellow in Figure 7b). These time series of each participant were then time-locked to 2s before onset of the video clip to 12s after the end of the video. Time series were up-sampled to a resolution of 200 ms using 2D cubic-spline interpolation, resulting in a data matrix of size m-by-n, where m is the number of trials, and n is the number of the up-sampled time points (i.e., 14s/200ms = 70 time points). A multiple regression model containing Out_S, Out_M, EV_S and EV_M was then estimated at each time point (across trials) for each participant. It should be noted that, although the linear regression here took a similar formulation as the first-level general linear model (GLM), it did not model any specific onset; instead, this regression was fitted at each time point in the entire trial across all the trials.

To test group-level significance of the above time series analysis, we employed a permutation procedure. We defined a time window of 4–8 s after the corresponding event onset, during which the BOLD response was expected to peak. In this time window, we randomly flipped the signs of the time courses of effect sizes for 5,000 repetitions to generate a null distribution, and tested whether the mean of the generated data from the permutation procedure was smaller or larger than 97.5% of the mean of the empirical data.

For the AVPS, we found an expected negative correlation with OutS (i.e. higher AVPS signals for high-shock than low-shock trials, which is what the signature was designed to do[22]) and a positive correlation with EV_S. To our knowledge, this may be the first evidence that the facial pain-witnessing network that had only been developed to discriminate the sight of high- vs low-pain facial expressions actually encodes predictor errors in a learning context - at least in our paradigm[22].

For the RS, we found the expected positive correlation with positive outcomes (i.e. with Out_S and Out_M) and a negative correlation with EV_S and EV_M. In agreement with a substantial literature associating this striato-prefrontal network with predictor error coding[11,23], we therefore may conclude that it encodes PE in our paradigm, both for Shocks and Money.

For the two prefrontal regions emerging from our PE_S analysis after removing variance explained by *wf*, we find both to show the pattern that would be expected if they encoded PE_S: correlating positively with OutS and negatively with EVS. Somewhat less surprisingly, both also seem to show some positive correlation with EV_M, in line with the notion that the vmPFC could generate a common currency combining the values in terms of Shocks and Money to later enable a decision that combines these two measures[23,24].

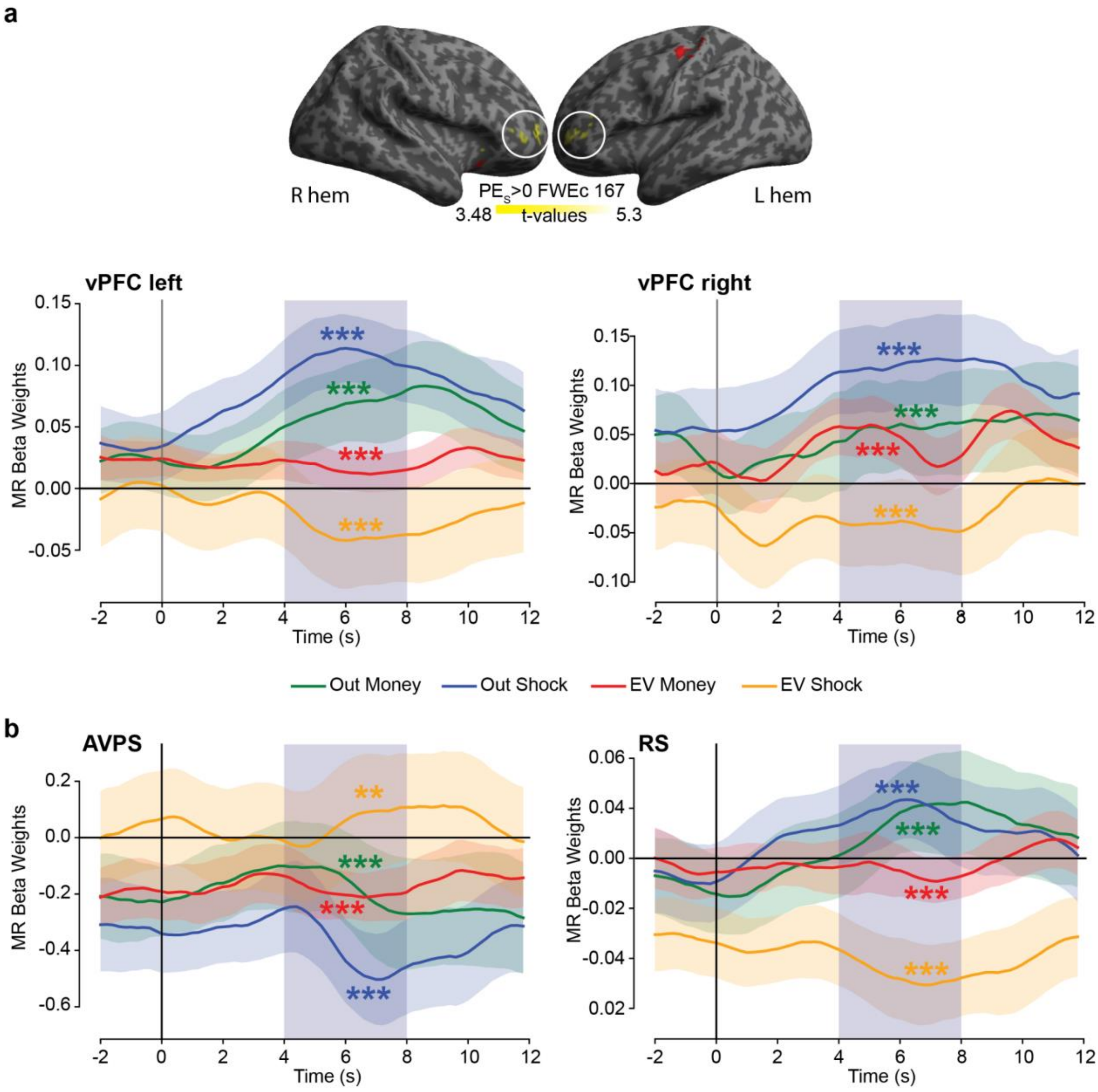

Supplementary Fig. 19: Dissociating Outcome vs Prediction Error coding.
a Results of a multiple regression analysis performed within the two ventral prefrontal cortex clusters of Figure 7b (left and right vPFC) that showed significant PE_S correlation after removing variance explained by *wf* (PES>0, one-tail, $p_{unc}<0.001$, $t=3.48$, FWEc 167). Beta-weights that are significantly different from zero based on permutation statistics in the time interval indicated by the gray box are marked by stars (**: $p<0.01$, ***: $p<0.001$). Shading indicates the s.e.m. across participants. Time=0 indicates the onset of the movie showing the outcome of a trial. The location of the clusters is shown in yellow in the renders. **b** Same for the AVPS and RS neural signatures. Out_M in green, Out_S in dark blue, EV_M in red and EV_S in yellow. Source data are provided as a Source Data file.

Supplementary Note 19. Psychological description of the difference between our learning models

All our learning models had in common that they learned by updating expected values for the two symbols using prediction errors, and additively combined self-money and other-shock. All of them captured individual variability in preference using a weighting factor (*wf*). What varied was when the two outcomes were combined. In M1, the outcomes themselves are combined into a single composite outcome value, which is already biased by the participants preference (Figure 5a). Translated in psychological terms, this model would capture a learning in which each symbol is associated with a value that captures how good or bad the outcomes for those symbols have felt in the past. In case of devaluation, a learner using this algorithm wouldn't switch their preference to the symbol that has the highest expected value on the remaining quantity because they wouldn't have an internal model that separates expectations for self-money and other-shock. In contrast, in our unbiased and separable version (M2Dec), participants track expectations separately for self-money and other-shock - independently of preference - and preference only plays out during the decision-phase. In psychological terms, this captures a form of learning in which participants separately know how likely each symbol will lead to self-money or other-shock, respectively. Only when a decision must be taken, will they combine these predictions with the relative value that self-money and other-shocks have for them personally in this specific context, to come to a decision under conflict (Figure 5b). In this version, the variable 'expected value' represents expectation in the objective units in which the outcome is coded, independently of whether this particular outcome is more or less valued by the participant in this specific conflict situation. Accordingly, in case of devaluation, the participant can base their decision on accurate predictions for the remaining quantity, and should have ~80% preference for the symbol with the highest expected value for the remaining quantity. Finally, in our biased but separable version (M2Out), participants also track separate expectations for both symbols in terms of self-money and other-shock, but they do so in ways that depend on their individual preference. Specifically, when self-money and other-shock outcomes are revealed, they are multiplied with their subjective weight (*wf* for self-money and *1-wf* for other-shock), and expectations are updated using these weighted values. At the decision-stage, the comparison between the two symbols is then done based on a simple sum of these already weighted expected values. Psychologically, this means that people have separate models for self-money and other-shock, but that the models predict the subjective value of each choice rather than the objective outcomes they are associated with. As a result, in case of devaluation, participants will have weaker preferences for the symbol that leads to more favorable outcomes if the outcome that remains was the outcome they valued less, than if the outcome that remained was the outcome they valued more.

Supplementary Note 20. Stress Tolerance short questionnaire (STSQ), and association with *wf*

The Stress Tolerance Short Questionnaire is designed to measure the stress tolerance quotient and the participants' anxiety level.
In the first part participants have to rate each item from 1 (always) to 5 (never), according to how much of the time the statement is true for them. In the second part participants have to rate each item from 0 (not at all sure) to 3 (nearly every day), according to how often they have been bothered by the following problems over the last 2 weeks.

**Please, rate each items from 1 (always) to 5 (never), according to how much of the time the statement applies to you**					
	Always	Frequently	Neutral	Sometimes	Never
1. I eat at least one balanced meal a day.	1	2	3	4	5
2. I exercise to the point of perspiration at least twice a week.	1	2	3	4	5
3. I limit myself to less than half a pack of cigarettes a day	1	2	3	4	5
4. I take fewer than five cups of tea a week.	1	2	3	4	5
5. I regularly attend social activities.	1	2	3	4	5
6. I am in good health (including eye-sight, hearing, teeth).	1	2	3	4	5
7. do something for fun at least once a week.	1	2	3	4	5
8. I am able to organize my time effectively.	1	2	3	4	5
9. I drink fewer than three cups of coffee a day.	1	2	3	4	5
10. I take some quite time for myself during the day.	1	2	3	4	5
**Over the last 2 weeks, how often have you been bothered by the following problems?**					
		Not at all sure	Several days	Over half the days	Nearly every day
11. Feeling nervous, anxious, or on edge		0	1	2	3
12. Not being able to stop or control worrying		0	1	2	3

13. Becoming easily annoyed or irritable	0	1	2	3
14. Feeling afraid as if something awful might happen	0	1	2	3

Supplementary Table 21. Stress Tolerance Short Questionnaire.

If participants obtained a score higher than 50 in the STSQ we would advise them not to continue with the experiment. This is because during the experiment they would be confronted with a number of possibly stressful videos, and put in a position in which their own benefit is detrimental to another individual. However, in case participants obtained a score higher than 50, we still gave them the possibility to continue with the task, after witnessing a sample video displaying a high intensity electrical stimulation to the confederate. None of our participants obtained a score higher than 50 in the STSQ.

A correlation between the STSQ score and *wf* remains inconclusive (Kendall's Tau=-0.124; BF_{10}=0.529; p=0.113)

Supplementary Note 21. Pain rating, pre- and post-disclosure feedback questionnaires and associations with *wf*

Soon after the learning task, participants were shown two randomly chosen videos from the pool used during the learning task (“Short pain rating” in Supplementary Fig. 1). One was an example from the no-shock outcome and one was from the shock outcome. Participants were asked to rate how much pain they thought the person in the video felt (“How much pain do you think the person felt?”), using a scale from 0 to 10 in which 0=no pain: no sensation on the hand and 10=excruciating painful sensation. We then correlated the difference between the rating for the painful shock and non-painful shock videos with *wf*, and found evidence against the existence of such a correlation: Kendall’s tau=0.010, BF_{10}=0.149, *p*=0.906. The same is true when correlating *wf* with the difference in reaction time between rating the painful shock and non-painful shock videos: Kendall’s tau=0.018, BF_{10}=0.152, *p*=0.812.

At the end of the experiment we collected two feedback questionnaires, one before disclosing the cover story (“Participant feedback questionnaire” in Supplementary Fig. 1) and one after (“Post disclosure feedback questionnaire” in Supplementary Fig. 1). As the two feedback questionnaires do not produce a single summary score, we ran two linear regression with *wf* as dependent variable and all the answers to the questionnaires as covariates (forward method, note that QQ plot confirms that the requirements for a multiple regression are met). For the feedback questionnaire prior to disclosing the cover story, the linear regression revealed that only Q4 and Q8 contribute significantly to explaining *wf* (Supplementary Fig. 20, ANOVA containing intercept, Q4 and Q8, $F_{(2,76)}$=10.948, *p*=6.636*10-5, BF_{10}=360 relative to intercept only). For the feedback questionnaire after disclosure, the first 3 questions relative to whether participants had believed the cover story are analyzed and reported in Supplementary Note §5. The remaining 8 questions (Q4-Q11) were entered into a multiple regression that was not outperforming an intercept only model ($F_{(8,70)}$=0.693, *p*=0.697. All BF_{incl}<0.150).

**Participant feedback questionnaire** (before disclosing the cover story)							
**Please indicate how much you agree with the following statements**	**strongly disagree**	**disagree**	**Somewhat disagree**	**Neither agree or disagree**	**Somewhat agree**	**agree**	**totally agree**
1. I felt uncomfortable when I saw the person in the videos getting the painful stimulation							
2. I am concerned about the welfare of the person who will receive the electrical stimulations							
3. I very quickly understood the different outcomes associated with each symbol							
4. Once I discovered the outcome-symbol association, I voluntarily chose the most financially rewarding symbol for myself							
5. I always felt guilty when I saw a video depicting a high intensity electrical stimulation							

6. I felt responsible for my choices							
7. I felt uncomfortable when not knowing the consequences of each symbol							
8. Before the experiment started I felt distressed							
9. During the experiment I felt distressed because of the conflictual situation							
10. I now feel distressed because of the experience							
11. It was easy to understand the task							
12. I was motivated throughout the task							
13. I participated to earn money							
14. I participated to contribute to scientific knowledge							
15. I participated out of curiosity							
16. I felt I could help the person who will receive the electrical stimulations by choosing one symbol instead of the other							
17. I feel that conflicts of this type are part of my daily life							

Supplementary Table 22. Pre-disclosure feedback questionnaire.

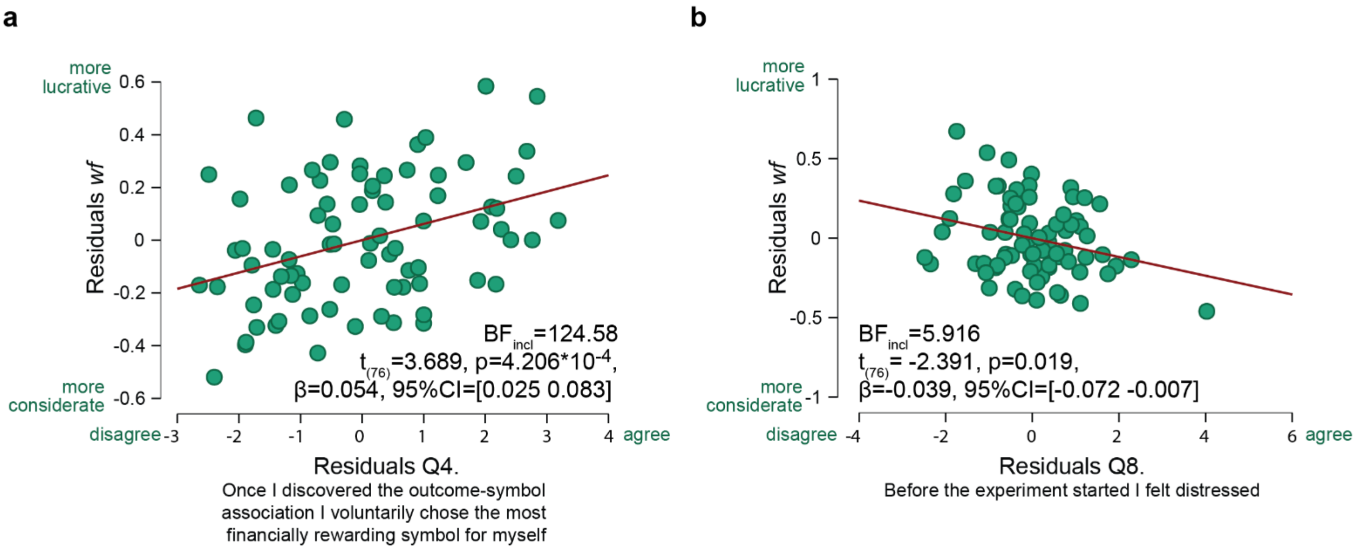

Supplementary Fig. 20: Multiple regression explaining *wf* using Q4 and Q8 of the pre-disclosure feedback questionnaire. **a** Regression explaining the residuals of *wf* given the residuals of Question 4 of the Feedback questionnaire. **b** Regression explaining the residuals of *wf* given the residuals of Question 8 of the Feedback questionnaire. Residuals are plotted because these are the results of a multiple regression including Q4 and Q8, and the figure thus shows the unique variance explained by each variable after removing what can be explained by the others. *T* and *p* refer to the parameter estimate for that particular variable, BF_{incl}, how much more likely the *wf* data is given a model including that variable compared to one excluding this variable. All tests are two-tailed. Source data are provided as a Source Data file.

Post disclosure feedback questionnaire (after disclosing the cover story)

1. Before choosing whether or not to see the other person receiving the electrical stimulations, I believed that someone was really going to receive them.

Strongly disagree
Disagree
Somewhat disagree
Neither agree nor disagree
Somewhat agree
Agree
Totally agree

2. In case you didn't believe it, what made you suspicious about the cover story?

Open field

3. In case you didn't believe the cover story, did you run the task as if you believed that someone would receive part of the electrical stimulations at the end of the task?

Yes
No

4. I am upset for having been lied to regarding the other participant.

Strongly disagree
Disagree
Somewhat disagree
Neither agree nor disagree
Somewhat agree
Agree
Totally agree

5. I feel relieved to know that another person was not hurt during the experiment.

Strongly disagree
Disagree
Somewhat disagree
Neither agree nor disagree
Somewhat agree
Agree
Totally agree

6. I find that the distress I felt during the experiment is acceptable given how important it is to understand how we learn how actions influence the welfare of others (if you were never distressed, please select "Not Distressed").

Strongly disagree
Disagree
Somewhat disagree
Neither agree nor disagree
Somewhat agree
Agree
Totally agree
Not distressed

7. The facial expressions in the learning task realistically expressed pain.

Strongly disagree
Disagree
Somewhat disagree
Neither agree nor disagree
Somewhat agree
Agree
Totally agree

8. I understand the reasons for the deception used in the study.

Strongly disagree
Disagree
Somewhat disagree
Neither agree nor disagree
Somewhat agree
Agree
Totally agree

9. I feel upset about the study.

Strongly disagree
Disagree
Somewhat disagree
Neither agree nor disagree
Somewhat agree
Agree
Totally agree

10. I would recommend the study to a friend.

Strongly disagree
Disagree
Somewhat disagree
Neither agree nor disagree
Somewhat agree
Agree
Totally agree

11. I feel angry about the study.

Strongly disagree
Disagree
Somewhat disagree
Neither agree nor disagree
Somewhat agree
Agree
Totally agree

12. Have you participated in study with deception before?

Yes
No

If you answered yes, what was the purpose of the study in question?

Open field

Supplementary Table 23. Post-disclosure feedback questionnaire.

Supplementary Note 22. Eye gaze analysis

In an independent group of participants (N=41) we tested whether participants maximizing self-money would look away from the facial expressions of pain (compared to participants minimizing the pain to others), to reduce their moral conflict. We collected eye gaze data through Gorilla.sc. Specifically, we correlated the percentage of time spent by participants fixating the facial expression of pain videos in the Conflict condition, with the percent of considerate choices they made. As the percentage of fixation on the videos was not normally distributed (Shapiro-Wilk= 0.914, p=0.004) we calculated the Kendall's $\boldsymbol{\tau}$. Supplementary Fig. 8 shows the results of this correlation: despite a tendency in the expected direction, the relation was not significant and the Bayes factor leaned in favour of the null hypotheses. This preliminary data speaks against the idea that participants with more lucrative preference strongly avoided to look at the facial expressions of pain compared to participants with more considerate preference. This also dovetails with the observation that activity in the vicarious affective pain signature did not depend on *wf*.

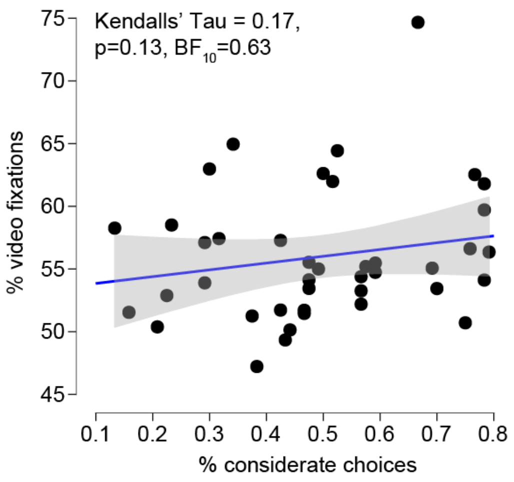

Supplementary Fig. 21. Correlation between time spent looking at the video and considerate choices in the Conflict condition. Blu line and gray shading represent the regression line and the 95% confidence interval. N=41. Two-tail testing was used. Source data are provided as a Source Data file.
