# Supplementary material for: Neuro-computational mechanisms and individual biases in action-outcome learning under moral conflict
Source: Nat Commun. 2023 Mar 6;14:1218. doi: 10.1038/s41467-023-36807-3 (PMC9988878; doi:10.1038/s41467-023-36807-3)
Supplement: Supplementary file 3 — Reporting Summary [file 41467_2023_36807_MOESM3_ESM.pdf]

nature portfolio

Corresponding author(s): Valeria Gazzola

Last updated by author(s): Oct 18, 2022

# Reporting Summary

Nature Portfolio wishes to improve the reproducibility of the work that we publish. This form provides structure for consistency and transparency in reporting. For further information on Nature Portfolio policies, see our Editorial Policies and the Editorial Policy Checklist.

## Statistics

For all statistical analyses, confirm that the following items are present in the figure legend, table legend, main text, or Methods section.

| n/a | Confirmed | |
|---|---|---|
| ☐ | ☒ | The exact sample size (*n*) for each experimental group/condition, given as a discrete number and unit of measurement |
| ☐ | ☒ | A statement on whether measurements were taken from distinct samples or whether the same sample was measured repeatedly |
| ☐ | ☒ | The statistical test(s) used AND whether they are one- or two-sided<br>*Only common tests should be described solely by name; describe more complex techniques in the Methods section.* |
| ☐ | ☒ | A description of all covariates tested |
| ☐ | ☒ | A description of any assumptions or corrections, such as tests of normality and adjustment for multiple comparisons |
| ☐ | ☒ | A full description of the statistical parameters including central tendency (e.g. means) or other basic estimates (e.g. regression coefficient) AND variation (e.g. standard deviation) or associated estimates of uncertainty (e.g. confidence intervals) |
| ☐ | ☒ | For null hypothesis testing, the test statistic (e.g. *F*, *t*, *r*) with confidence intervals, effect sizes, degrees of freedom and *P* value noted<br>*Give P values as exact values whenever suitable.* |
| ☐ | ☒ | For Bayesian analysis, information on the choice of priors and Markov chain Monte Carlo settings |
| ☐ | ☒ | For hierarchical and complex designs, identification of the appropriate level for tests and full reporting of outcomes |
| ☐ | ☒ | Estimates of effect sizes (e.g. Cohen's *d*, Pearson's *r*), indicating how they were calculated |

*Our web collection on statistics for biologists contains articles on many of the points above.*

## Software and code

Policy information about availability of computer code

| | |
|---|---|
| Data collection | Online platform Gorilla (https://gorilla.sc/) for the online experiment, and Presentation for the fMRI experiment. |
| Data analysis | Quantification of choices was performed in RStudio (Version 1.1.453) and Matlab (https://nl.mathworks.com/ Version 2021b). Statistical analyses were then performed using JASP (https://jasp-stats.org, version 0.11.1), to provide both Bayes factors and p values.<br>Models were fitted in RStan (version 2.18.2, http://mc-stan.org/rstan/) using a hierarchical Bayesian approach, i.e. by estimating the actual posterior distribution through Bayes rule. Our models were adapted from the R package hBayesDM (for “hierarchical Bayesian modeling of Decision-Making tasks”, version 1.2) described in detail in Ahn et al (2017) (52).<br>All data and analysis code are available at osf.io DOI 10.17605/OSF.IO/RK8W4<br>MRI data were processed in SPM12 (55) |

For manuscripts utilizing custom algorithms or software that are central to the research but not yet described in published literature, software must be made available to editors and reviewers. We strongly encourage code deposition in a community repository (e.g. GitHub). See the Nature Portfolio guidelines for submitting code & software for further information.

## Data

Policy information about availability of data

All manuscripts must include a data availability statement. This statement should provide the following information, where applicable:

- Accession codes, unique identifiers, or web links for publicly available datasets
- A description of any restrictions on data availability
- For clinical datasets or third party data, please ensure that the statement adheres to our policy

The behavioral choice data generated in this study have been deposited in OSF.io under DOI https://doi.org/10.17605/OSF.IO/RK8W4. The raw fMRI data are protected and are not available due to data privacy laws. The processed fMRI data are available at OSF.io under DOI https://doi.org/10.17605/OSF.IO/RK8W4. The data directly illustrated in the figures are provided in the Supplementary Information/Source Data file.

# Field-specific reporting

Please select the one below that is the best fit for your research. If you are not sure, read the appropriate sections before making your selection.

☒ Life sciences ☐ Behavioural & social sciences ☐ Ecological, evolutionary & environmental sciences

For a reference copy of the document with all sections, see nature.com/documents/nr-reporting-summary-flat.pdf

# Life sciences study design

All studies must disclose on these points even when the disclosure is negative.

| | |
|---|---|
| Sample size | In total, 106 healthy volunteers with normal or corrected-to-normal vision, and no history of neurological, psychiatric, or other medical problems, or any contraindication to fMRI (for the fMRI experiment only) were recruited for our experiments (Table 1). Particularly, 27 participants (37y±17SD; 27f) took part in the fMRI, and 79 (25y±7SD, 39f) in the Online experiment, with the two samples being independent. Sample sizes were chosen based on a pilot study that suggested a three tier distribution of preferences, and hence the online study was chosen to have over 20 participants per sample. Because Bayesian analysis supplement traditional frequentist approaches, no poweranalysis was performed beforehand. For the fMRI study, we used a sample size that is typical for this kind of studies. |
| Data exclusions | As two of the 27 participants in the fMRI were left handed, and stimuli showed movements of the right hand of the actor, to reduce variability possibly induced by lateralization of the brain responses, these two participants only performed the tasks off-line (i.e. no fMRI data acquired). |
| Replication | The online data was similar to a smaller pilot study, and therefore replicated once. The fMRI study was not replicated yet in terms of brain activity (due to access limitations due to the COVID pandemic), but the choice behavior of the fMRI participants is similar to the purely behavioral studies we have performed. |
| Randomization | The designs are within participant, and the order of conditions was randomized. |
| Blinding | All participants were subject to the same conditions, and blinding participants as to the condition they are in is therefore irrelevant. |

# Reporting for specific materials, systems and methods

We require information from authors about some types of materials, experimental systems and methods used in many studies. Here, indicate whether each material, system or method listed is relevant to your study. If you are not sure if a list item applies to your research, read the appropriate section before selecting a response.

## Materials & experimental systems

| n/a | Involved in the study | |
|---|---|---|
| ☒ | ☐ | Antibodies |
| ☒ | ☐ | Eukaryotic cell lines |
| ☒ | ☐ | Palaeontology and archaeology |
| ☒ | ☐ | Animals and other organisms |
| ☐ | ☒ | Human research participants |
| ☒ | ☐ | Clinical data |
| ☒ | ☐ | Dual use research of concern |

## Methods

| n/a | Involved in the study | |
|---|---|---|
| ☒ | ☐ | ChIP-seq |
| ☒ | ☐ | Flow cytometry |
| ☐ | ☒ | MRI-based neuroimaging |

# Human research participants

Policy information about studies involving human research participants

| | |
|---|---|
| Population characteristics | See Table 1 in the paper. Online Exp n=79, age 25±7, 39females 40 males. fMRI experiment: n=27, age 37±17, 27 females. |
| Recruitment | Participants for the online task were recruited through Prolific (https://prolific.ac/), while those for the fMRI through advertisements of the experiment on social media. Participation in Prolific is sometimes financially motivated, which might have led to an over-representation of lucrative preferences. Participation in the fMRI study required additional time and effort investment, which may have led to an over-representation of more prosocial preferences. All participants provided informed consent. |
| Ethics oversight | The studies were approved by the Ethics Committee of the University of Amsterdam, The Netherlands (2017-EXT-8201, 2018-EXT-8864, 2020-EXT-12450). |

Note that full information on the approval of the study protocol must also be provided in the manuscript.

# Magnetic resonance imaging

## Experimental design

| | |
|---|---|
| Design type | task, event related |
| Design specifications | each trial started with a jittered fixation cross lasting 3-9 seconds. Then the two symbols appeared and participants could make their choice without a time restriction. After the button press, there was a fixation cross raging from 3-9 seconds and the video with a duration of 2 seconds followed. There were a total of 60 trials per participants organized in a single run. |
| Behavioral performance measures | Binary choices between two symbols on 60 trials. |

## Acquisition

| | |
|---|---|
| Imaging type(s) | functional |
| Field strength | 3T |
| Sequence & imaging parameters | MRI images were acquired with a 3-Tesla Philips Ingenia CX system using a 32-channel head coil. One T1-weighted structural image (matrix = 240x222; 170 slices; voxel size = 1x1x1mm) was collected per participant together with an average of 775.83 EPI volumes ± 23.11 SD (matrix M x P: 80 x 78; 32 transversal slices acquired in ascending order; TR = 1.7 seconds; TE = 27.6ms; flip angle: 72.90°; voxel size = 3x3x3mm, including a .349mm slice gap). |
| Area of acquisition | Whole brain |

Diffusion MRI ☐ Used ☒ Not used

## Preprocessing

| | |
|---|---|
| Preprocessing software | SPM12 |
| Normalization | MRI data were processed in SPM12 (55). EPI images were slice-time corrected to the middle slice and realigned to the mean EPI. High quality T1 images were coregistered to the mean EPI image and segmented. The normalization parameters computed during the segmentation were used to normalize the gray matter segment (1mmx1mmx1mm) and the EPIs (2mmx2mmx2mm) to the MNI templates. Finally, EPIs images were smoothed with a 6mm kernel. |
| Normalization template | MNI |
| Noise and artifact removal | Motion regressors |
| Volume censoring | *Define your software and/or method and criteria for volume censoring, and state the extent of such censoring.* |

## Statistical modeling & inference

| | |
|---|---|
| Model type and settings | The design matrix to analyze the fMRI data of the learning task included 13 regressors: (1) A decision regressor starting with the appearance of the two symbols and ended with the button press of the participant. (2) The outcome regressor was aligned with the presentation of the video and had a fixed duration of 2 seconds, corresponding to the duration of the stimulus. (3) A button-press regressor with zero duration was aligned to the moment of button-pressing. (4-5) The decision regressor had 2 parametric modulators (EVM and EVS of the chosen option); and (6-7) the outcome regressor 2 parametric modulators (PES and PEM). The modulators were derived from the winning M2Out, then mean- subtracted and wf-normalized before being entered into the design matrix (see wf-normalization below and Supplementary Materials §15). (8-13) Finally, 6 regressors of no interest were included to model head translations and rotations.<br>Which parametric modulator to include was based on the results of parameter correlations and recovery. Prediction errors and actual outcomes could not be used within the same GLM as they were too highly correlated (r(PES,OutS)=0.741, ranging |

from 0.750 to 0.866 across our 27 participants; r(PEM,OutM=0.749, ranging from 0.781 to 0.862). We however examined if signals were associated with Out vs PE using the method suggested by Zhang and colleagues(Zhang et al., 2020). During the outcome phase, we therefore only included PES and PEM, which are only weakly correlated (average r=-0.26, ranging from -0.49 to -0.03 across the participants) and for which parameter recovery is robust (Supplementary Materials §17).

wf-normalization: Because we are interested in whether PES or PEM representations depend on wf or not, we divided PES with (1-wf) and PEM with wf before entering them into the design matrix. As a result, the first PES value in the parametric modulator would always be PES=-1 if it was a high-shock outcome or PES=+1 for a low-shock outcome, independently of the participant's wf value. If signals covary with PES in a way that does not linearly depend on wf, the parameter estimate across participants (βPES) would violate H0:βPES=0, but not H0:r(βPES=0,wf)=0. If signals covary with PES in a way that does linearly depend on wf, it would violate both of these H0. Note that for outcomes, the coding was +1 for good outcomes (i.e. high money or low shock) and -1 for bad outcomes (i.e. low money or high shock). EV and PE follow that polarity. The same was applied to EVS and EVM, which were divided by (1-wf) and wf, respectively. This approach is illustrated with an example in Supplementary Materials §15.

Results were then analyzed in two ways. First, to improve reverse inference, we used two multivariate signatures the affective vicarious pain signature (AVPS(Zhou et al., 2020)) and the reward signature (RS (Speer et al., 2022)) To explore if signals in this network covary with PES or PEM we then simply dot-multiplied the PES or PEM parameter estimate volume for each participant separately with the AVPS and RS, after having brought the AVPS and RS into our fMRI analysis space using ImageCalc. The result of the dot-multiplication indicates how much the covariance with PES or PEM loads on the AVPS or RS. We then brought these values into JASP, and compared them against zero and correlated them with wf. Because the loadings were normally distributed, we used parametric analyses. Second, we performed a similar analysis at the voxel level, by bringing the parameter estimate images for PES and PEM into a second level linear regression using a constant and wf as the two predictors. A t-test on the constant then reveals regions in which signals covary with PES or PEM after removing variance explained by wf. A t-test on the wf parameter estimate then reveals regions where the signals covary with PES or PEM in ways that depend on wf. To test if signals covary with 1-wf, we simply used a negative contrast in the t-test. Results were familywise corrected at the cluster level using the established two-step procedure in SPM: (i) for cluster-cutting we visualized results at punc < .001 k=10, and identified the FWEc minimum cluster size for family wise error correction from the results table, (ii) we reloaded the results at punc < .001 k=FWEc, so that all displayed results survive FWE at cluster-level. The same was done for EVS and EVM, but only reported in Supplementary Figure 18. For contrasts not revealing significant clusters at that threshold, we also mention results that were cluster-cut at punc < 0.01, and then applied the FWEc that SPM calculates at that cluster-cutting threshold. However, it should be noted that cluster-extent corrections following such permissive cluster-cutting thresholds are more subject to false positives and should be interpreted with care(Eklund et al., 2016).

Effect(s) tested: see above

Specify type of analysis: ☒ Whole brain ☐ ROI-based ☐ Both

Statistic type for inference (See Eklund et al. 2016): Results were familywise corrected at the cluster level using the established two-step procedure in SPM: (i) for cluster-cutting we visualized results at punc < .001 k=10, and identified the FWEc minimum cluster size for family wise error correction from the results table, (ii) we reloaded the results at punc < .001 k=FWEc, so that all displayed results survive FWE at cluster-level. The same was done for EVS and EVM, but only reported in Supplementary Figure 18. For contrasts not revealing significant clusters at that threshold, we also mention results that were cluster-cut at punc < 0.01, and then applied the FWEc that SPM calculates at that cluster-cutting threshold. However, it should be noted that cluster-extent corrections following such permissive cluster-cutting thresholds are more subject to false positives and should be interpreted with care(Eklund et al., 2016).

Correction: FWEc

## Models & analysis

| n/a | Involved in the study |
|---|---|
| ☒ | ☐ Functional and/or effective connectivity |
| ☒ | ☐ Graph analysis |
| ☐ | ☒ Multivariate modeling or predictive analysis |

Multivariate modeling and predictive analysis: To improve reverse inference, we used two multivariate signatures the affective vicarious pain signature (AVPS(Zhou et al., 2020)) and the reward signature (RS (Speer et al., 2022)) To explore if signals in this network covary with PES or PEM we then simply dot-multiplied the PES or PEM parameter estimate volume for each participant separately with the AVPS and RS, after having brought the AVPS and RS into our fMRI analysis space using ImageCalc. The result of the dot-multiplication indicates how much the covariance with PES or PEM loads on the AVPS or RS. We then brought these values into JASP, and compared them against zero and correlated them with wf. Because the loadings were normally distributed, we used parametric analyses.
